# PbsNRs: predict the potential binders and scaffolds for nuclear receptors

**DOI:** 10.1093/bib/bbae710

**Published:** 2025-01-11

**Authors:** Genhui Zheng, Dingfeng Wu, Xiuxia Wei, Dongpo Xu, Tiantian Mao, Deyu Yan, Wenhao Han, Xiaoxiao Shang, Zikun Chen, Jingxuan Qiu, Kailin Tang, Zhiwei Cao, Tianyi Qiu

**Affiliations:** Institute of Clinical Science, Zhongshan Hospital, Shanghai Medical College, Shanghai Institute of Infectious Disease and Biosecurity, Intelligent Medicine Institute, School of Life Sciences, Fudan University, No. 180 Fenglin Road, Shanghai 200032, China; Oden Institute for Computational Engineering and Sciences (ICES), University of Texas at Austin, No. 201 East 24th Street, Austin 78712, TX, United States; National Center, Children’s Hospital, Zhejiang University School of Medicine, National Clinical Research Center for Child Health, No. 3333 Binsheng Road, Hangzhou 310052, China; School of Health Science and Engineering, University of Shanghai for Science and Technology, No. 516 Jungong Road, Yangpu District, Shanghai 200093, China; School of Health Science and Engineering, University of Shanghai for Science and Technology, No. 516 Jungong Road, Yangpu District, Shanghai 200093, China; School of Life Sciences and Technology, Tongji University, No. 1239 Siping Road, Shanghai 200092, China; School of Life Sciences and Technology, Tongji University, No. 1239 Siping Road, Shanghai 200092, China; School of Life Sciences and Technology, Tongji University, No. 1239 Siping Road, Shanghai 200092, China; Institute of Clinical Science, Zhongshan Hospital, Shanghai Medical College, Shanghai Institute of Infectious Disease and Biosecurity, Intelligent Medicine Institute, School of Life Sciences, Fudan University, No. 180 Fenglin Road, Shanghai 200032, China; Department of Mathematics and Statistics, McGill University, 805 Sherbrooke Street West, Montreal H3A 0B9, Quebec, Canada; School of Life Sciences and Technology, Tongji University, No. 1239 Siping Road, Shanghai 200092, China; School of Health Science and Engineering, University of Shanghai for Science and Technology, No. 516 Jungong Road, Yangpu District, Shanghai 200093, China; School of Life Sciences and Technology, Tongji University, No. 1239 Siping Road, Shanghai 200092, China; Institute of Clinical Science, Zhongshan Hospital, Shanghai Medical College, Shanghai Institute of Infectious Disease and Biosecurity, Intelligent Medicine Institute, School of Life Sciences, Fudan University, No. 180 Fenglin Road, Shanghai 200032, China; Institute of Clinical Science, Zhongshan Hospital, Shanghai Medical College, Shanghai Institute of Infectious Disease and Biosecurity, Intelligent Medicine Institute, School of Life Sciences, Fudan University, No. 180 Fenglin Road, Shanghai 200032, China

**Keywords:** nuclear receptors, computer-aided drug design, bioinformatics, PCM model, molecule scaffold

## Abstract

Nuclear receptors (NRs) are a class of essential proteins that regulate the expression of specific genes and are associated with multiple diseases. *In silico* methods for prescreening potential NR binders with predictive binding ability are highly desired for NR-related drug development but are rarely reported. Here, we present the PbsNRs (Predicting binders and scaffolds for Nuclear Receptors), a user-friendly web server designed to predict the potential NR binders and scaffolds through proteochemometric modeling. The utility of PbsNRs was systemically evaluated using both chemical compounds and natural products. Results indicated that PbsNRs achieved a good prediction performance for chemical compounds on internal (ROC-AUC = 0.906, where ROC is Receiver-Operating Characteristic curve and AUC is the Area Under the Curve) and external (ROC-AUC = 0.783) datasets, outperforming both compound–ligand interaction tools and NR-specific predictors. PbsNRs also successfully identified bioactive chemical scaffolds for NRs by screening massive natural products. Moreover, the predicted bioactive and inactive natural products for NR2B1 were experimentally validated using biosensors. PbsNRs not only aids in screening potential therapeutic NR binders but also helps discover the essential molecular scaffold and guide the drug discovery for multiple NR-related diseases. The PbsNRs web server is available at http://pbsnrs.badd-cao.net.

## Introduction

Nuclear receptors (NRs) are transcription factors that recognize steroid hormones, thyroid hormones, and certain other molecules, playing a crucial role in drug development by directly combining with DNA and regulating gene expression. As one of the most abundant transcriptional regulators, NRs are involved in metabolism and are closely related to the occurrence, development, and prognosis of various essential diseases, including breast cancer [1–3], castration-resistant prostate cancer [4–6], liver diseases [7–9], type 2 diabetes [10, 11], and dyslipidemia [12, 13]. Currently, 48 NRs have been identified in human cells [14], and ~16% of all U.S. Food and Drug Administration (FDA)-approved small molecule drugs target NRs [15]. Thus, therapeutic drug development targeting NRs presents a continuous opportunity for addressing NR-related metabolic diseases. Additionally, discovering a new bioactive scaffold can lead to the identification of a new compound class with the potential to become future drugs [16–18]. High-throughput screening approaches are particularly valuable for NR-related drug development. Over the past decades, computer-aided drug design has proven to be a successful method for drug development [19]. For instance, the quantitative structure–activity relationship (QSAR) approach is widely used for *in silico* drug discovery by linking molecular information with bioactivity data through various quantitative functions [20]. Traditional QSAR methods simplify the drug–target interaction to compound–protein interaction (CPI) and predict whether the given compound is a ligand of the given protein. However, traditional QSARs only consider interactions between multiple compounds and a single protein target; lacking information on the target side makes it difficult to describe the binding patterns between compounds and multiple proteins from the same family [21]. Proteochemometric modeling (PCM), which integrates the information from both ligands and targets, was introduced [22]. Derived from QSAR, PCM is a modeling technique for bioactivity prediction. The key component is that both the characteristics of ligands and proteins should be considered. In the PCM model, the combination of ligand descriptors and protein descriptors is utilized as the input of machine learning model [22–24]. Till now, PCM modeling has been widely used to study molecular recognition mechanism in various contexts, including human immunodeficiency virus (HIV) protease variants [25], G protein–coupled receptors [26, 27], kinases [28, 29], enzymes [30, 31], and histone deacetylases [32].

Several approaches have been reported for predicting potential CPIs, including matrix factorization methods [33], bipartite local model [34], network-based inference [35], and network integration [36]. Docking methods such as ZDock [37] and AutoDock [38] also play a significant role in drug identification and development. Additionally, deep learning–based approaches, including convolutional neural networks and graph neural networks, have improved the performance and efficiency of drug screening [39–43]. These approaches primarily rely on graph theory and network rather than the physical and chemical properties of potential compounds, mainly focusing on common protein targets with bioactive data derived from databases such as Yamanishi [44], ChEMBL (https://www.ebi.ac.uk/chembl/) [45], DrugBank [46], and Human Protein Reference Database [47]. However, none of these approaches are specifically designed for NR proteins. Recently, two algorithms, NR-Profiler [48] and NR-ToxPred [49], have been developed to predict the bioactive compounds for NRs. NR-Profiler, based on the collected data, utilizes multitask deep learning to predict binders, weak binders, and nonbinders for NRs. Similarly, NR-ToxPred predicts binders for NRs based on Nuclear receptor activity (NURA) dataset, contributing to drug development of NR-related diseases [50]. However, both algorithms have limited scopes, focusing on only a few NR targets, which restricts their use in NR-related drug developments. Therefore, it is urgent to develop an approach concentrating on NRs based on a learning-guided *in silico* model. In this article, we present predicting binders and scaffolds for nuclear receptors (PbsNRs), a new computational tool designed to predict the potential binders and scaffolds for NRs through PCM modeling. The main objective of PbsNRs is to introduce the sequence similarity features across different NRs, allowing the model to be trained on the NRs with bioactive data and expanded to other NR targets lack of experimental data. To achieve this, we collected 6554 bioactive compounds with corresponding EC_50_ (half maximal effective concentration) values against five NR targets from the orphan nuclear receptor ligand binding database (ONRLDB) [51] as a modeling dataset for feature selection, parameter optimization, and internal validation. Additionally, to evaluate the performance of transfer learning, we used 713 independent bioactive compounds for six other NRs outside the modeling dataset as external validation. The prediction performance of PbsNRs was rigorously validated and compared with state-of-the-art tools, including CPI methods and NR-specific methods, demonstrating superior performance for prediction accuracy and application scope. PbsNRs was also applied for large-scale screening of chemical compounds and nature products through ChEMBL [45], Drugbank [46], and TCM@Taiwan [52], illustrating that the natural products might be a good screening library for NR ligands. Finally, we screened the NR2B1 ligands from the natural compounds database of herbal ingredients’ targets database (HIT 2.0) [53] and natural product activity and species source (NPASS) [54] through PbsNRs, selecting seven untested compounds for evaluation. The binding ability of these seven compounds to NR2B1 showed 100% agreement between the *in silico* prediction and experiments using biosensors.

## Materials and methods

### Descriptor generation

The PbsNRs model was generated based on bioactivity data for five NRs and validated using both the internal dataset and the external dataset (see Data Collection in Supplementary Methods). Detailed data distribution can be found in Supplementary Table 1. The model requires descriptors for both chemical compounds and NRs. For each chemical compound, the chemical and physical properties are described through RDKit, as previously reported [55]. Detailed information on chemical descriptors is listed in Supplementary Table 2. Further, for model construction, the structure information of all NRs involved in ONRLDB was collected from the protein data bank (PDB) [56] as potential targets, referred to as background NRs (Supplementary Table 3). Other NRs with crystal structures but not involved in ONRLDB are also included in the online version, referred to as extended NR targets. For each NR target, the similarity compared with 30 NRs is calculated by Smith–Waterman alignment [57], generating a 30-dimensional protein descriptor. Detailed information on protein descriptors is listed in Supplementary Table 4. The description of model optimization can be found in the Model Optimization part of Supplementary File*.* Detailed parameters for deep learning and random forest (RF) model optimization can be found in Supplementary Tables 5 and 6.

### Algorithm design of PbsNRs

**Figure 1:**
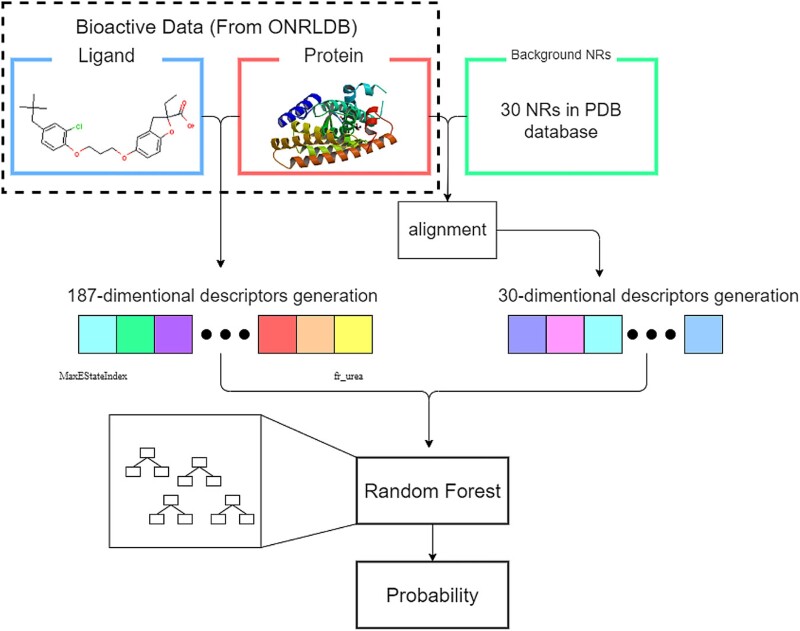
Illustration of PbsNRs modeling. The input data involve both the ligand structure of the molecule and the protein structure of NR. For each pair, 187-D descriptors for the ligand and 30-bit descriptors for the protein target are generated. Combined with bio-active data, the PCM modeling can be generated through machine learning approaches of random forest and deep learning. Finally, the PbsNRs score for each pair can be calculated.

The overall algorithm of PbsNRs is presented in Fig. 1. The PbsNRs server contains an NR library containing 30 NR targets with crystalized structure information. For any NR target, the similarity score across all 30 NR targets in the NR library is calculated to generate the protein similarity descriptors. For each compound with a chemical structure, a series of fingerprints are generated to describe the structural and physical–chemical features of the compounds. Finally, a total number of 217-dimensional (30 for NR and 187 for compound) descriptors is generated for model construction. Besides the RF classifier, the deep learning approach was also selected for model construction and comparison. The number of input neurons is 217, and the number of output neurons is 1. After parameter optimization, the activation function used in the DL approach is defined as ReLU. The number of hidden modules and neurons is 5 and 300, respectively, with a dropout rate of 0.5. The total number of parameters is 430 636, listed in Supplementary Table 7. The output activity score of PbsNRs represents the probability score, ranging from 0 to 1. The threshold of this score is defined at two levels: activity score >0.5 or >0.7. A higher score represents a higher probability of the ligand becoming the potential ligand of the corresponding target. In the PbsNRs web server, activity scores over 0.7 are marked in green, scores between 0.5 and 0.7 are marked in orange, and scores lower than 0.5 are marked in red.

By setting the different sizes of 6554 bioactivity data as training datasets, multiple models were constructed based on 50%–90% of the total training dataset with a 5% interval. Considering the imbalance between positive and negative samples in our training dataset, we utilized oversampling, a commonly used strategy in the machinery learning method [58]. At the same time, Gaussian noise was added to increase the robustness of our model [59]. For each NR target, oversampling in training was set from 1000 to 5000 with 500 intervals, and Gaussian noise was set as sigma = 0.1. The PbsNRs was evaluated on multiple models (see Model Optimization in Supplementary Methods part). After evaluation (see Results), the best model of PbsNRs was generated by RF classifier by setting the training percentage and oversampling number as 85% and 5000, respectively.

## Results

### Evaluation of PbsNRs modeling

**Figure 2:**
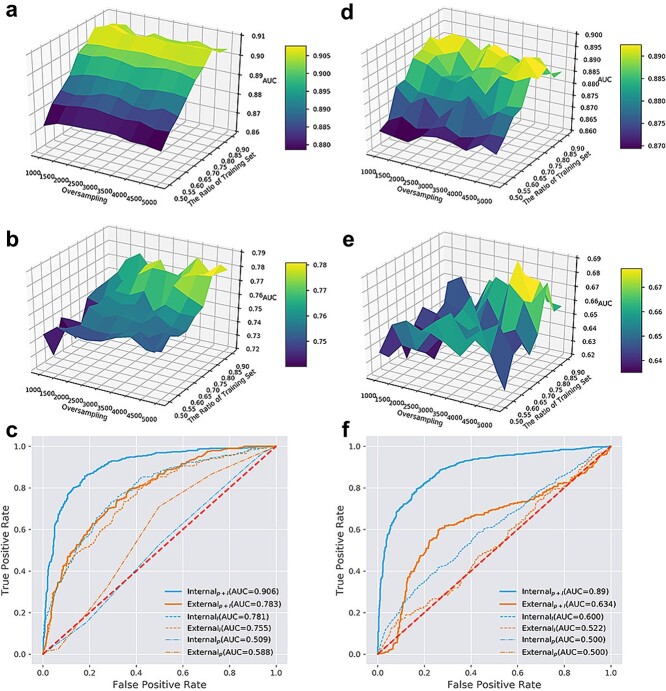
Model performance for random forest and deep learning. (a) Model performance of random forest (RF) on the internal validation set. (b) Model performance of RF on external set. (c) ROC curve of RF on both internal and external sets with 0.85 training set percentage and 5000 oversampling number. The subscripts of different labels represent models constructed on different descriptors but evaluated on the corresponding dataset. For example, external_p + l_ represents the performance of the model based on protein and ligand descriptors on the external dataset. (d) Model performance of DL on the internal set. (e) Model performance of DL on the external set. (f) ROC curve of DL on both internal and external sets with 0.80 training set percentage and 4000 oversampling number. The subscripts of different labels represent models constructed on different descriptors but evaluated on the corresponding dataset.

The performance of PbsNRs constructed by optimized RF classifier and deep learning was evaluated through both internal and external validation (Fig. 2). For internal testing, both approaches were evaluated through different training sizes with oversampling and noise (see Materials and Methods). The best model of each approach with defaulted parameters was further evaluated through the external testing set. It was found that increasing the percentage of the training set and oversampling number smoothly increased the performance of the RF classifier for internal evaluation (Fig. 2a). For external validation, the increasing number of training sets and oversampling significantly but not smoothly increased the performance for classification (Fig. 2b). The optimized RF model achieved ROC-AUC values of 0.906 on the internal validation set and 0.783 on the external validation set (Fig. 2c). On the other hand, the performance of DL approximately followed the principle of rough but monotonic increase on the internal validation (Fig. 2d) and showed random performance in the external validation (Fig. 2e). The optimized DL model achieved ROC-AUC values of 0.890 on the internal validation set and 0.634 on the external validation set (Fig. 2f). During parameter optimization, RF demonstrated better robustness by providing stable performance with a relatively smooth monotonic increasing surface, while DL showed unstable performance. Besides ROC-AUC, under optimized parameters, RF achieved better performance than DL on other parameters, including accuracy, precision, recall, and F1-score (Table 1).

**Table 1:** Performance of random forest and deep learning.

Internal	Accuracy	Precision	Recall	F1-score
RF	0.835	0.833	0.847	0.840
DL	0.815	0.877	0.900	0.834
**External**	**Accuracy**	**Precision**	**Recall**	**F1-score**
RF	0.72	0.756	0.794	0.774
DL	0.668	0.714	0.745	0.729

We also tested the performance of different combinations of protein and ligand descriptors, including: (i) ligand descriptors (L), (ii) protein descriptors (P), and (iii) the combination of protein and ligand descriptors (P + L). The ROC-AUC values of models based on different descriptors and established by RF (Fig. 2c) and DL (Fig. 2f) illustrated that the protein descriptors alone gave a low prediction performance of 0.500–0.588, only slightly better than random selection (~0.5). For ligand descriptors alone, the performance of RF reached a tolerable performance of ~0.755–0.781, better than the DL results. The combined descriptors of protein and ligand significantly increased prediction performance to ~0.9 for both RF and DL in the internal validation dataset. For external validation, the performance of RF can also reach a tolerable level of 0.783. The results showed that combined descriptors could provide good prediction performance, outperforming single-side descriptors, and extend the application scope of PbsNRs from NRs with bioactive data to NRs without bioactive data.

In addition, we predicted all compounds in the external dataset through the RF classifier and normalized the predicted score from 0 to 1. The prediction scores showed a significant difference (*P* < .0001) between the experimentally validated bioactive group (mean = 0.685, median = 0.801, and standard deviation of 0.224) and the inactive group (mean = 0.428, median = 0.453, and standard deviation of 0.249), indicating the outstanding performance of PbsNRs modeling (Supplementary Fig. 2).

### Performance comparison between PbsNRs and other state-of-the-art tools

PbsNRs’ performance was compared with available state-of-the-art tools using an independent testing dataset. The prediction between NRs and compounds falls into the spectrum of the CPI prediction. Thus, we selected two DL-based CPI approaches, Tsubaki’s model [41] and DeepCPI [42], and two tailor-made NR–ligand predictors, NR-ToxPred [49] and NR-Profiler [48] for comparison.

**Figure 3:**
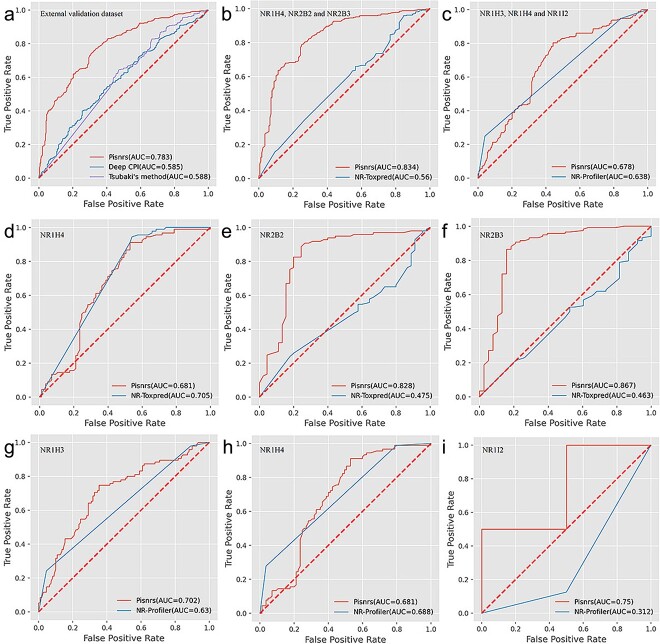
Model performance for PbsNRs and state-of-the-art tools. (a) The ROC curve of PbsNRs, Tsubaki’s method, and DeepCPI on the external dataset. (b) The ROC curve of PbsNRs and NR-ToxPred on NR1H4, NR2B2, and NR2B3. (c) The ROC curve of PbsNRs and NR-Profiler on NR1H3, NR1H4, and NR1I2. (d) The ROC curve of PbsNRs and NR-ToxPred on NR1H4. (e) The ROC curve of PbsNRs and NR-ToxPred on NR2B2. (f) The ROC curve of PbsNRs and NR-ToxPred on NR2B3. (g) The ROC curve of PbsNRs and NR-Profiler on NR1H3. (h) The ROC curve of PbsNRs and NR-Profiler on NR1H4. (i) The ROC curve of PbsNRs and NR-Profiler on NR1I2.

The external dataset was used for a fair comparison between PbsNRs, Tsubaki’s model, and DeepCPI (see Model Comparison and Evaluation Parameters part in Supplementary Methods). Results showed that PbsNRs achieved an ROC-AUC value of 0.783, significantly higher than Tsubaki’s model (0.588) and DeepCPI (0.585) (Fig. 3a). For applicable tools, the threshold to distinguish between bioactive compounds and inactive ones should be defined. We defined two types of cutoffs for comparison: the first cutoffs were those on the ROC curves to achieve the best-balanced accuracy (BA), and the second ones were defined as 0.5 for all three tools. Based on the two types of cutoffs, model performance, including precision, recall, accuracy, false-positive rate (FPR), and F1-score, are listed in Table 2. Results showed that PbsNRs achieved the best score of accuracy, precision, and F1-score on both cutoffs, with only a lower recall rate on cutoff = 0.5 compared to the other two CPI tools. This is because the two CPI tools labeled the majority of the compounds as active ones based on the 0.5 cutoffs, leading to an extremely high FPR of 0.821 for Tsubaki’s model and an FPR of 0.747 for DeepCPI. The results showed that PbsNRs provided accurate predictions with lower FPR than the other two CPI approaches for NR binder prediction, indicating that the CPI features might be different from NR–ligand interactions and other protein–compound interactions.

**Table 2:** Performance of three different tools based on two types of cutoffs.

**Tools**—**CPI**	**Accuracy**	**Precision**	**Recall**	**F1-score**	**FPR**
PbsNRs—best BA^a^	**0.722**	**0.783**	**0.743**	**0.763**	0.309
Tsubaki’s model—best BA	0.576	0.672	0.645	0.658	0.474
DeepCPI—best BA	0.537	0.701	0.400	0.509	**0.256**
PbsNRs—0.5^b^	**0.729**	**0.761**	0.801	**0.780**	**0.379**
Tsubaki’s model—0.5	0.626	0.628	**0.923**	0.747	0.821
DeepCPI—0.5	0.607	0.629	0.843	0.721	0.747
Tools—NR–ligand predictor	**Accuracy**	**Precision**	**Recall**	**F1-score**	**FPR**
PbsNRs—best BA	**0.774**	**0.851**	**0.787**	**0.818**	**0.250**
NR-Toxpred—best BA	0.672	0.673	0.957	0.790	0.845
PbsNRs—best BA	**0.679**	0.638	0.803	0.711	0.442
NR-Profiler—best BA	0.610	**0.857**	0.249	0.386	**0.040**

Tools labeled with best BA means the cutoff of defined as those to achieve the best balanced accuracy (BA).

^a^Best BA means the cutoff of each model was defined as those to achieve the best BA.

^b^0.5 means the cutoff of each model was defined as 0.5.

Besides CPI tools, we compared PbsNRs with NR-ToxPred [49] and NR-Profiler [48]. Considering that both methods only apply to a limited number of NR families, the corresponding NRs from the external dataset were separately selected for validation, including NR1H4, NR2B2, and NR2B3 for NR-ToxPred, and NR1H3, NR1H4, and NR1I2 for NR-Profiler (Supplementary Table 8). Results showed that PbsNRs achieved an AUC value of 0.834 on three validation NRs of NR-ToxPred, significantly higher than NR-ToxPred (0.56) (Fig. 3b). For three validation NRs of NR-Profiler, PbsNRs achieved a comparable AUC of 0.678, higher than NR-Profiler (0.638) (Fig. 3c). PbsNRs achieved similar prediction performance on NR1H4 (Fig. 3d) and significantly better performance on NR2B2 (Fig. 3e) and NR2B3 (Fig. 3f) compared to NR-ToxPred. Similarly, PbsNRs achieved a better AUC value on NR1H3 (Fig. 3g) and comparable performance on NR1H4 (Fig. 3h) compared to NR-Profiler. Due to only 12 experimentally validated ligands binding to NR1I2 in our external dataset, the performance of AUC = 0.75 for PbsNRs and AUC = 0.312 for NR-Profiler (Fig. 3i) is biased and cannot prove significantly better performance for our model than NR-Profiler. We also calculated accuracy, precision, recall, F1-score, an FPR under the best cutoff for PbsNRs, and the other two NR–ligand predictors (Table 2). Results showed that PbsNRs achieved better performance on all parameters against NR-ToxPred and obtained lower precision and a higher FPR against NR-Profiler (Table 2).

Further, we evaluated the prediction scores between positive samples and negative samples in our external validation dataset of NR1H4, NR2B2, and NR2B3 for NR-ToxPred, and NR1H3, NR1H4, and NR1I2 for NR-Profiler. It can be found that PbsNRs scores could significantly distinguish positive ligands from negative ones with high statistical significance on NR-ToxPred external validation datasets (Supplementary Fig. 3A) and the NR-ToxPred external validation datasets dataset (Supplementary Fig. 3B). As a classification model, NR-Profiler gives only the 0 and 1 solutions with statistical significance (Supplementary Fig. 3C), but NR-ToxPred gives a similar scoring interval between positive ones and negative ones with mean and median over 0.8 (3d). All the above demonstrated that PbsNRs outperformed both CPI models and NR–ligand predictors on the external dataset.

### Applying PbsNRs on chemical compounds and natural products

**Figure 4:**
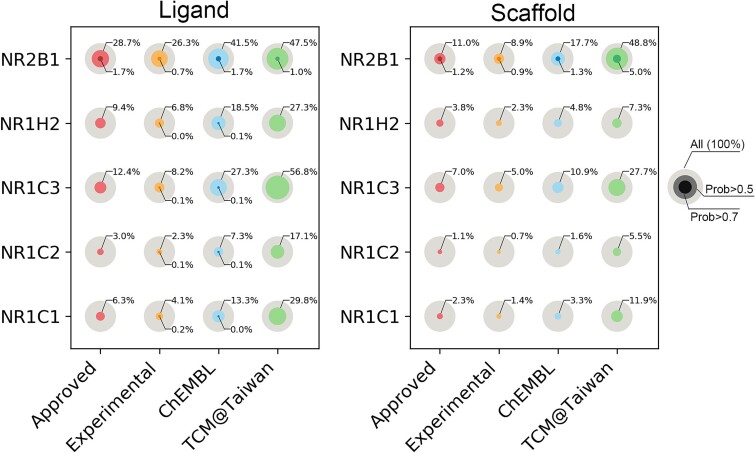
Evaluation of four different datasets. Testing dataset including Drug bank Approved, Drug bank Experimental, ChEMBL, and TCM@taiwan. The gray circle represents all compounds in one dataset. The transparent-colored circles represent bioactive chemicals with predicted values over 0.5 and the solid-colored circles represented those with values over 0.7.

To extend its application scope, PbsNRs were applied to both chemical compounds and natural products by screening the database of ChEMBL [45], Drugbank [46], and TCM@Taiwan [52]. The potential binding scores of the compounds in these databases against five major NRs (NR2B1, NR1H2, NR1C3, NR1C2, and NR1C1) were calculated through the PbsNRs algorithm. Considering the massive number of compounds in ChEMBL, only 5000 chemical structures were randomly selected [45]. For Drugbank, the compounds were divided into FDA-approved drugs and experimental ones. PbsNRs defined two levels of cutoffs: normal level (level 1: >0.5 as positive) and strict level (level 2: >0.7 as positive). The proportion of compounds over the cutoff is illustrated in Fig. 4.

As a benchmark, the proportions of potential ligands with prediction scores over 0.5 in FDA-approved drugs are higher than experimental ones, indicating the higher druggability of FDA-approved drugs to become potential NR binders. Interestingly, the TCM@Taiwan database, containing natural products, showed significantly higher proportions of ligands with predicted scores over 0.5 on 5 NRs (17.1%–47.5%) compared to the other two databases, followed by ChEMBL (7.3%–41.5%) and FDA-approved (3.0%–28.7%). The percentage of bioactive scaffolds in three databases showed a similar phenomenon, with natural products showing a higher proportion of bioactive scaffolds. The results indicated that natural products hold potential as bioactive ligands targeting NRs. Previous research reported lower cytotoxicity for natural products [60–62], suggesting that they might be a good library for screening NR-related drugs [63].

**Figure 5:**
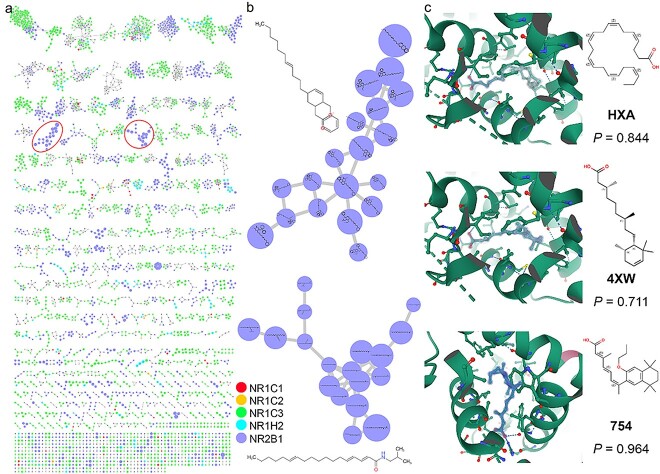
MMPs’ chemical space of natural products. (a) MMPs chemical space of all the natural products derived from TCM Database@Taiwan. (b) Two bioactive clusters for NR2B1. Five corresponding bioactive target with the highest score were presented in here. In addition, the detailed structure of the compound with the highest predicted values in the cluster is shown nearby. (c) Three validated examples are shown. The left side indicates how they interacted with the targeted proteins (Mol*). The right side indicates their corresponding structure, abbreviation, and predicted value.

We applied PbsNRs to natural products to detect potential binders and scaffolds of NR targets from 35 941 natural compounds derived from TCM Database@Taiwan [52]. The binding ability score for all natural compounds was predicted by PbsNRs. The matched molecular pair (MMP) identification method [64] was introduced to identify all MMPs in these natural compounds (see MMPs Generation and Molecular Scaffold Searching Part in Supplementary Methods). The result is illustrated in Fig. 5a. Each node represents a compound with a color indicating one of the NRs with the maximum predicted score. For example, a red node indicates a bioactive compound for NR1C1. As illustrated in Fig. 5a, most compounds are bioactive for NR1C3 and NR2B1, with two clusters of NR2B1 zoomed in Fig. 5b. These predicted bioactive compounds for NR2B1 have long chains, providing flexibility to embed into specific NR binding domains. Among them, doconexent [65] (in the PDB database, the ligand id is 1MV9), 9-*cis*-13,14-dihydroretinoic acid [66] (PDB ligand id: 4ZSH), and (2E,4E,6Z)-3-methyl-7-(5,5,8,8-tetramethyl-3-propoxy-5,6,7,8-tetrahydronaphthalen-2-yl) octa-2,4,6-trienoic acid [67] (PDB ligand id: 6STI) have been proven as bioactive compounds for NR2B1. Detailed molecular structures are illustrated in Fig. 5c using Mol* [68]. DHA is FDA-approved for mitigating obesity [69], promoting sleep in preterm toddlers [70], and preventing diseases in older age [71]. All have a carboxy group, commonly interacting with arginine through salt bridges, suggesting that long-chain scaffolds with carboxy groups hold potential for NR2B1 [72–74]. The above results indicate PbsNRs’ potential to excavate NR-related binders and scaffolds from natural products.

### Experimental validation on the virtual screening ligands of NR2B1

**Figure 6:**
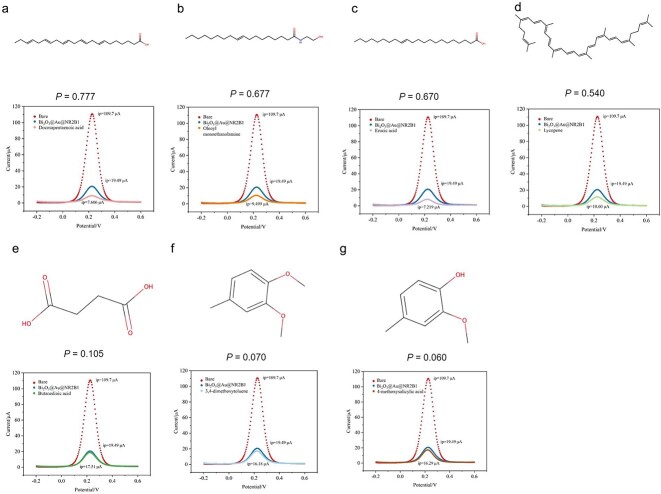
Experimental validation on NR2B1. Each subfigure represents the results of one specific natural compound, including compound structure, prediction score between NR2B1 and the compound, cyclic voltammetry (CV) assay curve of the biosensor. (a) Experimental results between NR2B1 and docosapentaenoic acid. (b) Experimental results between NR2B1 and oleoyl monoethanolamine. (c) Experimental results between NR2B1 and erucic acid. (d) Experimental results between NR2B1 and lycopene. (e) Experimental results between NR2B1 and butanedioic acid. (f) Experimental results between NR2B1 and 3,4-dimethoxytoluene. (g) Experimental results between NR2B1 and 4-methoxysalicylic acid.

Retinoic acid receptor RXRα, also known as NR2B1, is essential for retinoid drugs such as acitretin to treat severe psoriasis, adapalene to treat acne vulgaris, and alitretinoin to treat chronic hand eczema and psoriasis. Currently, only 12 drugs related to NR2B1 are FDA-approved [46]. We aimed to discover potential NR2B1 binders for drug discovery to evaluate PbsNRs’ utility. The target protein of NR2B1 was screened across HIT 2.0 [53] and NPASS [54] to detect potential NR2B1 binders through natural compounds. Seven compounds, including four positive ones: docosapentaenoic acid (score = 0.777), oleoyl monoethanolamine (score = 0.677), erucic acid (score = 0.670), and lycopene (score = 0.540), and three negative ones: butanedioic acid (score = 0.105), 3,4-dimethoxytoluene (score = 0.070), 4-methoxysalicylic acid (score = 0.060), were tested through biosensor validation (see Construction and Validation of Biosensors for NRs’ Ligands Detection in Supplementary Methods part). Pre-experiments illustrated the clear separation of redox peaks between the peaks of [Fe(CN)6] 3-/4- (Supplementary Fig. 4) and demonstrated the EIS curves of Bi2O3@Au, Bi2O3@Au@NR2B1, and bare electrodes (Supplementary Fig. 5). As is shown in Fig. 5 & Table 3, the signal peak of the control group which only added the NR2B1 without ligands was 19.49 μA. After adding the predicted positive natural compounds, the signal peak decreased to 7.606 μA for docosapentaenoic acid (Fig. 6a), 9.499 μA for oleoyl monoethanolamine (Fig. 6b), 7.219 μA for erucic acid (Fig. 6c), and 10.60 μA for lycopene (Fig. 6d), respectively. These results demonstrated that adding the above four predicted positive compounds could increase the resistance and decrease the peak of the signal by 1.8–2.7 times, indicating the NR2B1 protein on the surface of the biosensor could bind with the above four compounds, causing the changes in its spatial conformation, therefore increasing the amount of coverage on the electrode surface and impeding electron transfer. Meanwhile, adding the predicted negative ones could slightly change the peak of the signal from 19.49–17.51 μA for butanedioic acid (Fig. 6e), 16.18 μA for 3,4-dimethoxytoluene (Fig. 6f), and 16.29 μA for 4-methoxy salicylic acid (Fig. 6g), respectively. This result indicates almost no changes in the peak of signal, which decreased only 10% ~ 17% compared with the control. Given the above result, it’s shown that the prediction of PbsNRs highly corresponds with the experimental validation, which shows its utility of drug development for NRs. In addition, the predicted compounds have different structure features. For example, the positive compounds of docosapentaenoic acid, oleoyl monoethanolamine and erucic acid, have long chains and a similar scaffold. Also, the negative compounds of 3,4-dimethoxytoluene and 4-methoxysalicylic acid contain benzene rings. The above results also showed that the algorithm of PbsNRs has the potential for scaffold identification.

**Table 3:** Prediction results of PbsNRs, AutoDock, CDDOCKER, and LibDock.

Molecules/Score	Experiment	PbsNRs	Autodock	CDDOCKER	LibDOCK
Docosapentaenoic acid	7.606 μA	0.777	−7.4	−24.442	116.49
Lycopene	10.60 μA	0.54	−7.68	−690.86	NAN
Erucic acid	7.219 μA	0.67	−5.97	20.079	121.09
Oleoyl monoethanolamine	9.499 μA	0.677	−6.28	26.55	112.12
3,4-Dimethoxytoluene	16.18 μA	0.07	−4.41	14.751	45.88
4-Methoxysalicylic acid	16.29 μA	0.06	−3.52	17.364	50.11
Butanedioic acid	17.51 μA	0.105	−2.77	40.925	47.8
Running time (seconds)		<1	574	1128	416

We compared the prediction results of the seven molecules against NR2B1 with state-of-the-art Docking approaches (AutoDock [38], CDDOCKER [75], and LibDOCK [76]). AutoDock provided similar prediction results consistent with the experimental results (Table 3). Meanwhile, the calculated energy of CDDOCKER was 20.079 for erucic acid and 26.55 for oleoyl monoethanolamine, which were higher than those in the negative compounds, indicating false-positive prediction results. On the other hand, LibDOCK could not find the potential docking sites for lycopene, which illustrated that lycopene could not been detected by LibDOCK. Moreover, we evaluated the running time for PbsNRs and three docking approaches on seven tasks and found that PbsNRs only need <1 s to finish the calculation, which was at least 400–1100 times faster than those of AutoDock (574 s), CDDOCKER (1128s), and LibDOCK (416 s). The above results showed that, compared with popular docking approaches, PbsNRs could not only provide accurate prediction but also significantly reduce the calculation time of virtual screening.

### Development of web server and Python package

**Figure 7:**
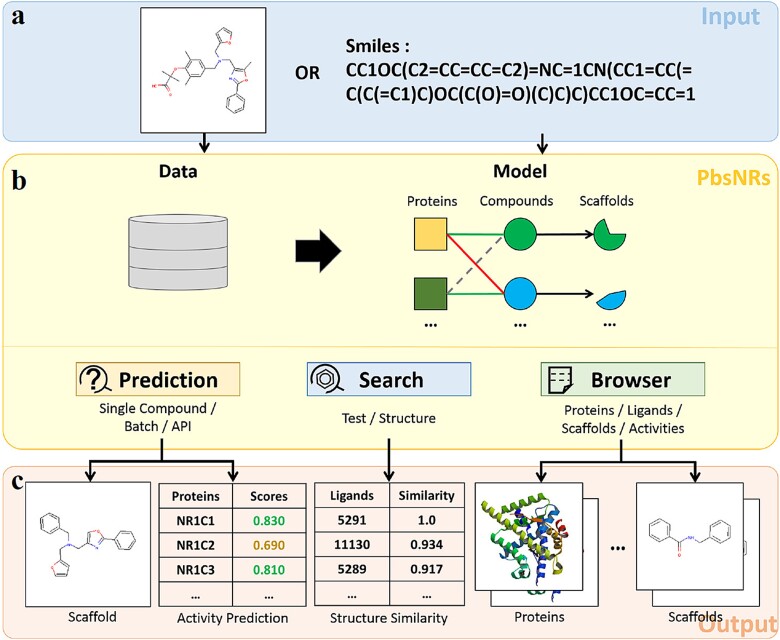
The operation flow of PbsNRs. (a) Input molecule structures in an accepted format. (b) After uploading, PbsNRs will automatically calculate the activity score through the PCM model against built-in NR targets. Besides prediction, PbsNRs also provide internal searching and a browser. (c) Output results of each function. For prediction, we provide three levels or activity score as high (0.7 ≤ score), median (0.5 ≤ score < 0.7), and low (score < 0.5).

The high performance of the algorithm and its application scope to detect potential NR binders justify constructing a user-friendly web server for PbsNRs (see Development of Web Server in Supplementary Methods), containing three major modules: (i) predict the activity score of query compounds against different NR targets, (ii) search the ligand molecules with a built-in database, and (iii) view information of the built-in database. A local Python implementation is provided, with the operation flow of PbsNRs illustrated in Fig. 7 and detailed descriptions in the PbsNRs implementation part of Supplementary Files.

A wrapper script was created and uploaded to GitHub (see Data and Code Availability) to facilitate compound prediction on the command line.

## Discussion

NRs could regulate the expression of specific genes and participate in several essential physiological processes, such as development, homeostasis, and metabolism of the organism [77, 78], making them promising drug targets for multiple systemic diseases. Given their unique sequence features, which allow direct binding with DNA, it is highly valuable to develop a specific model for NR-related drug prediction. Leveraging comprehensive data from ONRLDB [51], PbsNRs has been constructed as an efficient, accurate, and user-friendly web server designed for screening NR-related small molecule drugs. Further, independent validation indicated that PbsNRs outperformed both CPI tools and other NR-specific predictors. The PbsNRs modeling provided high precision (0.833) and recall (0.847) rates under the defaulted threshold for the internal dataset (Table 1). On the other hand, for the purpose of positive ligand selection, the FPR is an important metric for model evaluation (Table 2). Results showed that, under the cutoff of 0.5, PbsNRs could give the second-lowest FPR among all compared tools. These results represented that the model could be suitable for the prescreening of NR-related ligands, which could accelerate the potential drug design of NR-related drugs.

We evaluated the prediction scores of PbsNRs among positive and negative samples and found that the probability distribution of positive and negative compounds in the external dataset was significantly distinguishable. Most of the positive samples could provide a PbsNRs score over 0.5, while the scoring range of negative samples was lower than 0.6 (Supplementary Fig. 4). The prediction that PbsNRs could outperform the current SOTA tools may be due to two factors that other tools ignore. First, NR-Profiler utilizes the data record from international union of basic and clinical pharmacology (IUPHAR), NIMH psychoactive drug screening program (PDSP Ki), BindingDB (version 2019 m4), and ChEMBL. NR-ToxPred instead uses the NURA dataset as training data. Though the number of both datasets is greater than ONRLDB. However, they don’t cover a significant part of proteins in the NR family. Second, the choice of descriptors also leads to the key difference. Besides graph descriptors, NR-Profiler uses molecular fingerprints like PaDEL-Descriptor, molecular access system (MACCS), PubChem, Klekota-Roth, Morgan, and ECFP4 [79–81]. NR-ToxPred uses ECFP4 and MACCS fingerprints by using the RDkit package [81]. Though those fingerprints are useful in molecule representation, too many descriptors don’t lead to better performance in NR binder predictions.

This can be demonstrated by several case studies that PbsNRs succeed, while other methods fail. For example, compound 3-[4-(1-benzoylindol-2-yl)phenyl] propane nitrile can bind with Liver X receptor alpha (NR1H3) with an EC_50_ value of 0.012 [82] (Supplementary Fig. 6A). Meanwhile, according to the record in ONRLDB [51], ligands 3-[3-(8-chloro-3-methylquinolin-4-yl)phenoxy]-N-propylbenzamide and 3-{3-[3-benzyl-8-(trifluoromethyl)quinolin-4-yl]phenoxy}-N-propylbenzamide cannot bind to Liver X receptor alpha (Supplementary Fig. 6B and C). It can be found that PbsNRs provide scores of 0.832, 0.250, and 0.21, respectively, for the above three ligands, representing the consistent results between PbNRs and experimental validation. Meanwhile, the NR-profiler provided conflict results as nonbinder, binder, and binder.

Similar results can be found in the comparison between PbsNRs and NR-Toxpred; it can be found that ligand 4-[1-(1,1,2,3,3,6-hexamethyl-2H-inden-5-yl)ethenyl]benzoic acid can bind to Retinoid X receptor gamma (NR2B3) [83] (Supplementary Fig. 6D), while both 4-[(1-isopropyl-4,4,7-trimethyl-1,2,3,4-tetrahydroquinolin-6-yl)methylamino]benzoic acid and 2-(N-(5,5,8,8-tetramethyl-5,6,7,8-tetrahydronaphthalen-2-yl)methylsulfonamido)pyrimidine-5-carboxylic acid is unable to bind (Supplementary Fig. 6E and F). PbsNRs provide 0.900, 0.202, and 0.066, respectively, but NR-Toxpred gives 0.145, 0.839, and 0.963.

Besides chemical compounds from ONRLDB [51], PbsNRs were applied to natural products to discover potential NR binders. After high-throughput screening of 35 941 natural products, bioactive molecules were clustered based on structure similarity, identifying enriched molecular scaffold in each cluster. For instance, the enrichment group for NR2B1 in Fig. 5c suggests that long-chain scaffolds with a carboxy group could inhibit NR2B1 (RXRα) activity. This was further confirmed by experiments, where four model-predicted and biosensor-validated natural compounds contained long-chain structures. Thus, PbsNRs provide a new perspective for high-throughput screening of potential bioactive compounds for NRs. Interestingly, the predicted bioactive molecules for NRs share similar scaffolds, which could accelerate new drug development.

Key elements for PbsNRs modeling include ligand and target descriptors and appropriate learning approaches for training. In this study, ligand descriptors generated by RDKit comprehensively characterized the structure and physicochemical information of query molecules. For target descriptors, all 30 available background targets from NR families were used for modeling, rather than 11 NRs in our dataset. This approach improved the model’s extensibility and generalization ability [55]. In that case, the built-in target descriptor for PbsNRs was established based on 30 available background targets. Moreover, judging whether a ligand can interact with a selected target is crucial. Here, EC_50_ with a commonly used cutoff was used to define bioactive or inactive compounds. It shows that even if the activity value is converted to a classification label according to the threshold value of 1 μM, the model can still predict relatively accurate activity possibility according to the description information of protein and ligand. PbsNRs is a classification model, which predicts the small molecule binders of 30 NRs. The input is the SMILES of small molecules and specific NRs, and the output is the prediction score indicating the binding possibility.

In addition, numerical conversion from quantitative to classified data is a common method in data analysis, effectively eliminating noise from various sources and improving the model performance [84–86]. Our previous study showed that the RF classifier achieved the best prediction performance among several machine learning methods, including ridge classifier, logistic regression, support vector classification, and decision tree [55]. The RF classifier was further compared with the deep learning approach of the backpropagation neural network in this study. Results showed that RF outperformed DL in both prediction performance and generalization ability on internal and external datasets. This might be because the RF classifier, an ensemble of decision trees, randomly selects data and features to mitigate the overfitting problem of a single decision tree. In contrast, DL requires more data for model convergence and is more sensitive to noise and outliers. Experimental-based bioactive data may contain some noise, reducing DL prediction performance. Recent studies also indicated that tree-based models are more suitable for tabular data than DL approaches, aligning with the features derived in PbsNRs [87, 88]. Thus, PbsNRs’ web server was generated using an RF classifier. Additionally, oversampling and Gaussian noise were introduced to balance the ratio and improve the robustness of PCM modeling, addressing the imbalance of positive and negative samples in our training dataset. As a result, PbsNRs demonstrated good performance on the external dataset.

In summary, based on the extensive data of experimentally validated protein–ligand interactions between NRs and small molecules, we constructed an *in silico* model, PbsNRs for predicting potential binders for NRs. The results indicated the PbsNRs could provide good prediction performance (AUC = 0.906) for internal datasets and tolerable prediction performance (AUC = 0.783) for external datasets. In contrast, NR-Profiler and NR-ToxPred could not predict the ligands binding NRs outside the training dataset [48, 49]. This proves that PbsNRs have a better application range and extensibility. The part of reason is that we introduce PCM modeling rather than multitask QSAR for the first time in this field. Moreover, the tree-based model owns better prediction performance for tabular data such as the small-molecule fingerprint features from RDKit, which leads to the superior performance of PbsNRs compared with peers [87, 88]. Our results indicated that PbsNRs could not only successfully distinguish bioactive structures from background compounds but also provide bioactive molecular scaffolds to guide new drug discovery, assisting in developing NR-related pharmaceuticals.

Also, we detected the limitations of our method, in which PbsNRs may tend to provide similar predictions for the same or similar scaffold. It should be noted that the scaffold represents the structure features of a series of compounds, but sometimes, similar scaffolds may result in opposite bioactivity. This suggests that the subsequent direction of improvement may need to incorporate a more precise description of the molecules, such as the description of functional groups other than scaffolds, which may allow for better screening of NR-related drugs. Note that the 3D descriptors are not used in the current model construction of PbsNRs. In the future, with the accumulation of bioactive data and elucidated NR structure, it is possible to further improve the performance of PbsNRs by adding descriptors that could capture dynamic and structural properties.

**Key Points**

- PbsNRs introduce PCM modeling in nuclear receptor (NR)–related drug screening and demonstrate better prediction performance and application scope than current multitask quantitative structure–activity relationship–based state-of-the-art tools.
- PbsNRs incorporate tabular features such as sequence similarity and physical–chemical properties, which could be extended to NRs outside the training dataset and illustrate better performance on tree-based classifier than the deep learning model.
- The web server of PbsNRs is freely available at http://pbsnrs.badd-cao.net; the Python package of PbsNRs is implemented at GitHub (https://github.com/ddhmed/pisnrs), which could accelerate the detection of NR-related drug development.

## Supplementary Material

Supplementary_Files-clean_bbae710

Supplementary_Table_5_bbae710

Supplementary_Table_6_bbae710

## Data Availability

All data and scripts have been uploaded to https://github.com/ddhmed/pisnrs. The wrapper script in the commandline directory can be found in GitHub at: https://github.com/ddhmed/pisnrs/tree/master/commandline.
