# Supplementary material for: PbsNRs: predict the potential binders and scaffolds for nuclear receptors
Source: Brief Bioinform. 2025 Jan 11;26(1):bbae710. doi: 10.1093/bib/bbae710 (PMC11724720; doi:10.1093/bib/bbae710)
Supplement: Supplementary_Files-clean_bbae710 [file supplementary_files-clean_bbae710.docx]

**Supplementary Methods**

**Data collection**

Standard data records for model construction require structure information of chemical compounds and the sequences of protein targets, as well as bioactivity data between ligands and receptors. In the previous study [1], all the bioactivity data of chemical compounds, 11 nuclear receptors and the half maximal effective concentration (EC_50_) values of the compounds were collected from ONRLDB (version: v16) [2]. Among them, the bioactivity data for five nuclear receptors (NR1C1 (PPARα), NR1C2 (PPAR-β/δ), NR1C3 (PPARγ), NR1H2 (EcR) and NR2B1 (RARα)) were defined as the internal dataset. And the data for the other 6 NRs were defined as the external dataset. In this article, 35,941 chemical structures in .mol files were derived from TCM Database@Taiwan [3] (version 2014-01-31). Moreover, chemical compounds were also derived from ChEMBL [4] and Drugbank [5] in order to evaluate the amount of potential binders for NRs in those datasets.

**Model optimization**

For model optimization, approaches including Gaussian Naïve Bayens (GNB), Random Forest (RF), Ridge Classifier (RC), Logistic Regression (LR), and Support Vector Classification (SVC) are selected, which is implemented by the Python package of “Scikit-learn” with default parameters [58]. Detailed parameters for model optimization can be found in **Supplementary Table 5.** Moreover, the Deep Learning (DL) approach is implemented by the Python package of “fastai” (The version is 1.0.61). This model is comprised of a series of hidden modules and each module contains fixed layers: one ReLU layer, one BatchNorm1d layer, one Dropout layer and one linear layer. The parameters of DL including the number of modules, the dropout rate and the number of neurons in each layer, were set as 2, 0.5 and 200 respectively. The DL models were optimized through parameter screening of the number of hidden modules, the number of neurons in each module, and the dropout rate, listed in **Supplementary Table 6**. The internal data is split up into training and testing datasets randomly according to the ratio of 80% and 20%. Those models were evaluated based on the internal validation dataset derived from ONRLBD [51]. Among them, RF and DL outperform others, achieving ROC-AUC values of 0.886 and 0.899 on internal validation, followed by RC (0.726), GNB (0.547), SVC (0.547), and LR (0.475) (**Supplementary Figure 1**). Therefore, RF and DL were selected for further parameter optimization and model construction.

**MMPs generation and Molecular scaffold searching**

The discovery of bioactive scaffolds often leads to the discovery of a group of drugs. Thus, in order to develop new potential drugs for NRs, it is crucial to find the shared scaffolds of the known binders, which requires the methodology of matched molecular pairs (MMPs). MMP is a pair of compounds that share a similar scaffold and only differ by a single localized structural change. Based on the MMP methodology, a network can be generated and the scaffold of a group of binders can be identified.

The matched molecular pairs of natural products and the related network can be generated through the pipelines of Hussain J’s work through the Python package of RDKit [6]. The algorithm works by fragmenting and appropriately indexing the compounds under analysis. Here, the SMILES files of all the natural products were involved for MMPs generation. Further, for each protein target, the Bemis-Murcko scaffold for the cluster of bioactive compounds was derived for further analysis [1, 7].

**Model comparison and Evaluation parameters**

The threshold of bioactive and inactive compounds was defined by EC50 values, while compounds with EC50<1μM against target NRs were identified as potential bioactive binders for corresponding NRs, otherwise, identified as bio-inactive ones.

The nuclear receptor binder prediction can be regarded as the part of protein binder prediction. In tool comparison, we select the representative tools from two classes: Tsubaki’s model and DeepCPI for protein-ligand prediction and NR-Toxpred and NR-Profiler for nuclear receptors binder prediction. For NR-Toxpred and NR-Profiler. Since NR-Toxpred and NR- Profiler only apply to a limited number of NR families, the corresponding NRs from the external dataset were separately selected for validation. (Supplementary Table 8). For statistical analysis, we apply Mann–Whitney U test. In Tsubaki’s model, the data pre-processing and the feature extraction are based on the different sizes of input data to generate the feature vectors (<https://github.com/masashitsubaki/CPI_prediction>). For comparison, we generate the features vector of Tsubaki’s model based on their training dataset and our external dataset.

Moreover, the evaluation performance of different PCM modeling is evaluated based on the receiver operating characteristic (ROC) curve, which includes the area under the ROC curve (AUC), accuracy, precision, recall and F1-score [1]. The ROC curve is created by plotting the recall value against the false positive rate (FPR) at various threshold settings. The definition of accuracy, precision, FPR, recall, F1-score, and balanced accuracy was given as follows:

$Accuracy=\frac{TP+TN}{TP+FP+TN+FN}$ (1)

$Precision=\frac{\mathrm{TP}}{TP+FP}$ (2)

$FPR=\frac{FP}{FP+TN}$ (3)

$Recall=\frac{TP}{TP+FN}$ (4)

$F1-score=2\cdot\frac{precision \cdot recall}{precision+recall}$ (5)

$Balanced Accuracy=\frac{TP}{TP+FN}+\frac{TN}{TN+FP}$ (6)

Positive samples are those with EC_50_ value below threshold. TP represents True positive, TN represents true negative, FP represents false positive and FN represent false negative.

**Construction and validation of biosensors for NRs’ ligands detection**

To further evaluate the potential of drug screening, a biosensor was constructed for the validation of the predicted chemicals from PbsNRs. Initially, the NR2B1 protein (**Supplementary Material 1**) was synthesized by the Sangon Biotech based on the pre-specific sequences and was purified through the E. coli expression system. Above seven compounds were purchased from Shanghai Mackline Biochemical Co., Ltd. Then, a three-electrode system with Ag/AgCl as reference electrode, Pt electrode as counter electrode and biosensor as working electrode was used for the experimental validation. PBS at pH 7.0 was used as the working substrate for the cyclic voltammetry assay (CV). The potential was set from -0.2 to +0.6 V, and the scan rate was 100 mV/s. The whole detection procedure was performed at room temperature.

The fabrication process of this biosensor was investigated using CV and EIS (Electrochemical Impedance Spectroscopy) methods. As can be seen in **Supplementary Figure 1**, the CV curve has a clear separation of redox peaks between the peaks of [Fe(CN)6] 3-/4-. The CV response signal changes significantly due to the decrease in diffusion coefficient and the limitation of electron transfer. **Supplementary Figure 2** shows the EIS curves of Bi2O3@Au, Bi2O3@Au@NR2B1 and bare electrodes. The redox peaks of Bi2O3@Au and Bi2O3@Au@NR2B1 modified gold electrodes are significantly lower than those of bare gold electrodes. For cleaned bare electrodes, the resistance is low and when the nanomaterial Bi2O3@Au is modified onto the electrode surface, increasing the difficulty of charge transfer between the electrode surface and the electrolyte solution, the impedance values are greater than for bare electrodes. This is due to the increased thickness of the modified layer and the significant conformational changes that occur on the electrode surface. After the addition of protein, Bi2O3@Au binds specifically to the protein, forming a complex product that further blocks electron transfer and further increases the impedance value, in agreement with the CV curve results, all of which indicate the successful construction of the Bi2O3@Au@NR2B1 sensor.

**Development of web server**

After the assessment of the model, in order to make it available to search molecules and predict, a web server was developed based on the Python Django framework. The database was based on sqlite3 and the front-end language is HTML. We also used Bootstrap (https://getbootstrap.com/) for layout and styling, Highcharts [8] for statistics visualization and Ketcher [9] for molecule structure input.

**PbsNRs implementation**

The webserver of PbsNRs can be accessed at: <http://pbsnrs.badd-cao.net>, which is described as follows:

***Prediction:*** Currently, PbsNRs accepts three types of input files: 1) molecules in SMILES files; 2) molecules in .Mol files; and 3) custom-defined molecular structure through a server plug-in component. After uploading, PbsNRs will automatically calculate the 187-dimensional descriptors of input molecules and 30-dimensional protein target descriptors of all built-in NR targets. Then, the result page will pop up with information on 1) input molecules, 2) derived Murcko Scaffold including both image and SMILES files, and 3) predicted activity score of input molecules against built-in NR targets. By clicking the ID of each NR, the 3D structures of the target protein can be viewed. Meanwhile, the corresponding Protein Data Bank (PDB) ID [10] and Uniprot ID [11] were provided. Note that batch uploading with SMILES files is encouraged. Each time, maximally 100 molecules could be uploaded as queries. Then, the activity score and molecular scaffold will be calculated for each query automatically. The prediction results can be downloaded in .txt files. The time complexity of the prediction model is ~O(n), which requires 8.6 minutes on the prediction of 1,000 queries (30,000 tasks for all 30 NRs) on personal computer (CPU: Inter®Core™i7, [5700HQCPU@2.60GHz](mailto:5700HQCPU@2.60GHz), internal memory: 8GB). In addition, an application programming interface (API) was included in PbsNRs to provide a stable interface for batch predictions.

***Search and Browser:*** Also, users can search the ligand information in our built-in database. Currently, PbsNRs accept two kinds of searching patterns: 1) text search, according to the compound name, and 2) similarity search based on the structure similarity of molecules. The top 50 results of text and structure search with the entered chemical name and structure similarity will be provided respectively. It costs approximately 0.44s for text search and 1.89s for structure search respectively. For the browser, the information on NRs, NR-related ligands, the basic scaffold of NR-related ligands and experimental activities can be visualized for users. The structure of NRs was derived from PDB [10]. Ligands information and experimental activities were derived from ONRLDB [2], as well as Bemis-Murcko scaffolds for all ligands [7].

***Python implementation:*** The local version of PbsNRs is a Python package which is constructed based on RDKit and scikit-learn [12]. This package is also under the MIT license, which allows others to process the package without restriction. The installation of PbsNRs requires: Python (>= version 3.6), RDKit (>= version 2018.03.2.0), scikit-learn (>= 0.19.1) and pandas (>=0.23.1). Details of installation of PbsNRs package, along with examples and data were deposited at GitHub (see ***DATA AND CODE AVAILABILITY***). The local version can provide full functions of prediction for both individual and batch queries.

**Supplementary Figures**


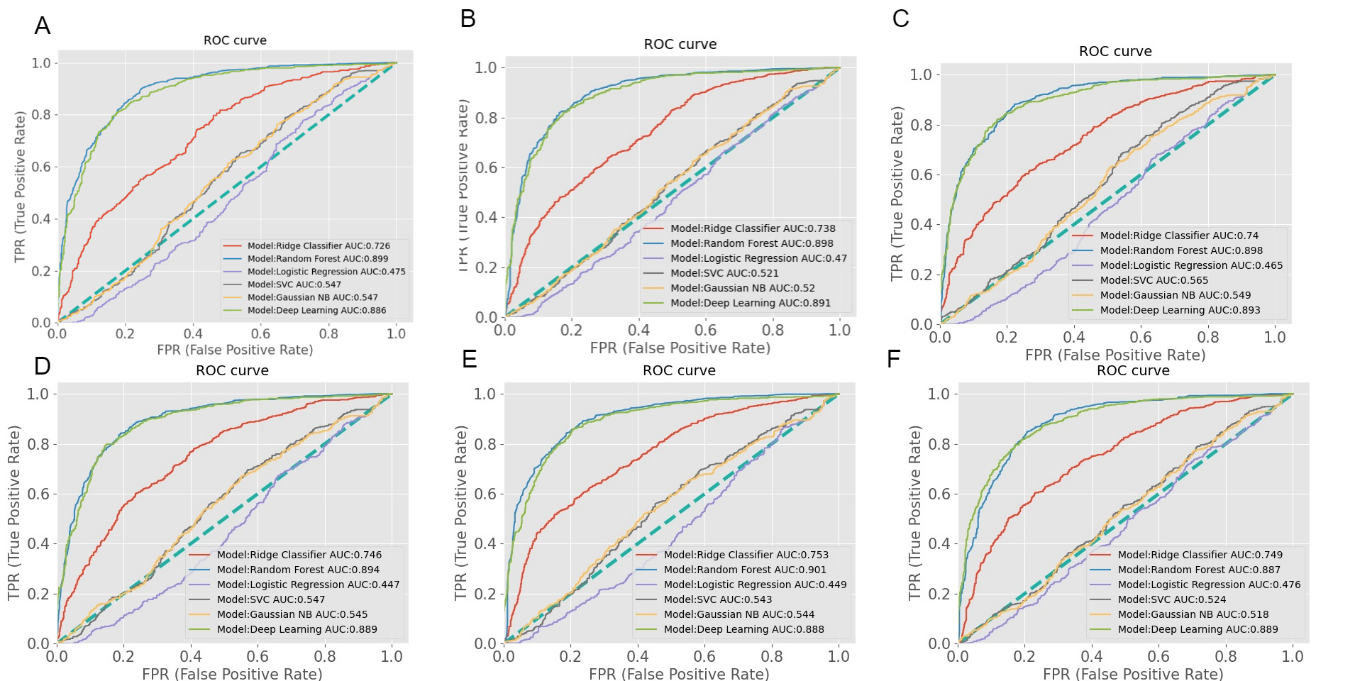


**Supplementary Figure 1.** The ROC curve of six models, Ridge Classifier, Random Forest, Logistic Regression, SVC (Support Vector Classifier), Gaussian NB and Deep Learning on internal dataset. The internal data is split up into train and test data randomly according to the ratio of 4 to 1. After the training, the test data is used to evaluate those models.


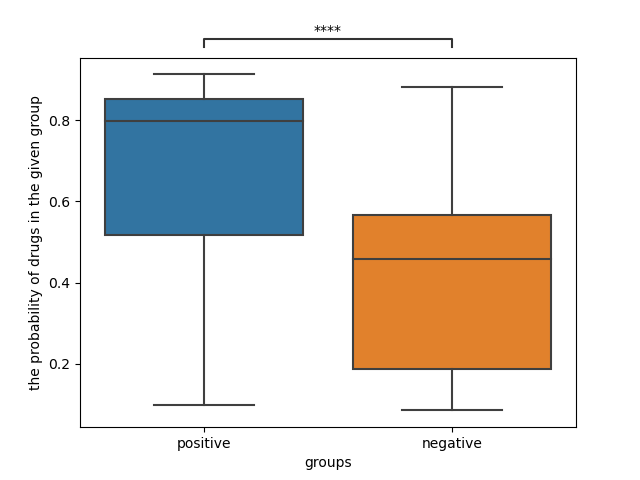


**Supplementary Figure 2.** The probability distribution of positive and negative compounds in external dataset predicted by PbsNRs.


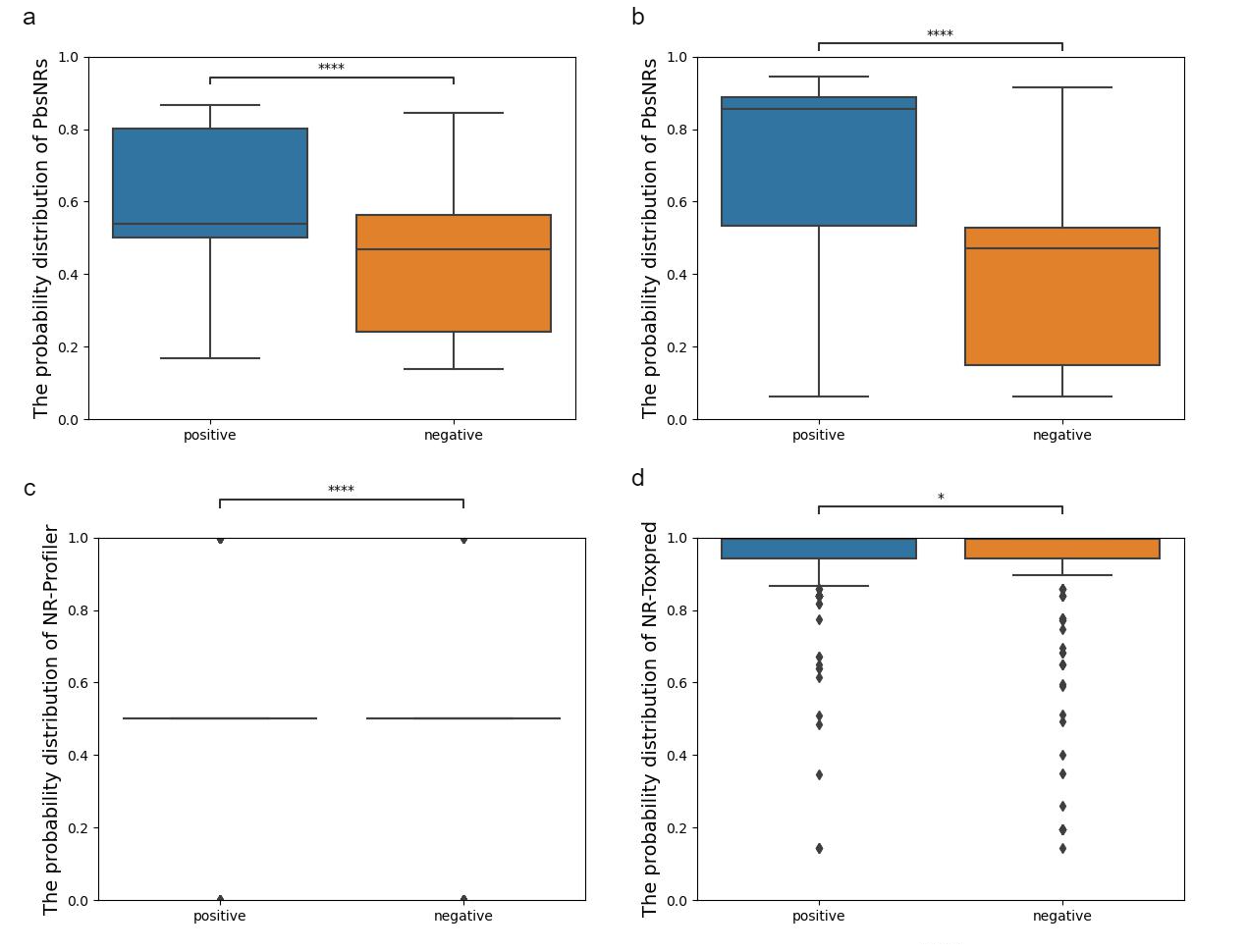


**Supplementary Figure 3.** Comparison of the probability distribution of positive and negative compounds in differents datasets predicted by PbsNRs, NR-Profiler and NR toxpred. (a) The statistical analysis of PbsNRs on NR1H4, NR2B2 and NR2B3 dataset. (b)The statistical analysis of PbsNRs on NR1H3, NR1H4 and NR1I2 dataset. (c)The statistical analysis of NR-Profiler on NR1H4, NR2B2 and NR2B3 dataset. (d) The statistical analysis of NR-toxpred on NR1H3, NR1H4 and NR1I2 dataset. The number of star symbols represents the P value. ‘*’ means that P value is less than 0.05. ‘****’ means that P value is less than 0.0001.


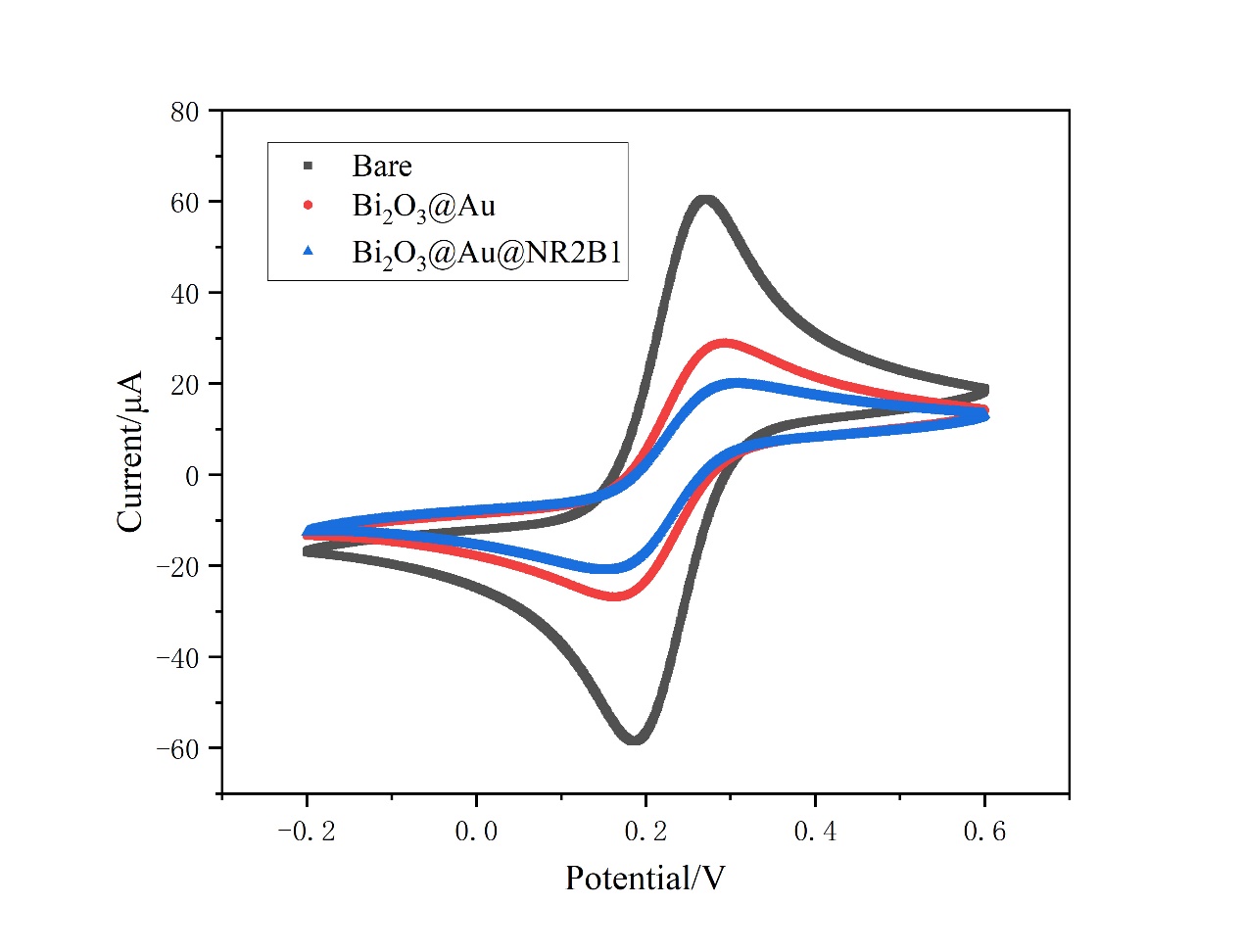


**Supplementary Figure 4.** The CV (current versus potential) curves of three electrodes, Bare, Bi2O3@Au and Bi2O3@Au@NR2B1.


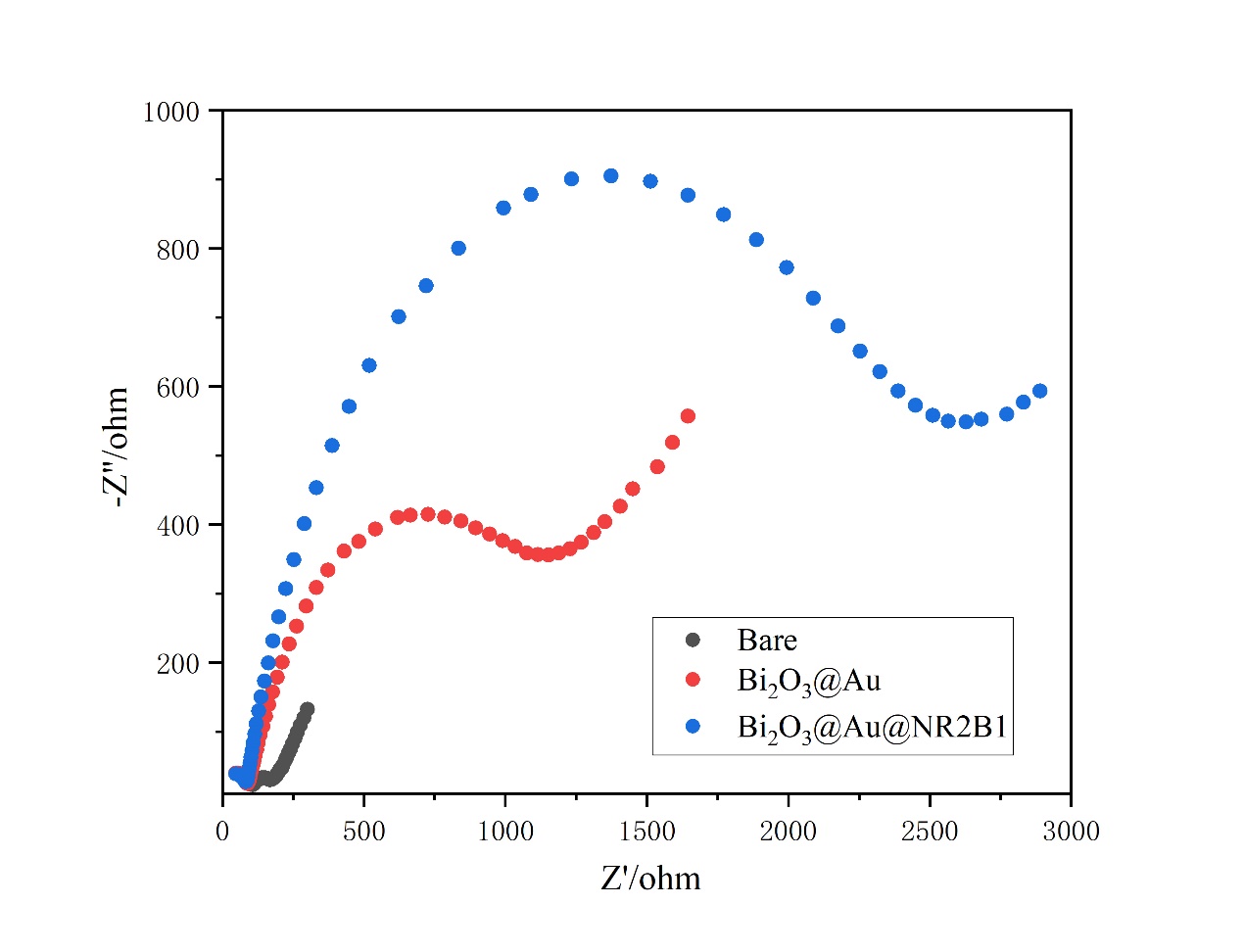


**Supplementary Figure 5.** The ELS (Electrochemical Impedance Spectroscopy) curves of three electrodes, Bare, Bi2O3@Au and Bi2O3@Au@NR2B1.


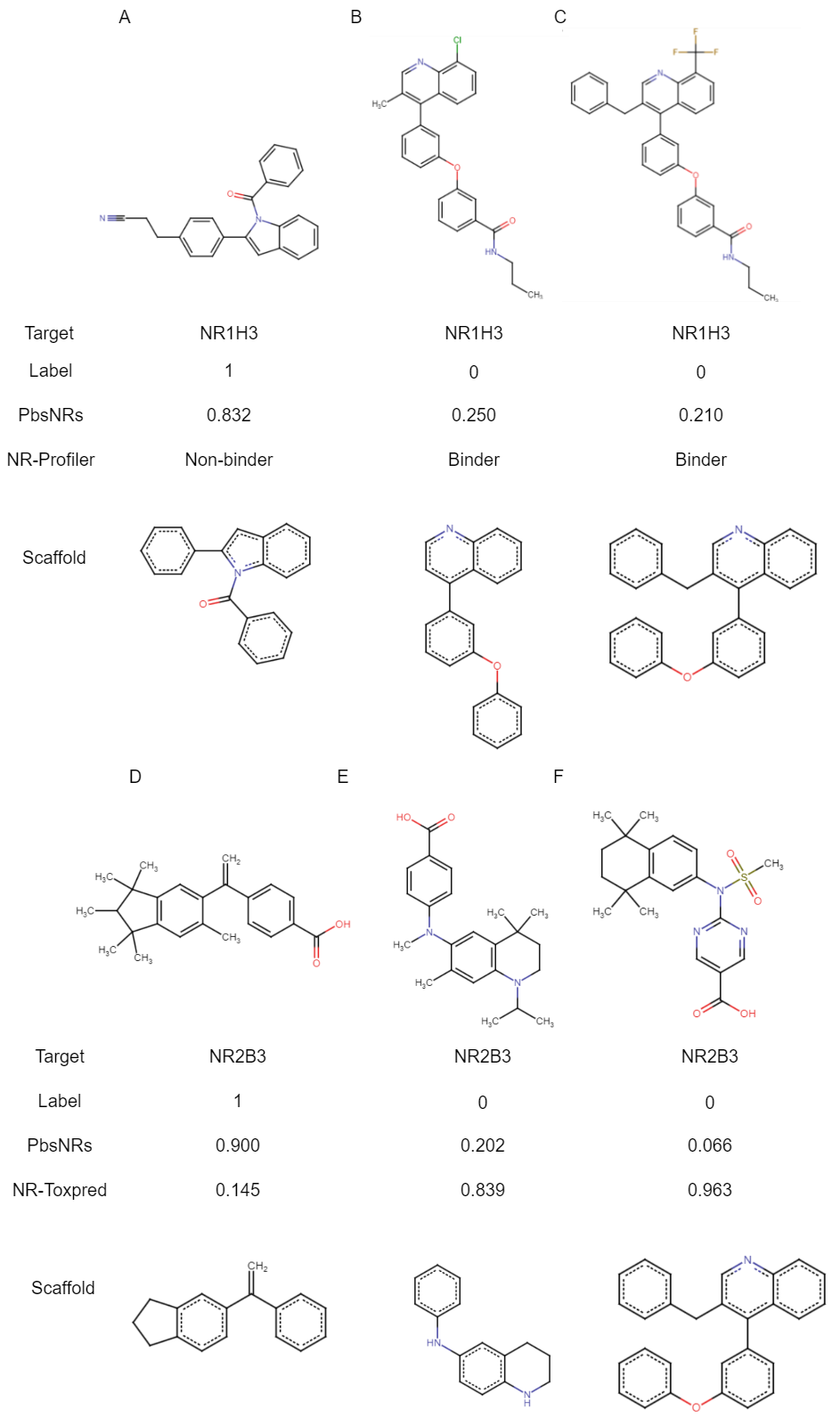


**Supplementary Figure 6. Case study between PbsNRs and other SOTA tools.** Structure, target, ground truth label, prediction output and scaffold of (a) 3-[4-(1-benzoylindol-2-yl)phenyl] propane nitrile, (b) 3-[3-(8-chloro-3-methylquinolin-4-yl)phenoxy]-N-propylbenzamide, (c) 3-{3-[3-benzyl-8-(trifluoromethyl)quinolin-4-yl]phenoxy}-N-propylbenzamide, (d) 4-[1-(1,1,2,3,3,6-hexamethyl-2H-inden-5-yl)ethenyl]benzoic acid, (e) 4-[(1-Isopropyl-4,4,7-trimethyl-1,2,3,4-tetrahydroquinolin-6-yl)methylamino]benZoic acid, (f) 2-(N-(5,5,8,8-tetramethyl-5,6,7,8-tetrahydronaphthalen-2-yl)methylsulfonamido)pyrimidine-5-carboxylic acid.

**Supplementary Tables**

**Supplementary Table 1.** Data distribution of different NR targets in ONRLDB.

| TargetID | Symbol | Number of compounds |
| --- | --- | --- |
| NR1C3 | PPAR_gamma | 2504 |
| NR1C1 | PPAR_alpha | 2275 |
| NR1C2 | PPAR_delta | 1120 |
| NR1H2 | LXR_beta | 382 |
| NR2B1 | RXR_alpha | 273 |
| NR1H3 | LXR_alpha | 205 |
| NR1H4 | FXR | 175 |
| NR2B3 | RXR_gamma | 156 |
| NR2B2 | RXR_beta | 142 |
| NR1D1 | Rev_erb_alpha | 23 |
| NR1I2 | PXR | 12 |

**Supplementary Table 2.** Information of chemical descriptors.

| NO | Name of descriptor | Category of descriptor | Reference |
| --- | --- | --- | --- |
| 1 | MaxEStateIndex | Basic EState definitions | Hall, Mohney and Kier. JCICS _31_ 76-81 (1991) |
| 2 | MinEStateIndex |  |  |
| 3 | MaxAbsEStateIndex |  |  |
| 4 | MinAbsEStateIndex |  |  |
| 5 | BalabanJ | Calculation of topological/topochemical descriptors. | Chem. Phys. Lett. vol 89, 399-404, (1982) |
| 6 | BertzCT | Calculation of topological/topochemical descriptors. | S. H. Bertz, J. Am. Chem. Soc., vol 103, 3599-3601 (1981) |
| 7 | Chi0 | Calculation of topological/topochemical descriptors. | Rev. Comput. Chem. 2:367-422 (1991) |
| 8 | Chi0n |  |  |
| 9 | Chi0v |  |  |
| 10 | Chi1 |  |  |
| 11 | Chi1n |  |  |
| 12 | Chi1v |  |  |
| 13 | Chi2n |  |  |
| 14 | Chi2v |  |  |
| 15 | Chi3n |  |  |
| 16 | Chi3v |  |  |
| 17 | Chi4n |  |  |
| 18 | Chi4v |  |  |
| 19 | EState_VSA1 | Hybrid EState-VSA descriptors (Estate VSA Descriptor 1-11) | http://www.rdkit.org/docs/api/rdkit.Chem.EState.EState_VSA-module.html |
| 20 | EState_VSA2 |  |  |
| 21 | EState_VSA3 |  |  |
| 22 | EState_VSA4 |  |  |
| 23 | EState_VSA5 |  |  |
| 24 | EState_VSA6 |  |  |
| 25 | EState_VSA7 |  |  |
| 26 | EState_VSA8 |  |  |
| 27 | EState_VSA9 |  |  |
| 28 | EState_VSA10 |  |  |
| 29 | EState_VSA11 |  |  |
| 30 | FractionCSP3 | Calculation of Lipinski parameters for molecules | http://www.rdkit.org/docs/api/rdkit.Chem.Lipinski-module.html#FractionCSP3 |
| 31 | HallKierAlpha | Calculation of topological/topochemical descriptors. | http://www.rdkit.org/docs/api/rdkit.Chem.GraphDescriptors-module.html#HallKierAlpha |
| 32 | HeavyAtomCount | Calculation of Lipinski parameters for molecules | http://www.rdkit.org/docs/api/rdkit.Chem.Lipinski-module.html#HeavyAtomCount |
| 33 | Ipc | Calculation of topological/topochemical descriptors. | D. Bonchev & N. Trinajstic, J. Chem. Phys. vol 67, 4517-4533 (1977 |
| 34 | Kappa1 | Calculation of topological/topochemical descriptors. | http://www.rdkit.org/docs/api/rdkit.Chem.GraphDescriptors-module.html |
| 35 | Kappa2 |  |  |
| 36 | Kappa3 |  |  |
| 37 | LabuteASA | Exposes functionality for MOE-like approximate molecular surface area descriptors | http://www.rdkit.org/docs/api/rdkit.Chem.MolSurf-module.html#LabuteASA |
| 38 | MolLogP | Atom-based calculation of LogP and MR using Crippen's approach | S. A. Wildman and G. M. Crippen JCICS 39 868-873 (1999) |
| 39 | MolMR |  |  |
| 40 | NHOHCount | Calculation of Lipinski parameters for molecules | http://www.rdkit.org/docs/api/rdkit.Chem.Lipinski-module.html |
| 41 | NOCount |  |  |
| 42 | NumAliphaticCarbocycles |  |  |
| 43 | NumAliphaticHeterocycles |  |  |
| 44 | NumAliphaticRings |  |  |
| 45 | NumAromaticCarbocycles |  |  |
| 46 | NumAromaticHeterocycles |  |  |
| 47 | NumAromaticRings |  |  |
| 48 | NumHAcceptors |  |  |
| 49 | NumHDonors |  |  |
| 50 | NumHeteroatoms |  |  |
| 51 | NumRotatableBonds |  |  |
| 52 | NumSaturatedCarbocycles |  |  |
| 53 | NumSaturatedHeterocycles |  |  |
| 54 | NumSaturatedRings |  |  |
| 55 | PEOE_VSA1 | Exposes functionality for MOE-like approximate molecular surface area descriptors (MOE Charge VSA Descriptor 1-14). | http://www.rdkit.org/docs/api/rdkit.Chem.MolSurf-module.html |
| 56 | PEOE_VSA2 |  |  |
| 57 | PEOE_VSA3 |  |  |
| 58 | PEOE_VSA4 |  |  |
| 59 | PEOE_VSA5 |  |  |
| 60 | PEOE_VSA6 |  |  |
| 61 | PEOE_VSA7 |  |  |
| 62 | PEOE_VSA8 |  |  |
| 63 | PEOE_VSA9 |  |  |
| 64 | PEOE_VSA10 |  |  |
| 65 | PEOE_VSA11 |  |  |
| 66 | PEOE_VSA12 |  |  |
| 67 | PEOE_VSA13 |  |  |
| 68 | PEOE_VSA14 |  |  |
| 69 | RingCount | Calculation of Lipinski parameters for molecules | http://www.rdkit.org/docs/api/rdkit.Chem.Lipinski-module.html#RingCount |
| 70 | SMR_VSA1 | Exposes functionality for MOE-like approximate molecular surface area descriptors (MOE MR VSA Descriptor 1-10). | http://www.rdkit.org/docs/api/rdkit.Chem.MolSurf-module.html |
| 71 | SMR_VSA2 |  |  |
| 72 | SMR_VSA3 |  |  |
| 73 | SMR_VSA4 |  |  |
| 74 | SMR_VSA5 |  |  |
| 75 | SMR_VSA6 |  |  |
| 76 | SMR_VSA7 |  |  |
| 77 | SMR_VSA8 |  |  |
| 78 | SMR_VSA9 |  |  |
| 79 | SMR_VSA10 |  |  |
| 80 | SlogP_VSA1 | Exposes functionality for MOE-like approximate molecular surface area descriptors (MOE logP VSA descriptor 1-12). | http://www.rdkit.org/docs/api/rdkit.Chem.MolSurf-module.html |
| 81 | SlogP_VSA2 |  |  |
| 82 | SlogP_VSA3 |  |  |
| 83 | SlogP_VSA4 |  |  |
| 84 | SlogP_VSA5 |  |  |
| 85 | SlogP_VSA6 |  |  |
| 86 | SlogP_VSA7 |  |  |
| 87 | SlogP_VSA8 |  |  |
| 88 | SlogP_VSA9 |  |  |
| 89 | SlogP_VSA10 |  |  |
| 90 | SlogP_VSA11 |  |  |
| 91 | SlogP_VSA12 |  |  |
| 92 | TPSA | Exposes functionality for MOE-like approximate molecular surface area descriptors. | http://www.rdkit.org/docs/api/rdkit.Chem.MolSurf-module.html#TPSA |
| 93 | VSA_EState1 | Hybrid EState-VSA descriptors (VSA Estate Descriptor 1-10) | http://www.rdkit.org/docs/api/rdkit.Chem.EState.EState_VSA-module.html |
| 94 | VSA_EState2 |  |  |
| 95 | VSA_EState3 |  |  |
| 96 | VSA_EState4 |  |  |
| 97 | VSA_EState5 |  |  |
| 98 | VSA_EState6 |  |  |
| 99 | VSA_EState7 |  |  |
| 100 | VSA_EState8 |  |  |
| 101 | VSA_EState9 |  |  |
| 102 | VSA_EState10 |  |  |
| 103 | fr_Al_COO | functions to match a bunch of fragment descriptors from a file | http://www.rdkit.org/docs/api/rdkit.Chem.Fragments-module.html#fr_Al_COO |
| 104 | fr_Al_OH | functions to match a bunch of fragment descriptors from a file | http://www.rdkit.org/docs/api/rdkit.Chem.Fragments-module.html#fr_Al_OH |
| 105 | fr_Al_OH_noTert | functions to match a bunch of fragment descriptors from a file | http://www.rdkit.org/docs/api/rdkit.Chem.Fragments-module.html#fr_Al_OH_noTert |
| 106 | fr_ArN | functions to match a bunch of fragment descriptors from a file | http://www.rdkit.org/docs/api/rdkit.Chem.Fragments-module.html#fr_ArN |
| 107 | fr_Ar_COO | functions to match a bunch of fragment descriptors from a file | http://www.rdkit.org/docs/api/rdkit.Chem.Fragments-module.html#fr_Ar_COO |
| 108 | fr_Ar_N | functions to match a bunch of fragment descriptors from a file | http://www.rdkit.org/docs/api/rdkit.Chem.Fragments-module.html#fr_Ar_N |
| 109 | fr_Ar_NH | functions to match a bunch of fragment descriptors from a file | http://www.rdkit.org/docs/api/rdkit.Chem.Fragments-module.html#fr_Ar_NH |
| 110 | fr_Ar_OH | functions to match a bunch of fragment descriptors from a file | http://www.rdkit.org/docs/api/rdkit.Chem.Fragments-module.html#fr_Ar_OH |
| 111 | fr_COO | functions to match a bunch of fragment descriptors from a file | http://www.rdkit.org/docs/api/rdkit.Chem.Fragments-module.html#fr_COO |
| 112 | fr_COO2 | functions to match a bunch of fragment descriptors from a file | http://www.rdkit.org/docs/api/rdkit.Chem.Fragments-module.html#fr_COO2 |
| 113 | fr_C_O | functions to match a bunch of fragment descriptors from a file | http://www.rdkit.org/docs/api/rdkit.Chem.Fragments-module.html#fr_C_O |
| 114 | fr_C_O_noCOO | functions to match a bunch of fragment descriptors from a file | http://www.rdkit.org/docs/api/rdkit.Chem.Fragments-module.html#fr_C_O_noCOO |
| 115 | fr_C_S | functions to match a bunch of fragment descriptors from a file | http://www.rdkit.org/docs/api/rdkit.Chem.Fragments-module.html#fr_C_S |
| 116 | fr_HOCCN | functions to match a bunch of fragment descriptors from a file | http://www.rdkit.org/docs/api/rdkit.Chem.Fragments-module.html#fr_HOCCN |
| 117 | fr_Imine | functions to match a bunch of fragment descriptors from a file | http://www.rdkit.org/docs/api/rdkit.Chem.Fragments-module.html#fr_Imine |
| 118 | fr_NH0 | functions to match a bunch of fragment descriptors from a file | http://www.rdkit.org/docs/api/rdkit.Chem.Fragments-module.html#fr_NH0 |
| 119 | fr_NH1 | functions to match a bunch of fragment descriptors from a file | http://www.rdkit.org/docs/api/rdkit.Chem.Fragments-module.html#fr_NH1 |
| 120 | fr_NH2 | functions to match a bunch of fragment descriptors from a file | http://www.rdkit.org/docs/api/rdkit.Chem.Fragments-module.html#fr_NH2 |
| 121 | fr_N_O | functions to match a bunch of fragment descriptors from a file | http://www.rdkit.org/docs/api/rdkit.Chem.Fragments-module.html#fr_N_O |
| 122 | fr_Ndealkylation1 | functions to match a bunch of fragment descriptors from a file | http://www.rdkit.org/docs/api/rdkit.Chem.Fragments-module.html#fr_Ndealkylation1 |
| 123 | fr_Ndealkylation2 | functions to match a bunch of fragment descriptors from a file | http://www.rdkit.org/docs/api/rdkit.Chem.Fragments-module.html#fr_Ndealkylation2 |
| 124 | fr_Nhpyrrole | functions to match a bunch of fragment descriptors from a file | http://www.rdkit.org/docs/api/rdkit.Chem.Fragments-module.html#fr_Nhpyrrole |
| 125 | fr_SH | functions to match a bunch of fragment descriptors from a file | http://www.rdkit.org/docs/api/rdkit.Chem.Fragments-module.html#fr_SH |
| 126 | fr_aldehyde | functions to match a bunch of fragment descriptors from a file | http://www.rdkit.org/docs/api/rdkit.Chem.Fragments-module.html#fr_aldehyde |
| 127 | fr_alkyl_carbamate | functions to match a bunch of fragment descriptors from a file | http://www.rdkit.org/docs/api/rdkit.Chem.Fragments-module.html#fr_alkyl_carbamate |
| 128 | fr_alkyl_halide | functions to match a bunch of fragment descriptors from a file | http://www.rdkit.org/docs/api/rdkit.Chem.Fragments-module.html#fr_alkyl_halide |
| 129 | fr_allylic_oxid | functions to match a bunch of fragment descriptors from a file | http://www.rdkit.org/docs/api/rdkit.Chem.Fragments-module.html#fr_allylic_oxid |
| 130 | fr_amide | functions to match a bunch of fragment descriptors from a file | http://www.rdkit.org/docs/api/rdkit.Chem.Fragments-module.html#fr_amide |
| 131 | fr_amidine | functions to match a bunch of fragment descriptors from a file | http://www.rdkit.org/docs/api/rdkit.Chem.Fragments-module.html#fr_amidine |
| 132 | fr_aniline | functions to match a bunch of fragment descriptors from a file | http://www.rdkit.org/docs/api/rdkit.Chem.Fragments-module.html#fr_aniline |
| 133 | fr_aryl_methyl | functions to match a bunch of fragment descriptors from a file | http://www.rdkit.org/docs/api/rdkit.Chem.Fragments-module.html#fr_aryl_methyl |
| 134 | fr_azide | functions to match a bunch of fragment descriptors from a file | http://www.rdkit.org/docs/api/rdkit.Chem.Fragments-module.html#fr_azide |
| 135 | fr_azo | functions to match a bunch of fragment descriptors from a file | http://www.rdkit.org/docs/api/rdkit.Chem.Fragments-module.html#fr_azo |
| 136 | fr_barbitur | functions to match a bunch of fragment descriptors from a file | http://www.rdkit.org/docs/api/rdkit.Chem.Fragments-module.html#fr_barbitur |
| 137 | fr_benzene | functions to match a bunch of fragment descriptors from a file | http://www.rdkit.org/docs/api/rdkit.Chem.Fragments-module.html#fr_benzene |
| 138 | fr_benzodiazepine | functions to match a bunch of fragment descriptors from a file | http://www.rdkit.org/docs/api/rdkit.Chem.Fragments-module.html#fr_benzodiazepine |
| 139 | fr_bicyclic | functions to match a bunch of fragment descriptors from a file | http://www.rdkit.org/docs/api/rdkit.Chem.Fragments-module.html#fr_bicyclic |
| 140 | fr_diazo | functions to match a bunch of fragment descriptors from a file | http://www.rdkit.org/docs/api/rdkit.Chem.Fragments-module.html#fr_diazo |
| 141 | fr_dihydropyridine | functions to match a bunch of fragment descriptors from a file | http://www.rdkit.org/docs/api/rdkit.Chem.Fragments-module.html#fr_dihydropyridine |
| 142 | fr_epoxide | functions to match a bunch of fragment descriptors from a file | http://www.rdkit.org/docs/api/rdkit.Chem.Fragments-module.html#fr_epoxide |
| 143 | fr_ester | functions to match a bunch of fragment descriptors from a file | http://www.rdkit.org/docs/api/rdkit.Chem.Fragments-module.html#fr_ester |
| 144 | fr_ether | functions to match a bunch of fragment descriptors from a file | http://www.rdkit.org/docs/api/rdkit.Chem.Fragments-module.html#fr_ether |
| 145 | fr_furan | functions to match a bunch of fragment descriptors from a file | http://www.rdkit.org/docs/api/rdkit.Chem.Fragments-module.html#fr_furan |
| 146 | fr_guanido | functions to match a bunch of fragment descriptors from a file | http://www.rdkit.org/docs/api/rdkit.Chem.Fragments-module.html#fr_guanido |
| 147 | fr_halogen | functions to match a bunch of fragment descriptors from a file | http://www.rdkit.org/docs/api/rdkit.Chem.Fragments-module.html#fr_halogen |
| 148 | fr_hdrzine | functions to match a bunch of fragment descriptors from a file | http://www.rdkit.org/docs/api/rdkit.Chem.Fragments-module.html#fr_hdrzine |
| 149 | fr_hdrzone | functions to match a bunch of fragment descriptors from a file | http://www.rdkit.org/docs/api/rdkit.Chem.Fragments-module.html#fr_hdrzone |
| 150 | fr_imidazole | functions to match a bunch of fragment descriptors from a file | http://www.rdkit.org/docs/api/rdkit.Chem.Fragments-module.html#fr_imidazole |
| 151 | fr_imide | functions to match a bunch of fragment descriptors from a file | http://www.rdkit.org/docs/api/rdkit.Chem.Fragments-module.html#fr_imide |
| 152 | fr_isocyan | functions to match a bunch of fragment descriptors from a file | http://www.rdkit.org/docs/api/rdkit.Chem.Fragments-module.html#fr_isocyan |
| 153 | fr_isothiocyan | functions to match a bunch of fragment descriptors from a file | http://www.rdkit.org/docs/api/rdkit.Chem.Fragments-module.html#fr_isothiocyan |
| 154 | fr_ketone | functions to match a bunch of fragment descriptors from a file | http://www.rdkit.org/docs/api/rdkit.Chem.Fragments-module.html#fr_ketone |
| 155 | fr_ketone_Topliss | functions to match a bunch of fragment descriptors from a file | http://www.rdkit.org/docs/api/rdkit.Chem.Fragments-module.html#fr_ketone_Topliss |
| 156 | fr_lactam | functions to match a bunch of fragment descriptors from a file | http://www.rdkit.org/docs/api/rdkit.Chem.Fragments-module.html#fr_lactam |
| 157 | fr_lactone | functions to match a bunch of fragment descriptors from a file | http://www.rdkit.org/docs/api/rdkit.Chem.Fragments-module.html#fr_lactone |
| 158 | fr_methoxy | functions to match a bunch of fragment descriptors from a file | http://www.rdkit.org/docs/api/rdkit.Chem.Fragments-module.html#fr_methoxy |
| 159 | fr_morpholine | functions to match a bunch of fragment descriptors from a file | http://www.rdkit.org/docs/api/rdkit.Chem.Fragments-module.html#fr_morpholine |
| 160 | fr_nitrile | functions to match a bunch of fragment descriptors from a file | http://www.rdkit.org/docs/api/rdkit.Chem.Fragments-module.html#fr_nitrile |
| 161 | fr_nitro | functions to match a bunch of fragment descriptors from a file | http://www.rdkit.org/docs/api/rdkit.Chem.Fragments-module.html#fr_nitro |
| 162 | fr_nitro_arom | functions to match a bunch of fragment descriptors from a file | http://www.rdkit.org/docs/api/rdkit.Chem.Fragments-module.html#fr_nitro_arom |
| 163 | fr_nitro_arom_nonortho | functions to match a bunch of fragment descriptors from a file | http://www.rdkit.org/docs/api/rdkit.Chem.Fragments-module.html#fr_nitro_arom_nonortho |
| 164 | fr_nitroso | functions to match a bunch of fragment descriptors from a file | http://www.rdkit.org/docs/api/rdkit.Chem.Fragments-module.html#fr_nitroso |
| 165 | fr_oxazole | functions to match a bunch of fragment descriptors from a file | http://www.rdkit.org/docs/api/rdkit.Chem.Fragments-module.html#fr_oxazole |
| 166 | fr_oxime | functions to match a bunch of fragment descriptors from a file | http://www.rdkit.org/docs/api/rdkit.Chem.Fragments-module.html#fr_oxime |
| 167 | fr_para_hydroxylation | functions to match a bunch of fragment descriptors from a file | http://www.rdkit.org/docs/api/rdkit.Chem.Fragments-module.html#fr_para_hydroxylation |
| 168 | fr_phenol | functions to match a bunch of fragment descriptors from a file | http://www.rdkit.org/docs/api/rdkit.Chem.Fragments-module.html#fr_phenol |
| 169 | fr_phenol_noOrthoHbond | functions to match a bunch of fragment descriptors from a file | http://www.rdkit.org/docs/api/rdkit.Chem.Fragments-module.html#fr_phenol_noOrthoHbond |
| 170 | fr_phos_acid | functions to match a bunch of fragment descriptors from a file | http://www.rdkit.org/docs/api/rdkit.Chem.Fragments-module.html#fr_phos_acid |
| 171 | fr_phos_ester | functions to match a bunch of fragment descriptors from a file | http://www.rdkit.org/docs/api/rdkit.Chem.Fragments-module.html#fr_phos_ester |
| 172 | fr_piperdine | functions to match a bunch of fragment descriptors from a file | http://www.rdkit.org/docs/api/rdkit.Chem.Fragments-module.html#fr_piperdine |
| 173 | fr_piperzine | functions to match a bunch of fragment descriptors from a file | http://www.rdkit.org/docs/api/rdkit.Chem.Fragments-module.html#fr_piperzine |
| 174 | fr_priamide | functions to match a bunch of fragment descriptors from a file | http://www.rdkit.org/docs/api/rdkit.Chem.Fragments-module.html#fr_priamide |
| 175 | fr_prisulfonamd | functions to match a bunch of fragment descriptors from a file | http://www.rdkit.org/docs/api/rdkit.Chem.Fragments-module.html#fr_prisulfonamd |
| 176 | fr_pyridine | functions to match a bunch of fragment descriptors from a file | http://www.rdkit.org/docs/api/rdkit.Chem.Fragments-module.html#fr_pyridine |
| 177 | fr_quatN | functions to match a bunch of fragment descriptors from a file | http://www.rdkit.org/docs/api/rdkit.Chem.Fragments-module.html#fr_quatN |
| 178 | fr_sulfide | functions to match a bunch of fragment descriptors from a file | http://www.rdkit.org/docs/api/rdkit.Chem.Fragments-module.html#fr_sulfide |
| 179 | fr_sulfonamd | functions to match a bunch of fragment descriptors from a file | http://www.rdkit.org/docs/api/rdkit.Chem.Fragments-module.html#fr_sulfonamd |
| 180 | fr_sulfone | functions to match a bunch of fragment descriptors from a file | http://www.rdkit.org/docs/api/rdkit.Chem.Fragments-module.html#fr_sulfone |
| 181 | fr_term_acetylene | functions to match a bunch of fragment descriptors from a file | http://www.rdkit.org/docs/api/rdkit.Chem.Fragments-module.html#fr_term_acetylene |
| 182 | fr_tetrazole | functions to match a bunch of fragment descriptors from a file | http://www.rdkit.org/docs/api/rdkit.Chem.Fragments-module.html#fr_tetrazole |
| 183 | fr_thiazole | functions to match a bunch of fragment descriptors from a file | http://www.rdkit.org/docs/api/rdkit.Chem.Fragments-module.html#fr_thiazole |
| 184 | fr_thiocyan | functions to match a bunch of fragment descriptors from a file | http://www.rdkit.org/docs/api/rdkit.Chem.Fragments-module.html#fr_thiocyan |
| 185 | fr_thiophene | functions to match a bunch of fragment descriptors from a file | http://www.rdkit.org/docs/api/rdkit.Chem.Fragments-module.html#fr_thiophene |
| 186 | fr_unbrch_alkane | functions to match a bunch of fragment descriptors from a file | http://www.rdkit.org/docs/api/rdkit.Chem.Fragments-module.html#fr_unbrch_alkane |
| 187 | fr_urea | functions to match a bunch of fragment descriptors from a file | http://www.rdkit.org/docs/api/rdkit.Chem.Fragments-module.html#fr_urea |

**Supplementary Table 3.** Information of crystal structure used for descriptor generation.

| PDB ID | Chain | Symbol | Exp. Method | Resolution | Structure MW | Residue Count |
| --- | --- | --- | --- | --- | --- | --- |
| 2Q3Y | B | NR0B2 | X-RAY DIFFRACTION | 2.4 | 30406.42 | 259 |
| 3UVV | A | NR1A1 | X-RAY DIFFRACTION | 2.95 | 58389.32 | 509 |
| 1DSZ | A | NR1B1 | X-RAY DIFFRACTION | 1.7 | 29654.19 | 201 |
| 3VI8 | A | NR1C1 | X-RAY DIFFRACTION | 1.75 | 31321.85 | 273 |
| 3TKM | A | NR1C2 | X-RAY DIFFRACTION | 1.95 | 31994.38 | 275 |
| 3U9Q | A | NR1C3 | X-RAY DIFFRACTION | 1.52 | 31877.6 | 278 |
| 1A6Y | A, B | NR1D1 | X-RAY DIFFRACTION | 2.3 | 34445.65 | 228 |
| 3CQV | A | NR1D2 | X-RAY DIFFRACTION | 1.9 | 23486.7 | 199 |
| 1N83 | A | NR1F1 | X-RAY DIFFRACTION | 1.63 | 31901.16 | 270 |
| 3L0L | A, B | NR1F3 | X-RAY DIFFRACTION | 1.74 | 61780.36 | 522 |
| 1UPV | A | NR1H2 | X-RAY DIFFRACTION | 2.1 | 29898.13 | 257 |
| 3IPQ | A | NR1H3 | X-RAY DIFFRACTION | 2 | 36351 | 308 |
| 1OSH | A | NR1H4 | X-RAY DIFFRACTION | 1.8 | 27583.96 | 232 |
| 1YNW | A | NR1I1 | X-RAY DIFFRACTION | 3 | 35710.27 | 245 |
| 3CTB | A, B | NR1I2 | X-RAY DIFFRACTION | 2 | 78368.8 | 688 |
| 3CBB | A, B | NR2A1 | X-RAY DIFFRACTION | 2 | 31260.2 | 198 |
| 2P1T | A | NR2B1 | X-RAY DIFFRACTION | 1.8 | 28816.56 | 253 |
| 1H9U | A, B, C, D | NR2B2 | X-RAY DIFFRACTION | 2.7 | 101130.9 | 896 |
| 2GL8 | A, B, C, D | NR2B3 | X-RAY DIFFRACTION | 2.4 | 107984.8 | 964 |
| 3P0U | A, B | NR2C2 | X-RAY DIFFRACTION | 3 | 56204.8 | 498 |
| 2EBL | A | NR2F1 | SOLUTION NMR | | 10031.11 | 89 |
| 3CJW | A | NR2F2 | X-RAY DIFFRACTION | 1.48 | 27311.7 | 244 |
| 3K6P | A | NR3B1 | X-RAY DIFFRACTION | 2 | 27586.98 | 248 |
| 1LO1 | A | NR3B2 | SOLUTION NMR | | 19223.21 | 124 |
| 2E2R | A | NR3B3 | X-RAY DIFFRACTION | 1.6 | 28115.87 | 244 |
| 2Z4J | A | NR3C4 | X-RAY DIFFRACTION | 2.6 | 30364.02 | 258 |
| 3V3E | A, B | NR4A1 | X-RAY DIFFRACTION | 2.06 | 57767.28 | 514 |
| 1OVL | A, D | NR4A2 | X-RAY DIFFRACTION | 2.2 | 186167.3 | 1626 |
| 1ZDT | A, B | NR5A1 | X-RAY DIFFRACTION | 2.1 | 59604.09 | 506 |
| 3PLZ | A, B | NR5A2 | X-RAY DIFFRACTION | 1.75 | 64028.82 | 542 |

**Supplementary Table 4.** Information of protein descriptors.

| NRs | NR0B2 | NR1A1 | NR1B1 | NR1C1 | NR1C2 | NR1C3 | NR1D1 | NR1D2 | NR1F1 | NR1F3 |
| --- | --- | --- | --- | --- | --- | --- | --- | --- | --- | --- |
| NR1C1 | 2.56 | 34.8 | 4.76 | 100 | 85.71 | 79.49 | 4.4 | 35.9 | 39.19 | 36.26 |
| NR1C2 | 2.55 | 37.82 | 4.73 | 85.09 | 100 | 79.27 | 4.36 | 38.91 | 47.27 | 39.64 |
| NR1C3 | 2.6 | 34.94 | 4.09 | 80.67 | 81.04 | 100 | 4.83 | 38.29 | 42.01 | 42.38 |
| NR1D1 | 6.38 | 11.7 | 65.96 | 15.96 | 12.77 | 13.83 | 100 | 7.45 | 14.89 | 12.77 |
| NR1H2 | 1.56 | 46.69 | 4.67 | 39.69 | 36.19 | 41.63 | 3.89 | 40.08 | 50.97 | 42.02 |
| NR1H3 | 2.12 | 49.47 | 3.53 | 43.82 | 38.16 | 40.64 | 2.83 | 43.11 | 51.24 | 47 |
| NR1H4 | 2.59 | 50.86 | 4.74 | 38.36 | 38.79 | 40.95 | 1.72 | 48.71 | 51.72 | 40.95 |
| NR1I2 | 1.45 | 34.01 | 3.2 | 25.58 | 29.65 | 30.81 | 1.45 | 30.81 | 36.92 | 31.69 |
| NR2B1 | 2.08 | 38.33 | 1.67 | 40.83 | 40 | 37.92 | 2.08 | 35.42 | 32.92 | 33.33 |
| NR2B2 | 2.23 | 38.84 | 1.79 | 40.63 | 41.52 | 39.29 | 2.68 | 38.84 | 35.27 | 32.59 |
| NR2B3 | 2.07 | 47.3 | 2.49 | 45.23 | 36.93 | 37.34 | 2.07 | 35.27 | 35.68 | 31.54 |
| NRs | **NR1H2** | **NR1H3** | **NR1H4** | **NR1I1** | **NR1I2** | **NR2A1** | **NR2B1** | **NR2B2** | **NR2B3** | **NR2C2** |
| NR1C1 | 37 | 46.15 | 32.6 | 2.2 | 32.6 | 5.49 | 35.9 | 33.33 | 39.93 | 32.6 |
| NR1C2 | 33.82 | 38.91 | 32.73 | 4 | 37.09 | 1.82 | 34.55 | 33.45 | 32.36 | 31.27 |
| NR1C3 | 40.15 | 42.75 | 35.32 | 5.58 | 39.03 | 3.35 | 33.83 | 32.71 | 33.46 | 31.23 |
| NR1D1 | 10.64 | 8.51 | 4.26 | 57.45 | 26.6 | 55.32 | 5.32 | 6.38 | 5.32 | 26.6 |
| NR1H2 | 100 | 83.66 | 43.97 | 2.72 | 56.03 | 6.23 | 46.69 | 40.47 | 41.63 | 31.52 |
| NR1H3 | 75.97 | 100 | 40.64 | 5.3 | 54.77 | 2.83 | 31.1 | 27.92 | 28.27 | 28.62 |
| NR1H4 | 48.71 | 49.14 | 100 | 6.9 | 47.84 | 2.59 | 38.36 | 38.36 | 44.4 | 34.05 |
| NR1I2 | 41.57 | 45.06 | 31.98 | 5.52 | 100 | 2.91 | 25.87 | 22.97 | 27.03 | 19.48 |
| NR2B1 | 50.42 | 36.67 | 37.08 | 7.5 | 36.25 | 3.33 | 100 | 87.08 | 94.17 | 49.17 |
| NR2B2 | 46.43 | 35.27 | 39.73 | 8.04 | 35.27 | 4.02 | 93.3 | 100 | 90.63 | 45.54 |
| NR2B3 | 44.4 | 33.2 | 43.15 | 7.47 | 39 | 3.73 | 93.78 | 84.23 | 100 | 43.57 |
| NRs | **NR2F1** | **NR2F2** | **NR3B1** | **NR3B2** | **NR3B3** | **NR3C4** | **NR4A1** | **NR4A2** | **NR5A1** | **NR5A2** |
| NR1C1 | 7.69 | 36.26 | 31.5 | 2.56 | 30.4 | 13.55 | 35.9 | 30.04 | 25.64 | 37.73 |
| NR1C2 | 2.18 | 33.45 | 36.73 | 7.64 | 30.18 | 32.36 | 39.64 | 36.36 | 26.18 | 30.18 |
| NR1C3 | 2.23 | 33.46 | 33.09 | 2.97 | 31.97 | 33.09 | 40.89 | 37.17 | 38.66 | 38.66 |
| NR1D1 | 58.51 | 35.11 | 10.64 | 61.7 | 5.32 | 11.7 | 8.51 | 7.45 | 8.51 | 23.4 |
| NR1H2 | 2.33 | 38.52 | 38.91 | 2.72 | 40.86 | 36.96 | 45.14 | 43.58 | 38.52 | 42.8 |
| NR1H3 | 2.83 | 39.22 | 35.34 | 3.18 | 41.34 | 32.51 | 44.88 | 44.52 | 35.34 | 41.7 |
| NR1H4 | 3.02 | 37.5 | 31.47 | 4.31 | 43.1 | 43.1 | 40.09 | 35.34 | 40.52 | 44.83 |
| NR1I2 | 2.62 | 31.4 | 22.67 | 3.2 | 28.49 | 26.45 | 29.65 | 34.59 | 26.45 | 35.76 |
| NR2B1 | 3.33 | 55.83 | 52.08 | 2.92 | 53.33 | 42.08 | 38.33 | 38.33 | 54.58 | 56.67 |
| NR2B2 | 4.91 | 56.25 | 52.68 | 3.13 | 52.68 | 43.75 | 37.05 | 37.05 | 47.77 | 52.23 |
| NR2B3 | 2.9 | 59.75 | 49.38 | 4.15 | 51.45 | 42.32 | 38.59 | 37.76 | 45.64 | 53.94 |

**Supplementary Table 5.** The performance of Random Forest under different parameters.

The first column represents the number of rows. The second column represents the number of fold that is used as test dataset. The third, fourth and firth columns represent the parameters of Random Forest, the number of random forest trees, the feature ratio of each tree, and the depth of each tree. The sixth, seventh, eighth and nineth columns represents the AUC value in different datasets.

**Supplementary Table 6.** The performance of Deep Learning under different parameters.

The first column represents the number of rows. The second column represents the number of fold that is used as test dataset. The third, fourth and firth columns represent the parameters of Deep Learning, the number of hidden layers, the number of neurons in one layer and dropout rate. The sixth and seventh columns represents the AUC value in different datasets.

**Supplementary Table 7.** The detailed architecture of Deep Learning approach.

| Layers: |  |  |
| --- | --- | --- |
| (0): Linear(in_features=217, out_features=300, bias=True) | | |
| (1): ReLU(inplace=True) | | |
| (2): BatchNorm1d(300, eps=1e-05, momentum=0.1, affine=True, track_running_stats=True) | | |
| (3): Dropout(p=0.5, inplace=False) | | |
| (4): Linear(in_features=300, out_features=300, bias=True) | | |
| (5): ReLU(inplace=True) | | |
| (6): BatchNorm1d(300, eps=1e-05, momentum=0.1, affine=True, track_running_stats=True) | | |
| (7): Dropout(p=0.5, inplace=False) | | |
| (8): Linear(in_features=300, out_features=300, bias=True) | | |
| (9): ReLU(inplace=True) | | |
| (10): BatchNorm1d(300, eps=1e-05, momentum=0.1, affine=True, track_running_stats=True) | | |
| (11): Dropout(p=0.5, inplace=False) | | |
| (12): Linear(in_features=300, out_features=300, bias=True) | | |
| (13): ReLU(inplace=True) | | |
| (14): BatchNorm1d(300, eps=1e-05, momentum=0.1, affine=True, track_running_stats=True) | | |
| (15): Dropout(p=0.5, inplace=False) | | |
| (16): Linear(in_features=300, out_features=300, bias=True) | | |
| (17): ReLU(inplace=True) | | |
| (18): BatchNorm1d(300, eps=1e-05, momentum=0.1, affine=True, track_running_stats=True) | | |
| (19): Dropout(p=0.5, inplace=False) | | |
| (20): Linear(in_features=300, out_features=2, bias=True) | | |
|  |  |  |
| The total number of parameters = 430636 | | |

**Supplementary Table 8.** Overview of Nuclear Receptors Three Tools can Predict in External Dataset

| **NRs Name** | **NR-Profiler** | **NR-toxpred** | **PbsNRs** | **Data Size** |
| --- | --- | --- | --- | --- |
| **NR1D1** | No | No | Yes | 23 |
| **NR1H3** | Yes | No | Yes | 205 |
| **NR1H4** | Yes | Yes | Yes | 175 |
| **NR1I2** | Yes | No | Yes | 12 |
| **NR2B2** | No | Yes | Yes | 142 |
| **NR2B3** | No | Yes | Yes | 156 |

**Supplementary Materials**

**Supplementary Material 1.** The sequence of NR2B1 for synthesis.

**DNA sequence:**

GGATCCCATCATCATCATCACCATATGGATACCAAACATTTTCTGCCGCTGGATTTTAGCACCCAGGTGAATAGCAGTCTGACCAGCCCGACCGGCCGTGGTAGTATGGCCGCCCCTAGTCTGCATCCGAGCCTGGGTCCGGGTATTGGTAGTCCGGGTCAGCTGCATAGTCCGATTAGTACCCTGAGCAGTCCGATTAATGGCATGGGTCCGCCGTTTAGCGTTATTAGTAGCCCGATGGGCCCGCATAGTATGAGTGTGCCGACCACCCCGACCCTGGGCTTTAGCACCGGCAGCCCGCAGCTGAGCAGTCCTATGAATCCGGTTAGCAGTAGTGAAGATATTAAGCCGCCGCTGGGTCTGAATGGTGTTCTGAAAGTTCCGGCACATCCGAGCGGTAATATGGCAAGCTTTACCAAACATATTTGTGCCATTTGTGGCGATCGCAGCAGTGGTAAACATTATGGTGTGTATAGTTGTGAAGGTTGCAAAGGCTTTTTCAAACGTACCGTGCGCAAAGATCTGACCTATACCTGTCGTGATAATAAGGATTGTCTGATTGATAAACGTCAGCGTAATCGTTGTCAGTATTGTCGCTATCAGAAATGCCTGGCAATGGGCATGAAACGCGAAGCAGTTCAGGAAGAACGCCAGCGCGGTAAAGATCGCAATGAAAATGAAGTTGAAAGTACCAGTAGTGCCAATGAAGATATGCCGGTGGAACGCATTCTGGAAGCCGAACTGGCCGTTGAACCGAAAACCGAAACCTATGTGGAAGCAAATATGGGCCTGAATCCGAGCAGCCCGAATGATCCGGTGACCAATATTTGTCAGGCAGCCGATAAACAGCTGTTTACCCTGGTTGAATGGGCAAAACGTATTCCGCATTTTAGTGAACTGCCGCTGGACGATCAGGTTATTCTGCTGCGTGCAGGTTGGAATGAACTGCTGATTGCCAGTTTTAGCCATCGTAGTATTGCAGTGAAAGATGGCATTCTGCTGGCCACCGGCCTGCATGTTCATCGCAATAGTGCACATAGTGCAGGCGTGGGTGCAATTTTTGATCGCGTTCTGACCGAACTGGTTAGCAAAATGCGTGATATGCAGATGGATAAAACCGAACTGGGCTGCCTGCGCGCAATTGTGCTGTTTAATCCGGATAGTAAAGGTCTGAGCAATCCGGCAGAAGTTGAAGCCCTGCGCGAAAAAGTGTATGCCAGTCTGGAAGCATATTGTAAACATAAATACCCGGAACAGCCGGGCCGCTTTGCAAAACTGCTGCTGCGTCTGCCGGCACTGCGTAGCATTGGCCTGAAATGTCTGGAACATCTGTTTTTCTTTAAGCTGATTGGTGACACCCCGATTGATACCTTTCTGATGGAAATGCTGGAAGCACCGCATCAGATGACCTAACTCGAG

**Protein sequence:**

MSPILGYWKIKGLVQPTRLLLEYLEEKYEEHLYERDEGDKWRNKKFELGLEFPNLPYYIDGDVKLTQSMAIIRYIADKHNMLGGCPKERAEISMLEGAVLDIRYGVSRIAYSKDFETLKVDFLSKLPEMLKMFEDRLCHKTYLNGDHVTHPDFMLYDALDVVLYMDPMCLDAFPKLVCFKKRIEAIPQIDKYLKSSKYIAWPLQGWQATFGGGDHPPKSDLEVLFQGPLGSHHHHHHMDTKHFLPLDFSTQVNSSLTSPTGRGSMAAPSLHPSLGPGIGSPGQLHSPISTLSSPINGMGPPFSVISSPMGPHSMSVPTTPTLGFSTGSPQLSSPMNPVSSSEDIKPPLGLNGVLKVPAHPSGNMASFTKHICAICGDRSSGKHYGVYSCEGCKGFFKRTVRKDLTYTCRDNKDCLIDKRQRNRCQYCRYQKCLAMGMKREAVQEERQRGKDRNENEVESTSSANEDMPVERILEAELAVEPKTETYVEANMGLNPSSPNDPVTNICQAADKQLFTLVEWAKRIPHFSELPLDDQVILLRAGWNELLIASFSHRSIAVKDGILLATGLHVHRNSAHSAGVGAIFDRVLTELVSKMRDMQMDKTELGCLRAIVLFNPDSKGLSNPAEVEALREKVYASLEAYCKHKYPEQPGRFAKLLLRLPALRSIGLKCLEHLFFFKLIGDTPIDTFLMEMLEAPHQMT*

1. Qiu T, Wu D, Qiu J et al. Finding the molecular scaffold of nuclear receptor inhibitors through high-throughput screening based on proteochemometric modelling, J Cheminform 2018;10:21.

2. Nanduri R, Bhutani I, Somavarapu AK et al. ONRLDB--manually curated database of experimentally validated ligands for orphan nuclear receptors: insights into new drug discovery, Database (Oxford) 2015;2015.

3. Chen CY. TCM Database@Taiwan: the world's largest traditional Chinese medicine database for drug screening in silico, PLoS One 2011;6:e15939.

4. Mendez D, Gaulton A, Bento AP et al. ChEMBL: towards direct deposition of bioassay data, Nucleic Acids Res 2019;47:D930-d940.

5. Wishart DS, Feunang YD, Guo AC et al. DrugBank 5.0: a major update to the DrugBank database for 2018, Nucleic Acids Res 2018;46:D1074-d1082.

6. Hussain J, Rea C. Computationally efficient algorithm to identify matched molecular pairs (MMPs) in large data sets, Journal of Chemical Information and Modeling 2010;50:339-348.

7. Bemis GW, Murcko MA. The properties of known drugs. 1. Molecular frameworks, J Med Chem 1996;39:2887-2893.

8. Torstein Hønsi GH, Christer Vasseng, Anita Nesse, Anne Jorunn Fjærestad. Highcharts webpage. <https://www.highcharts.com/>.

9. Karulin B, Kozhevnikov M. Ketcher: web-based chemical structure editor, Journal of Cheminformatics 2011;3:P3.

10. Berman HM, Westbrook J, Feng Z et al. The Protein Data Bank, Nucleic Acids Res 2000;28:235-242.

11. The UniProt C. UniProt: the universal protein knowledgebase, Nucleic Acids Res 2017;45:D158-D169.

12. Pedregosa F, Varoquaux G, Gramfort A et al. Scikit-learn: Machine Learning in Python, Journal of Machine Learning Research 2012;12.
