# Supplementary material for: PbsNRs: predict the potential binders and scaffolds for nuclear receptors
Source: Brief Bioinform. 2025 Jan 11;26(1):bbae710. doi: 10.1093/bib/bbae710 (PMC11724720; doi:10.1093/bib/bbae710)
Supplement: Supplementary_Table_5_bbae710 [file supplementary_table_5_bbae710.docx]

**Supplementary Table 6.** The performance of Random Forest under different parameters.

| Parameter group of random forest | Fold | n_estimators | max_features | max_depth | Train_AUC | Valid_AUC | Test_AUC | Extend_AUC |
| --- | --- | --- | --- | --- | --- | --- | --- | --- |
| 1 | 0 | 101 | 0.1 |  | 0.9997 | 0.8648 | 0.8634 | 0.7287 |
| 2 | 1 | 101 | 0.1 |  | 0.9997 | 0.8710 | 0.8577 | 0.7244 |
| 3 | 2 | 101 | 0.1 |  | 0.9997 | 0.8554 | 0.8523 | 0.7027 |
| 4 | 3 | 101 | 0.1 |  | 0.9997 | 0.8631 | 0.8585 | 0.7174 |
| 5 | 4 | 101 | 0.1 |  | 0.9997 | 0.8355 | 0.8601 | 0.7330 |
| 6 | Mean | 101 | 0.1 |  | 0.9997 | 0.8579 | 0.8584 | 0.7212 |
| 7 | 0 | 101 | 0.1 | 1 | 0.6599 | 0.6196 | 0.6341 | 0.5207 |
| 8 | 1 | 101 | 0.1 | 1 | 0.6653 | 0.6584 | 0.6422 | 0.5318 |
| 9 | 2 | 101 | 0.1 | 1 | 0.6644 | 0.6840 | 0.6472 | 0.5350 |
| 10 | 3 | 101 | 0.1 | 1 | 0.6566 | 0.6355 | 0.6353 | 0.5314 |
| 11 | 4 | 101 | 0.1 | 1 | 0.6673 | 0.6461 | 0.6394 | 0.5570 |
| 12 | Mean | 101 | 0.1 | 1 | 0.6627 | 0.6487 | 0.6397 | 0.5352 |
| 13 | 0 | 101 | 0.1 | 2 | 0.7104 | 0.6645 | 0.6684 | 0.6137 |
| 14 | 1 | 101 | 0.1 | 2 | 0.7090 | 0.6960 | 0.6687 | 0.6195 |
| 15 | 2 | 101 | 0.1 | 2 | 0.7134 | 0.7085 | 0.6750 | 0.6317 |
| 16 | 3 | 101 | 0.1 | 2 | 0.7182 | 0.6868 | 0.6782 | 0.6329 |
| 17 | 4 | 101 | 0.1 | 2 | 0.7164 | 0.6894 | 0.6683 | 0.6425 |
| 18 | Mean | 101 | 0.1 | 2 | 0.7135 | 0.6891 | 0.6717 | 0.6281 |
| 19 | 0 | 101 | 0.1 | 3 | 0.7556 | 0.7147 | 0.7020 | 0.6542 |
| 20 | 1 | 101 | 0.1 | 3 | 0.7523 | 0.7231 | 0.6971 | 0.6666 |
| 21 | 2 | 101 | 0.1 | 3 | 0.7537 | 0.7363 | 0.7061 | 0.6591 |
| 22 | 3 | 101 | 0.1 | 3 | 0.7565 | 0.7153 | 0.7001 | 0.6626 |
| 23 | 4 | 101 | 0.1 | 3 | 0.7573 | 0.7215 | 0.7004 | 0.6631 |
| 24 | Mean | 101 | 0.1 | 3 | 0.7551 | 0.7222 | 0.7011 | 0.6612 |
| 25 | 0 | 101 | 0.1 | 4 | 0.7944 | 0.7382 | 0.7292 | 0.6739 |
| 26 | 1 | 101 | 0.1 | 4 | 0.8074 | 0.7626 | 0.7426 | 0.6725 |
| 27 | 2 | 101 | 0.1 | 4 | 0.8089 | 0.7734 | 0.7470 | 0.6739 |
| 28 | 3 | 101 | 0.1 | 4 | 0.8072 | 0.7490 | 0.7358 | 0.6802 |
| 29 | 4 | 101 | 0.1 | 4 | 0.8031 | 0.7449 | 0.7350 | 0.6717 |
| 30 | Mean | 101 | 0.1 | 4 | 0.8042 | 0.7536 | 0.7379 | 0.6745 |
| 31 | 0 | 101 | 0.1 | 5 | 0.8464 | 0.7759 | 0.7649 | 0.6967 |
| 32 | 1 | 101 | 0.1 | 5 | 0.8442 | 0.7824 | 0.7639 | 0.6748 |
| 33 | 2 | 101 | 0.1 | 5 | 0.8460 | 0.7890 | 0.7648 | 0.6753 |
| 34 | 3 | 101 | 0.1 | 5 | 0.8529 | 0.7850 | 0.7710 | 0.6745 |
| 35 | 4 | 101 | 0.1 | 5 | 0.8498 | 0.7702 | 0.7656 | 0.6729 |
| 36 | Mean | 101 | 0.1 | 5 | 0.8478 | 0.7805 | 0.7660 | 0.6788 |
| 37 | 0 | 101 | 0.2 |  | 0.9997 | 0.8642 | 0.8588 | 0.7356 |
| 38 | 1 | 101 | 0.2 |  | 0.9997 | 0.8639 | 0.8510 | 0.7297 |
| 39 | 2 | 101 | 0.2 |  | 0.9997 | 0.8557 | 0.8536 | 0.7110 |
| 40 | 3 | 101 | 0.2 |  | 0.9997 | 0.8637 | 0.8546 | 0.7243 |
| 41 | 4 | 101 | 0.2 |  | 0.9996 | 0.8320 | 0.8557 | 0.7185 |
| 42 | Mean | 101 | 0.2 |  | 0.9997 | 0.8559 | 0.8547 | 0.7238 |
| 43 | 0 | 101 | 0.2 | 1 | 0.6609 | 0.6173 | 0.6312 | 0.5516 |
| 44 | 1 | 101 | 0.2 | 1 | 0.6652 | 0.6553 | 0.6404 | 0.5556 |
| 45 | 2 | 101 | 0.2 | 1 | 0.6480 | 0.6654 | 0.6279 | 0.5506 |
| 46 | 3 | 101 | 0.2 | 1 | 0.6582 | 0.6321 | 0.6289 | 0.5383 |
| 47 | 4 | 101 | 0.2 | 1 | 0.6540 | 0.6402 | 0.6264 | 0.5557 |
| 48 | Mean | 101 | 0.2 | 1 | 0.6572 | 0.6420 | 0.6310 | 0.5504 |
| 49 | 0 | 101 | 0.2 | 2 | 0.7050 | 0.6739 | 0.6627 | 0.6114 |
| 50 | 1 | 101 | 0.2 | 2 | 0.7077 | 0.6975 | 0.6740 | 0.5962 |
| 51 | 2 | 101 | 0.2 | 2 | 0.7100 | 0.7102 | 0.6677 | 0.6241 |
| 52 | 3 | 101 | 0.2 | 2 | 0.7188 | 0.6822 | 0.6732 | 0.6298 |
| 53 | 4 | 101 | 0.2 | 2 | 0.7074 | 0.6854 | 0.6553 | 0.6436 |
| 54 | Mean | 101 | 0.2 | 2 | 0.7098 | 0.6898 | 0.6666 | 0.6210 |
| 55 | 0 | 101 | 0.2 | 3 | 0.7538 | 0.7090 | 0.6982 | 0.6541 |
| 56 | 1 | 101 | 0.2 | 3 | 0.7582 | 0.7302 | 0.7093 | 0.6544 |
| 57 | 2 | 101 | 0.2 | 3 | 0.7588 | 0.7463 | 0.7104 | 0.6570 |
| 58 | 3 | 101 | 0.2 | 3 | 0.7685 | 0.7254 | 0.7103 | 0.6775 |
| 59 | 4 | 101 | 0.2 | 3 | 0.7617 | 0.7197 | 0.7005 | 0.6592 |
| 60 | Mean | 101 | 0.2 | 3 | 0.7602 | 0.7261 | 0.7057 | 0.6605 |
| 61 | 0 | 101 | 0.2 | 4 | 0.8051 | 0.7482 | 0.7407 | 0.6884 |
| 62 | 1 | 101 | 0.2 | 4 | 0.8079 | 0.7679 | 0.7467 | 0.6710 |
| 63 | 2 | 101 | 0.2 | 4 | 0.8145 | 0.7739 | 0.7531 | 0.6795 |
| 64 | 3 | 101 | 0.2 | 4 | 0.8147 | 0.7564 | 0.7460 | 0.6734 |
| 65 | 4 | 101 | 0.2 | 4 | 0.8091 | 0.7472 | 0.7348 | 0.6691 |
| 66 | Mean | 101 | 0.2 | 4 | 0.8102 | 0.7587 | 0.7442 | 0.6763 |
| 67 | 0 | 101 | 0.2 | 5 | 0.8581 | 0.7880 | 0.7757 | 0.6811 |
| 68 | 1 | 101 | 0.2 | 5 | 0.8557 | 0.7962 | 0.7689 | 0.6679 |
| 69 | 2 | 101 | 0.2 | 5 | 0.8565 | 0.7935 | 0.7769 | 0.6632 |
| 70 | 3 | 101 | 0.2 | 5 | 0.8600 | 0.7822 | 0.7774 | 0.6829 |
| 71 | 4 | 101 | 0.2 | 5 | 0.8612 | 0.7705 | 0.7728 | 0.6809 |
| 72 | Mean | 101 | 0.2 | 5 | 0.8583 | 0.7861 | 0.7744 | 0.6752 |
| 73 | 0 | 101 | 0.3 |  | 0.9997 | 0.8638 | 0.8596 | 0.7325 |
| 74 | 1 | 101 | 0.3 |  | 0.9997 | 0.8633 | 0.8500 | 0.7213 |
| 75 | 2 | 101 | 0.3 |  | 0.9997 | 0.8460 | 0.8459 | 0.6920 |
| 76 | 3 | 101 | 0.3 |  | 0.9997 | 0.8650 | 0.8526 | 0.7252 |
| 77 | 4 | 101 | 0.3 |  | 0.9996 | 0.8324 | 0.8512 | 0.7238 |
| 78 | Mean | 101 | 0.3 |  | 0.9997 | 0.8541 | 0.8519 | 0.7190 |
| 79 | 0 | 101 | 0.3 | 1 | 0.6564 | 0.6255 | 0.6300 | 0.5670 |
| 80 | 1 | 101 | 0.3 | 1 | 0.6509 | 0.6402 | 0.6297 | 0.5319 |
| 81 | 2 | 101 | 0.3 | 1 | 0.6500 | 0.6682 | 0.6276 | 0.5441 |
| 82 | 3 | 101 | 0.3 | 1 | 0.6588 | 0.6239 | 0.6337 | 0.5109 |
| 83 | 4 | 101 | 0.3 | 1 | 0.6669 | 0.6566 | 0.6409 | 0.5275 |
| 84 | Mean | 101 | 0.3 | 1 | 0.6566 | 0.6429 | 0.6324 | 0.5363 |
| 85 | 0 | 101 | 0.3 | 2 | 0.7040 | 0.6734 | 0.6554 | 0.6192 |
| 86 | 1 | 101 | 0.3 | 2 | 0.7100 | 0.7036 | 0.6727 | 0.6167 |
| 87 | 2 | 101 | 0.3 | 2 | 0.7152 | 0.7048 | 0.6739 | 0.6502 |
| 88 | 3 | 101 | 0.3 | 2 | 0.7158 | 0.6766 | 0.6694 | 0.6180 |
| 89 | 4 | 101 | 0.3 | 2 | 0.7082 | 0.6821 | 0.6623 | 0.6440 |
| 90 | Mean | 101 | 0.3 | 2 | 0.7106 | 0.6881 | 0.6667 | 0.6296 |
| 91 | 0 | 101 | 0.3 | 3 | 0.7557 | 0.7167 | 0.7018 | 0.6622 |
| 92 | 1 | 101 | 0.3 | 3 | 0.7642 | 0.7365 | 0.7129 | 0.6565 |
| 93 | 2 | 101 | 0.3 | 3 | 0.7589 | 0.7317 | 0.7059 | 0.6590 |
| 94 | 3 | 101 | 0.3 | 3 | 0.7638 | 0.7160 | 0.7037 | 0.6693 |
| 95 | 4 | 101 | 0.3 | 3 | 0.7551 | 0.7113 | 0.6921 | 0.6525 |
| 96 | Mean | 101 | 0.3 | 3 | 0.7595 | 0.7224 | 0.7033 | 0.6599 |
| 97 | 0 | 101 | 0.3 | 4 | 0.8101 | 0.7584 | 0.7452 | 0.6714 |
| 98 | 1 | 101 | 0.3 | 4 | 0.8084 | 0.7667 | 0.7416 | 0.6729 |
| 99 | 2 | 101 | 0.3 | 4 | 0.8130 | 0.7747 | 0.7504 | 0.6777 |
| 100 | 3 | 101 | 0.3 | 4 | 0.8149 | 0.7517 | 0.7436 | 0.6825 |
| 101 | 4 | 101 | 0.3 | 4 | 0.8174 | 0.7548 | 0.7416 | 0.6748 |
| 102 | Mean | 101 | 0.3 | 4 | 0.8128 | 0.7613 | 0.7445 | 0.6758 |
| 103 | 0 | 101 | 0.3 | 5 | 0.8622 | 0.7912 | 0.7781 | 0.6897 |
| 104 | 1 | 101 | 0.3 | 5 | 0.8498 | 0.7869 | 0.7667 | 0.6808 |
| 105 | 2 | 101 | 0.3 | 5 | 0.8561 | 0.7947 | 0.7750 | 0.6785 |
| 106 | 3 | 101 | 0.3 | 5 | 0.8621 | 0.7818 | 0.7755 | 0.6949 |
| 107 | 4 | 101 | 0.3 | 5 | 0.8609 | 0.7714 | 0.7713 | 0.6745 |
| 108 | Mean | 101 | 0.3 | 5 | 0.8582 | 0.7852 | 0.7733 | 0.6837 |
| 109 | 0 | 101 | 0.4 |  | 0.9997 | 0.8571 | 0.8533 | 0.7394 |
| 110 | 1 | 101 | 0.4 |  | 0.9996 | 0.8601 | 0.8468 | 0.7214 |
| 111 | 2 | 101 | 0.4 |  | 0.9997 | 0.8547 | 0.8501 | 0.6985 |
| 112 | 3 | 101 | 0.4 |  | 0.9997 | 0.8650 | 0.8548 | 0.7254 |
| 113 | 4 | 101 | 0.4 |  | 0.9997 | 0.8298 | 0.8515 | 0.7255 |
| 114 | Mean | 101 | 0.4 |  | 0.9997 | 0.8533 | 0.8513 | 0.7220 |
| 115 | 0 | 101 | 0.4 | 1 | 0.6380 | 0.6020 | 0.6116 | 0.5307 |
| 116 | 1 | 101 | 0.4 | 1 | 0.6420 | 0.6433 | 0.6193 | 0.5714 |
| 117 | 2 | 101 | 0.4 | 1 | 0.6676 | 0.6750 | 0.6434 | 0.5238 |
| 118 | 3 | 101 | 0.4 | 1 | 0.6646 | 0.6255 | 0.6366 | 0.5394 |
| 119 | 4 | 101 | 0.4 | 1 | 0.6591 | 0.6444 | 0.6327 | 0.5803 |
| 120 | Mean | 101 | 0.4 | 1 | 0.6542 | 0.6381 | 0.6287 | 0.5491 |
| 121 | 0 | 101 | 0.4 | 2 | 0.7052 | 0.6773 | 0.6621 | 0.6182 |
| 122 | 1 | 101 | 0.4 | 2 | 0.7020 | 0.6984 | 0.6654 | 0.6087 |
| 123 | 2 | 101 | 0.4 | 2 | 0.7110 | 0.6985 | 0.6658 | 0.6214 |
| 124 | 3 | 101 | 0.4 | 2 | 0.7166 | 0.6707 | 0.6712 | 0.6244 |
| 125 | 4 | 101 | 0.4 | 2 | 0.7028 | 0.6739 | 0.6552 | 0.6297 |
| 126 | Mean | 101 | 0.4 | 2 | 0.7075 | 0.6838 | 0.6639 | 0.6205 |
| 127 | 0 | 101 | 0.4 | 3 | 0.7595 | 0.7229 | 0.7028 | 0.6590 |
| 128 | 1 | 101 | 0.4 | 3 | 0.7684 | 0.7401 | 0.7164 | 0.6640 |
| 129 | 2 | 101 | 0.4 | 3 | 0.7652 | 0.7481 | 0.7150 | 0.6499 |
| 130 | 3 | 101 | 0.4 | 3 | 0.7616 | 0.7105 | 0.7071 | 0.6316 |
| 131 | 4 | 101 | 0.4 | 3 | 0.7565 | 0.7140 | 0.6999 | 0.6707 |
| 132 | Mean | 101 | 0.4 | 3 | 0.7622 | 0.7271 | 0.7082 | 0.6550 |
| 133 | 0 | 101 | 0.4 | 4 | 0.8110 | 0.7622 | 0.7465 | 0.6818 |
| 134 | 1 | 101 | 0.4 | 4 | 0.8146 | 0.7705 | 0.7467 | 0.6645 |
| 135 | 2 | 101 | 0.4 | 4 | 0.8182 | 0.7795 | 0.7555 | 0.6647 |
| 136 | 3 | 101 | 0.4 | 4 | 0.8142 | 0.7534 | 0.7462 | 0.6664 |
| 137 | 4 | 101 | 0.4 | 4 | 0.8102 | 0.7474 | 0.7394 | 0.6731 |
| 138 | Mean | 101 | 0.4 | 4 | 0.8136 | 0.7626 | 0.7469 | 0.6701 |
| 139 | 0 | 101 | 0.4 | 5 | 0.8594 | 0.7867 | 0.7744 | 0.6930 |
| 140 | 1 | 101 | 0.4 | 5 | 0.8560 | 0.7937 | 0.7705 | 0.6721 |
| 141 | 2 | 101 | 0.4 | 5 | 0.8593 | 0.7940 | 0.7810 | 0.6712 |
| 142 | 3 | 101 | 0.4 | 5 | 0.8648 | 0.7820 | 0.7752 | 0.6793 |
| 143 | 4 | 101 | 0.4 | 5 | 0.8611 | 0.7716 | 0.7726 | 0.6640 |
| 144 | Mean | 101 | 0.4 | 5 | 0.8601 | 0.7856 | 0.7747 | 0.6759 |
| 145 | 0 | 101 | 0.5 |  | 0.9997 | 0.8543 | 0.8540 | 0.7187 |
| 146 | 1 | 101 | 0.5 |  | 0.9996 | 0.8611 | 0.8424 | 0.7148 |
| 147 | 2 | 101 | 0.5 |  | 0.9996 | 0.8452 | 0.8409 | 0.6939 |
| 148 | 3 | 101 | 0.5 |  | 0.9997 | 0.8622 | 0.8478 | 0.7243 |
| 149 | 4 | 101 | 0.5 |  | 0.9996 | 0.8336 | 0.8496 | 0.7233 |
| 150 | Mean | 101 | 0.5 |  | 0.9996 | 0.8513 | 0.8469 | 0.7150 |
| 151 | 0 | 101 | 0.5 | 1 | 0.6527 | 0.6188 | 0.6232 | 0.5575 |
| 152 | 1 | 101 | 0.5 | 1 | 0.6453 | 0.6476 | 0.6222 | 0.5925 |
| 153 | 2 | 101 | 0.5 | 1 | 0.6483 | 0.6662 | 0.6237 | 0.5609 |
| 154 | 3 | 101 | 0.5 | 1 | 0.6614 | 0.6297 | 0.6336 | 0.5734 |
| 155 | 4 | 101 | 0.5 | 1 | 0.6658 | 0.6472 | 0.6372 | 0.5741 |
| 156 | Mean | 101 | 0.5 | 1 | 0.6547 | 0.6419 | 0.6280 | 0.5717 |
| 157 | 0 | 101 | 0.5 | 2 | 0.7048 | 0.6793 | 0.6584 | 0.6215 |
| 158 | 1 | 101 | 0.5 | 2 | 0.7082 | 0.7013 | 0.6707 | 0.5938 |
| 159 | 2 | 101 | 0.5 | 2 | 0.7086 | 0.7008 | 0.6615 | 0.6163 |
| 160 | 3 | 101 | 0.5 | 2 | 0.7072 | 0.6655 | 0.6586 | 0.6197 |
| 161 | 4 | 101 | 0.5 | 2 | 0.7125 | 0.6829 | 0.6645 | 0.6344 |
| 162 | Mean | 101 | 0.5 | 2 | 0.7083 | 0.6860 | 0.6628 | 0.6171 |
| 163 | 0 | 101 | 0.5 | 3 | 0.7595 | 0.7290 | 0.7041 | 0.6597 |
| 164 | 1 | 101 | 0.5 | 3 | 0.7619 | 0.7315 | 0.7123 | 0.6525 |
| 165 | 2 | 101 | 0.5 | 3 | 0.7581 | 0.7369 | 0.7046 | 0.6464 |
| 166 | 3 | 101 | 0.5 | 3 | 0.7610 | 0.7058 | 0.6981 | 0.6473 |
| 167 | 4 | 101 | 0.5 | 3 | 0.7565 | 0.7198 | 0.6980 | 0.6490 |
| 168 | Mean | 101 | 0.5 | 3 | 0.7594 | 0.7246 | 0.7034 | 0.6510 |
| 169 | 0 | 101 | 0.5 | 4 | 0.8144 | 0.7635 | 0.7406 | 0.6751 |
| 170 | 1 | 101 | 0.5 | 4 | 0.8111 | 0.7659 | 0.7443 | 0.6644 |
| 171 | 2 | 101 | 0.5 | 4 | 0.8102 | 0.7739 | 0.7427 | 0.6450 |
| 172 | 3 | 101 | 0.5 | 4 | 0.8176 | 0.7528 | 0.7422 | 0.6688 |
| 173 | 4 | 101 | 0.5 | 4 | 0.8131 | 0.7561 | 0.7414 | 0.6687 |
| 174 | Mean | 101 | 0.5 | 4 | 0.8133 | 0.7624 | 0.7423 | 0.6644 |
| 175 | 0 | 101 | 0.5 | 5 | 0.8637 | 0.7951 | 0.7806 | 0.6847 |
| 176 | 1 | 101 | 0.5 | 5 | 0.8595 | 0.7985 | 0.7754 | 0.6751 |
| 177 | 2 | 101 | 0.5 | 5 | 0.8657 | 0.8012 | 0.7771 | 0.6679 |
| 178 | 3 | 101 | 0.5 | 5 | 0.8572 | 0.7757 | 0.7696 | 0.6622 |
| 179 | 4 | 101 | 0.5 | 5 | 0.8605 | 0.7717 | 0.7701 | 0.6611 |
| 180 | Mean | 101 | 0.5 | 5 | 0.8613 | 0.7884 | 0.7746 | 0.6702 |
| 181 | 0 | 301 | 0.1 |  | 0.9997 | 0.8685 | 0.8680 | 0.7316 |
| 182 | 1 | 301 | 0.1 |  | 0.9997 | 0.8718 | 0.8576 | 0.7247 |
| 183 | 2 | 301 | 0.1 |  | 0.9997 | 0.8624 | 0.8559 | 0.6974 |
| 184 | 3 | 301 | 0.1 |  | 0.9997 | 0.8727 | 0.8609 | 0.7236 |
| 185 | 4 | 301 | 0.1 |  | 0.9997 | 0.8413 | 0.8606 | 0.7253 |
| 186 | Mean | 301 | 0.1 |  | 0.9997 | 0.8633 | 0.8606 | 0.7205 |
| 187 | 0 | 301 | 0.1 | 1 | 0.6710 | 0.6267 | 0.6372 | 0.5594 |
| 188 | 1 | 301 | 0.1 | 1 | 0.6702 | 0.6573 | 0.6409 | 0.5236 |
| 189 | 2 | 301 | 0.1 | 1 | 0.6698 | 0.6975 | 0.6504 | 0.5388 |
| 190 | 3 | 301 | 0.1 | 1 | 0.6700 | 0.6360 | 0.6442 | 0.5241 |
| 191 | 4 | 301 | 0.1 | 1 | 0.6787 | 0.6626 | 0.6438 | 0.5548 |
| 192 | Mean | 301 | 0.1 | 1 | 0.6719 | 0.6560 | 0.6433 | 0.5402 |
| 193 | 0 | 301 | 0.1 | 2 | 0.7081 | 0.6692 | 0.6636 | 0.6123 |
| 194 | 1 | 301 | 0.1 | 2 | 0.7161 | 0.7009 | 0.6764 | 0.6344 |
| 195 | 2 | 301 | 0.1 | 2 | 0.7181 | 0.7148 | 0.6802 | 0.6438 |
| 196 | 3 | 301 | 0.1 | 2 | 0.7210 | 0.6882 | 0.6768 | 0.6276 |
| 197 | 4 | 301 | 0.1 | 2 | 0.7159 | 0.6834 | 0.6684 | 0.6309 |
| 198 | Mean | 301 | 0.1 | 2 | 0.7158 | 0.6913 | 0.6731 | 0.6298 |
| 199 | 0 | 301 | 0.1 | 3 | 0.7508 | 0.7056 | 0.6942 | 0.6581 |
| 200 | 1 | 301 | 0.1 | 3 | 0.7574 | 0.7274 | 0.7052 | 0.6646 |
| 201 | 2 | 301 | 0.1 | 3 | 0.7569 | 0.7379 | 0.7072 | 0.6657 |
| 202 | 3 | 301 | 0.1 | 3 | 0.7635 | 0.7189 | 0.7075 | 0.6759 |
| 203 | 4 | 301 | 0.1 | 3 | 0.7557 | 0.7162 | 0.6935 | 0.6677 |
| 204 | Mean | 301 | 0.1 | 3 | 0.7569 | 0.7212 | 0.7015 | 0.6664 |
| 205 | 0 | 301 | 0.1 | 4 | 0.7998 | 0.7444 | 0.7301 | 0.6922 |
| 206 | 1 | 301 | 0.1 | 4 | 0.8020 | 0.7578 | 0.7377 | 0.6667 |
| 207 | 2 | 301 | 0.1 | 4 | 0.8095 | 0.7710 | 0.7451 | 0.6711 |
| 208 | 3 | 301 | 0.1 | 4 | 0.8094 | 0.7556 | 0.7415 | 0.6887 |
| 209 | 4 | 301 | 0.1 | 4 | 0.8080 | 0.7492 | 0.7394 | 0.6780 |
| 210 | Mean | 301 | 0.1 | 4 | 0.8057 | 0.7556 | 0.7388 | 0.6793 |
| 211 | 0 | 301 | 0.1 | 5 | 0.8481 | 0.7744 | 0.7651 | 0.6914 |
| 212 | 1 | 301 | 0.1 | 5 | 0.8463 | 0.7866 | 0.7671 | 0.6847 |
| 213 | 2 | 301 | 0.1 | 5 | 0.8507 | 0.7971 | 0.7717 | 0.6684 |
| 214 | 3 | 301 | 0.1 | 5 | 0.8534 | 0.7832 | 0.7701 | 0.6863 |
| 215 | 4 | 301 | 0.1 | 5 | 0.8543 | 0.7719 | 0.7703 | 0.6853 |
| 216 | Mean | 301 | 0.1 | 5 | 0.8506 | 0.7827 | 0.7688 | 0.6832 |
| 217 | 0 | 301 | 0.2 |  | 0.9997 | 0.8641 | 0.8618 | 0.7342 |
| 218 | 1 | 301 | 0.2 |  | 0.9997 | 0.8695 | 0.8521 | 0.7232 |
| 219 | 2 | 301 | 0.2 |  | 0.9997 | 0.8561 | 0.8536 | 0.6983 |
| 220 | 3 | 301 | 0.2 |  | 0.9997 | 0.8732 | 0.8568 | 0.7253 |
| 221 | 4 | 301 | 0.2 |  | 0.9997 | 0.8375 | 0.8553 | 0.7248 |
| 222 | Mean | 301 | 0.2 |  | 0.9997 | 0.8601 | 0.8559 | 0.7212 |
| 223 | 0 | 301 | 0.2 | 1 | 0.6534 | 0.6083 | 0.6272 | 0.5432 |
| 224 | 1 | 301 | 0.2 | 1 | 0.6625 | 0.6547 | 0.6418 | 0.5236 |
| 225 | 2 | 301 | 0.2 | 1 | 0.6653 | 0.6834 | 0.6442 | 0.5370 |
| 226 | 3 | 301 | 0.2 | 1 | 0.6661 | 0.6347 | 0.6381 | 0.5332 |
| 227 | 4 | 301 | 0.2 | 1 | 0.6671 | 0.6499 | 0.6402 | 0.5937 |
| 228 | Mean | 301 | 0.2 | 1 | 0.6629 | 0.6462 | 0.6383 | 0.5462 |
| 229 | 0 | 301 | 0.2 | 2 | 0.7047 | 0.6708 | 0.6605 | 0.6151 |
| 230 | 1 | 301 | 0.2 | 2 | 0.7083 | 0.6979 | 0.6712 | 0.6169 |
| 231 | 2 | 301 | 0.2 | 2 | 0.7108 | 0.7066 | 0.6683 | 0.6323 |
| 232 | 3 | 301 | 0.2 | 2 | 0.7139 | 0.6760 | 0.6708 | 0.6231 |
| 233 | 4 | 301 | 0.2 | 2 | 0.7097 | 0.6846 | 0.6648 | 0.6443 |
| 234 | Mean | 301 | 0.2 | 2 | 0.7095 | 0.6872 | 0.6671 | 0.6263 |
| 235 | 0 | 301 | 0.2 | 3 | 0.7564 | 0.7168 | 0.7029 | 0.6544 |
| 236 | 1 | 301 | 0.2 | 3 | 0.7585 | 0.7292 | 0.7067 | 0.6641 |
| 237 | 2 | 301 | 0.2 | 3 | 0.7587 | 0.7405 | 0.7091 | 0.6632 |
| 238 | 3 | 301 | 0.2 | 3 | 0.7639 | 0.7175 | 0.7087 | 0.6706 |
| 239 | 4 | 301 | 0.2 | 3 | 0.7551 | 0.7148 | 0.6984 | 0.6638 |
| 240 | Mean | 301 | 0.2 | 3 | 0.7585 | 0.7238 | 0.7051 | 0.6632 |
| 241 | 0 | 301 | 0.2 | 4 | 0.8093 | 0.7572 | 0.7415 | 0.6767 |
| 242 | 1 | 301 | 0.2 | 4 | 0.8112 | 0.7685 | 0.7449 | 0.6700 |
| 243 | 2 | 301 | 0.2 | 4 | 0.8150 | 0.7779 | 0.7534 | 0.6750 |
| 244 | 3 | 301 | 0.2 | 4 | 0.8191 | 0.7594 | 0.7490 | 0.6850 |
| 245 | 4 | 301 | 0.2 | 4 | 0.8113 | 0.7478 | 0.7416 | 0.6763 |
| 246 | Mean | 301 | 0.2 | 4 | 0.8132 | 0.7622 | 0.7461 | 0.6766 |
| 247 | 0 | 301 | 0.2 | 5 | 0.8570 | 0.7836 | 0.7731 | 0.6906 |
| 248 | 1 | 301 | 0.2 | 5 | 0.8531 | 0.7891 | 0.7691 | 0.6765 |
| 249 | 2 | 301 | 0.2 | 5 | 0.8571 | 0.7976 | 0.7787 | 0.6768 |
| 250 | 3 | 301 | 0.2 | 5 | 0.8606 | 0.7911 | 0.7768 | 0.6852 |
| 251 | 4 | 301 | 0.2 | 5 | 0.8582 | 0.7733 | 0.7721 | 0.6798 |
| 252 | Mean | 301 | 0.2 | 5 | 0.8572 | 0.7869 | 0.7740 | 0.6818 |
| 253 | 0 | 301 | 0.3 |  | 0.9997 | 0.8602 | 0.8605 | 0.7252 |
| 254 | 1 | 301 | 0.3 |  | 0.9997 | 0.8663 | 0.8512 | 0.7242 |
| 255 | 2 | 301 | 0.3 |  | 0.9997 | 0.8544 | 0.8495 | 0.7022 |
| 256 | 3 | 301 | 0.3 |  | 0.9997 | 0.8631 | 0.8536 | 0.7233 |
| 257 | 4 | 301 | 0.3 |  | 0.9997 | 0.8329 | 0.8535 | 0.7257 |
| 258 | Mean | 301 | 0.3 |  | 0.9997 | 0.8554 | 0.8537 | 0.7201 |
| 259 | 0 | 301 | 0.3 | 1 | 0.6497 | 0.6131 | 0.6178 | 0.5511 |
| 260 | 1 | 301 | 0.3 | 1 | 0.6591 | 0.6507 | 0.6370 | 0.5309 |
| 261 | 2 | 301 | 0.3 | 1 | 0.6653 | 0.6810 | 0.6446 | 0.5430 |
| 262 | 3 | 301 | 0.3 | 1 | 0.6616 | 0.6283 | 0.6329 | 0.5418 |
| 263 | 4 | 301 | 0.3 | 1 | 0.6624 | 0.6481 | 0.6349 | 0.5680 |
| 264 | Mean | 301 | 0.3 | 1 | 0.6596 | 0.6442 | 0.6335 | 0.5470 |
| 265 | 0 | 301 | 0.3 | 2 | 0.7042 | 0.6711 | 0.6622 | 0.6185 |
| 266 | 1 | 301 | 0.3 | 2 | 0.7069 | 0.6996 | 0.6689 | 0.6110 |
| 267 | 2 | 301 | 0.3 | 2 | 0.7108 | 0.7084 | 0.6699 | 0.6279 |
| 268 | 3 | 301 | 0.3 | 2 | 0.7167 | 0.6796 | 0.6712 | 0.6339 |
| 269 | 4 | 301 | 0.3 | 2 | 0.7028 | 0.6759 | 0.6607 | 0.6235 |
| 270 | Mean | 301 | 0.3 | 2 | 0.7083 | 0.6869 | 0.6666 | 0.6230 |
| 271 | 0 | 301 | 0.3 | 3 | 0.7595 | 0.7237 | 0.7054 | 0.6658 |
| 272 | 1 | 301 | 0.3 | 3 | 0.7626 | 0.7389 | 0.7107 | 0.6659 |
| 273 | 2 | 301 | 0.3 | 3 | 0.7597 | 0.7386 | 0.7105 | 0.6579 |
| 274 | 3 | 301 | 0.3 | 3 | 0.7658 | 0.7114 | 0.7065 | 0.6592 |
| 275 | 4 | 301 | 0.3 | 3 | 0.7596 | 0.7184 | 0.7029 | 0.6641 |
| 276 | Mean | 301 | 0.3 | 3 | 0.7614 | 0.7262 | 0.7072 | 0.6626 |
| 277 | 0 | 301 | 0.3 | 4 | 0.8145 | 0.7632 | 0.7456 | 0.6707 |
| 278 | 1 | 301 | 0.3 | 4 | 0.8149 | 0.7742 | 0.7468 | 0.6734 |
| 279 | 2 | 301 | 0.3 | 4 | 0.8149 | 0.7741 | 0.7511 | 0.6736 |
| 280 | 3 | 301 | 0.3 | 4 | 0.8158 | 0.7497 | 0.7464 | 0.6775 |
| 281 | 4 | 301 | 0.3 | 4 | 0.8154 | 0.7509 | 0.7435 | 0.6680 |
| 282 | Mean | 301 | 0.3 | 4 | 0.8151 | 0.7624 | 0.7467 | 0.6726 |
| 283 | 0 | 301 | 0.3 | 5 | 0.8632 | 0.7899 | 0.7785 | 0.6825 |
| 284 | 1 | 301 | 0.3 | 5 | 0.8597 | 0.7966 | 0.7737 | 0.6788 |
| 285 | 2 | 301 | 0.3 | 5 | 0.8623 | 0.8025 | 0.7795 | 0.6795 |
| 286 | 3 | 301 | 0.3 | 5 | 0.8629 | 0.7868 | 0.7760 | 0.6816 |
| 287 | 4 | 301 | 0.3 | 5 | 0.8610 | 0.7751 | 0.7731 | 0.6738 |
| 288 | Mean | 301 | 0.3 | 5 | 0.8618 | 0.7902 | 0.7762 | 0.6792 |
| 289 | 0 | 301 | 0.4 |  | 0.9997 | 0.8629 | 0.8586 | 0.7325 |
| 290 | 1 | 301 | 0.4 |  | 0.9997 | 0.8609 | 0.8495 | 0.7232 |
| 291 | 2 | 301 | 0.4 |  | 0.9997 | 0.8495 | 0.8480 | 0.6980 |
| 292 | 3 | 301 | 0.4 |  | 0.9997 | 0.8635 | 0.8533 | 0.7307 |
| 293 | 4 | 301 | 0.4 |  | 0.9997 | 0.8326 | 0.8520 | 0.7167 |
| 294 | Mean | 301 | 0.4 |  | 0.9997 | 0.8539 | 0.8523 | 0.7202 |
| 295 | 0 | 301 | 0.4 | 1 | 0.6614 | 0.6289 | 0.6337 | 0.5542 |
| 296 | 1 | 301 | 0.4 | 1 | 0.6547 | 0.6436 | 0.6297 | 0.5350 |
| 297 | 2 | 301 | 0.4 | 1 | 0.6578 | 0.6828 | 0.6399 | 0.5500 |
| 298 | 3 | 301 | 0.4 | 1 | 0.6643 | 0.6292 | 0.6366 | 0.5457 |
| 299 | 4 | 301 | 0.4 | 1 | 0.6609 | 0.6468 | 0.6367 | 0.5740 |
| 300 | Mean | 301 | 0.4 | 1 | 0.6598 | 0.6463 | 0.6353 | 0.5518 |
| 301 | 0 | 301 | 0.4 | 2 | 0.7065 | 0.6771 | 0.6631 | 0.6181 |
| 302 | 1 | 301 | 0.4 | 2 | 0.7061 | 0.7039 | 0.6681 | 0.6252 |
| 303 | 2 | 301 | 0.4 | 2 | 0.7096 | 0.7066 | 0.6686 | 0.6342 |
| 304 | 3 | 301 | 0.4 | 2 | 0.7166 | 0.6751 | 0.6700 | 0.6327 |
| 305 | 4 | 301 | 0.4 | 2 | 0.7057 | 0.6806 | 0.6584 | 0.6328 |
| 306 | Mean | 301 | 0.4 | 2 | 0.7089 | 0.6887 | 0.6657 | 0.6286 |
| 307 | 0 | 301 | 0.4 | 3 | 0.7587 | 0.7217 | 0.7029 | 0.6534 |
| 308 | 1 | 301 | 0.4 | 3 | 0.7617 | 0.7372 | 0.7103 | 0.6522 |
| 309 | 2 | 301 | 0.4 | 3 | 0.7612 | 0.7395 | 0.7077 | 0.6651 |
| 310 | 3 | 301 | 0.4 | 3 | 0.7663 | 0.7156 | 0.7058 | 0.6553 |
| 311 | 4 | 301 | 0.4 | 3 | 0.7590 | 0.7177 | 0.7018 | 0.6660 |
| 312 | Mean | 301 | 0.4 | 3 | 0.7614 | 0.7263 | 0.7057 | 0.6584 |
| 313 | 0 | 301 | 0.4 | 4 | 0.8133 | 0.7639 | 0.7440 | 0.6754 |
| 314 | 1 | 301 | 0.4 | 4 | 0.8167 | 0.7725 | 0.7500 | 0.6685 |
| 315 | 2 | 301 | 0.4 | 4 | 0.8162 | 0.7742 | 0.7521 | 0.6569 |
| 316 | 3 | 301 | 0.4 | 4 | 0.8185 | 0.7515 | 0.7444 | 0.6740 |
| 317 | 4 | 301 | 0.4 | 4 | 0.8139 | 0.7532 | 0.7437 | 0.6581 |
| 318 | Mean | 301 | 0.4 | 4 | 0.8157 | 0.7631 | 0.7468 | 0.6666 |
| 319 | 0 | 301 | 0.4 | 5 | 0.8628 | 0.7910 | 0.7770 | 0.6860 |
| 320 | 1 | 301 | 0.4 | 5 | 0.8618 | 0.7972 | 0.7779 | 0.6751 |
| 321 | 2 | 301 | 0.4 | 5 | 0.8635 | 0.7996 | 0.7798 | 0.6675 |
| 322 | 3 | 301 | 0.4 | 5 | 0.8609 | 0.7820 | 0.7710 | 0.6799 |
| 323 | 4 | 301 | 0.4 | 5 | 0.8620 | 0.7729 | 0.7725 | 0.6685 |
| 324 | Mean | 301 | 0.4 | 5 | 0.8622 | 0.7885 | 0.7756 | 0.6754 |
| 325 | 0 | 301 | 0.5 |  | 0.9997 | 0.8541 | 0.8558 | 0.7297 |
| 326 | 1 | 301 | 0.5 |  | 0.9997 | 0.8638 | 0.8469 | 0.7249 |
| 327 | 2 | 301 | 0.5 |  | 0.9997 | 0.8467 | 0.8440 | 0.6988 |
| 328 | 3 | 301 | 0.5 |  | 0.9997 | 0.8663 | 0.8504 | 0.7280 |
| 329 | 4 | 301 | 0.5 |  | 0.9997 | 0.8279 | 0.8504 | 0.7181 |
| 330 | Mean | 301 | 0.5 |  | 0.9997 | 0.8518 | 0.8495 | 0.7199 |
| 331 | 0 | 301 | 0.5 | 1 | 0.6557 | 0.6216 | 0.6282 | 0.5557 |
| 332 | 1 | 301 | 0.5 | 1 | 0.6635 | 0.6534 | 0.6379 | 0.5380 |
| 333 | 2 | 301 | 0.5 | 1 | 0.6561 | 0.6769 | 0.6342 | 0.5605 |
| 334 | 3 | 301 | 0.5 | 1 | 0.6643 | 0.6302 | 0.6370 | 0.5568 |
| 335 | 4 | 301 | 0.5 | 1 | 0.6631 | 0.6440 | 0.6356 | 0.5899 |
| 336 | Mean | 301 | 0.5 | 1 | 0.6606 | 0.6452 | 0.6346 | 0.5602 |
| 337 | 0 | 301 | 0.5 | 2 | 0.7073 | 0.6825 | 0.6638 | 0.6135 |
| 338 | 1 | 301 | 0.5 | 2 | 0.7056 | 0.6994 | 0.6692 | 0.6157 |
| 339 | 2 | 301 | 0.5 | 2 | 0.7114 | 0.7013 | 0.6685 | 0.6106 |
| 340 | 3 | 301 | 0.5 | 2 | 0.7134 | 0.6702 | 0.6650 | 0.6252 |
| 341 | 4 | 301 | 0.5 | 2 | 0.7097 | 0.6782 | 0.6625 | 0.6289 |
| 342 | Mean | 301 | 0.5 | 2 | 0.7095 | 0.6863 | 0.6658 | 0.6188 |
| 343 | 0 | 301 | 0.5 | 3 | 0.7609 | 0.7282 | 0.7071 | 0.6638 |
| 344 | 1 | 301 | 0.5 | 3 | 0.7620 | 0.7382 | 0.7112 | 0.6438 |
| 345 | 2 | 301 | 0.5 | 3 | 0.7598 | 0.7437 | 0.7098 | 0.6376 |
| 346 | 3 | 301 | 0.5 | 3 | 0.7618 | 0.7075 | 0.7002 | 0.6460 |
| 347 | 4 | 301 | 0.5 | 3 | 0.7586 | 0.7198 | 0.6993 | 0.6628 |
| 348 | Mean | 301 | 0.5 | 3 | 0.7606 | 0.7275 | 0.7055 | 0.6508 |
| 349 | 0 | 301 | 0.5 | 4 | 0.8153 | 0.7651 | 0.7465 | 0.6804 |
| 350 | 1 | 301 | 0.5 | 4 | 0.8150 | 0.7687 | 0.7461 | 0.6589 |
| 351 | 2 | 301 | 0.5 | 4 | 0.8179 | 0.7751 | 0.7515 | 0.6538 |
| 352 | 3 | 301 | 0.5 | 4 | 0.8179 | 0.7521 | 0.7454 | 0.6587 |
| 353 | 4 | 301 | 0.5 | 4 | 0.8143 | 0.7502 | 0.7417 | 0.6606 |
| 354 | Mean | 301 | 0.5 | 4 | 0.8161 | 0.7622 | 0.7462 | 0.6625 |
| 355 | 0 | 301 | 0.5 | 5 | 0.8629 | 0.7898 | 0.7775 | 0.6910 |
| 356 | 1 | 301 | 0.5 | 5 | 0.8619 | 0.7976 | 0.7762 | 0.6674 |
| 357 | 2 | 301 | 0.5 | 5 | 0.8656 | 0.7973 | 0.7789 | 0.6667 |
| 358 | 3 | 301 | 0.5 | 5 | 0.8635 | 0.7806 | 0.7719 | 0.6705 |
| 359 | 4 | 301 | 0.5 | 5 | 0.8641 | 0.7747 | 0.7738 | 0.6696 |
| 360 | Mean | 301 | 0.5 | 5 | 0.8636 | 0.7880 | 0.7757 | 0.6730 |
| 361 | 0 | 501 | 0.1 |  | 0.9997 | 0.8691 | 0.8675 | 0.7289 |
| 362 | 1 | 501 | 0.1 |  | 0.9997 | 0.8729 | 0.8577 | 0.7298 |
| 363 | 2 | 501 | 0.1 |  | 0.9997 | 0.8633 | 0.8564 | 0.7010 |
| 364 | 3 | 501 | 0.1 |  | 0.9997 | 0.8742 | 0.8615 | 0.7277 |
| 365 | 4 | 501 | 0.1 |  | 0.9997 | 0.8421 | 0.8601 | 0.7329 |
| 366 | Mean | 501 | 0.1 |  | 0.9997 | 0.8643 | 0.8606 | 0.7240 |
| 367 | 0 | 501 | 0.1 | 1 | 0.6664 | 0.6185 | 0.6326 | 0.5291 |
| 368 | 1 | 501 | 0.1 | 1 | 0.6721 | 0.6604 | 0.6455 | 0.5277 |
| 369 | 2 | 501 | 0.1 | 1 | 0.6708 | 0.6829 | 0.6486 | 0.5396 |
| 370 | 3 | 501 | 0.1 | 1 | 0.6756 | 0.6452 | 0.6473 | 0.5193 |
| 371 | 4 | 501 | 0.1 | 1 | 0.6776 | 0.6571 | 0.6429 | 0.5742 |
| 372 | Mean | 501 | 0.1 | 1 | 0.6725 | 0.6528 | 0.6434 | 0.5380 |
| 373 | 0 | 501 | 0.1 | 2 | 0.7107 | 0.6687 | 0.6659 | 0.6255 |
| 374 | 1 | 501 | 0.1 | 2 | 0.7135 | 0.6975 | 0.6767 | 0.6289 |
| 375 | 2 | 501 | 0.1 | 2 | 0.7178 | 0.7124 | 0.6785 | 0.6502 |
| 376 | 3 | 501 | 0.1 | 2 | 0.7198 | 0.6838 | 0.6753 | 0.6189 |
| 377 | 4 | 501 | 0.1 | 2 | 0.7141 | 0.6891 | 0.6659 | 0.6392 |
| 378 | Mean | 501 | 0.1 | 2 | 0.7152 | 0.6903 | 0.6725 | 0.6325 |
| 379 | 0 | 501 | 0.1 | 3 | 0.7554 | 0.7102 | 0.6993 | 0.6607 |
| 380 | 1 | 501 | 0.1 | 3 | 0.7571 | 0.7272 | 0.7044 | 0.6627 |
| 381 | 2 | 501 | 0.1 | 3 | 0.7591 | 0.7408 | 0.7080 | 0.6692 |
| 382 | 3 | 501 | 0.1 | 3 | 0.7629 | 0.7163 | 0.7077 | 0.6667 |
| 383 | 4 | 501 | 0.1 | 3 | 0.7580 | 0.7187 | 0.7008 | 0.6556 |
| 384 | Mean | 501 | 0.1 | 3 | 0.7585 | 0.7226 | 0.7040 | 0.6630 |
| 385 | 0 | 501 | 0.1 | 4 | 0.8009 | 0.7438 | 0.7326 | 0.6832 |
| 386 | 1 | 501 | 0.1 | 4 | 0.8046 | 0.7628 | 0.7371 | 0.6727 |
| 387 | 2 | 501 | 0.1 | 4 | 0.8066 | 0.7747 | 0.7416 | 0.6775 |
| 388 | 3 | 501 | 0.1 | 4 | 0.8102 | 0.7578 | 0.7425 | 0.6903 |
| 389 | 4 | 501 | 0.1 | 4 | 0.8094 | 0.7475 | 0.7374 | 0.6778 |
| 390 | Mean | 501 | 0.1 | 4 | 0.8063 | 0.7573 | 0.7382 | 0.6803 |
| 391 | 0 | 501 | 0.1 | 5 | 0.8493 | 0.7776 | 0.7674 | 0.6966 |
| 392 | 1 | 501 | 0.1 | 5 | 0.8503 | 0.7915 | 0.7683 | 0.6776 |
| 393 | 2 | 501 | 0.1 | 5 | 0.8533 | 0.7956 | 0.7747 | 0.6768 |
| 394 | 3 | 501 | 0.1 | 5 | 0.8564 | 0.7891 | 0.7730 | 0.6904 |
| 395 | 4 | 501 | 0.1 | 5 | 0.8529 | 0.7699 | 0.7679 | 0.6828 |
| 396 | Mean | 501 | 0.1 | 5 | 0.8525 | 0.7847 | 0.7703 | 0.6849 |
| 397 | 0 | 501 | 0.2 |  | 0.9997 | 0.8623 | 0.8623 | 0.7293 |
| 398 | 1 | 501 | 0.2 |  | 0.9997 | 0.8696 | 0.8538 | 0.7241 |
| 399 | 2 | 501 | 0.2 |  | 0.9997 | 0.8568 | 0.8538 | 0.6993 |
| 400 | 3 | 501 | 0.2 |  | 0.9997 | 0.8692 | 0.8581 | 0.7212 |
| 401 | 4 | 501 | 0.2 |  | 0.9997 | 0.8345 | 0.8554 | 0.7250 |
| 402 | Mean | 501 | 0.2 |  | 0.9997 | 0.8585 | 0.8567 | 0.7198 |
| 403 | 0 | 501 | 0.2 | 1 | 0.6589 | 0.6183 | 0.6294 | 0.5495 |
| 404 | 1 | 501 | 0.2 | 1 | 0.6613 | 0.6474 | 0.6367 | 0.5356 |
| 405 | 2 | 501 | 0.2 | 1 | 0.6648 | 0.6837 | 0.6470 | 0.5311 |
| 406 | 3 | 501 | 0.2 | 1 | 0.6646 | 0.6296 | 0.6351 | 0.5323 |
| 407 | 4 | 501 | 0.2 | 1 | 0.6651 | 0.6486 | 0.6394 | 0.5562 |
| 408 | Mean | 501 | 0.2 | 1 | 0.6629 | 0.6455 | 0.6375 | 0.5409 |
| 409 | 0 | 501 | 0.2 | 2 | 0.7088 | 0.6752 | 0.6630 | 0.6314 |
| 410 | 1 | 501 | 0.2 | 2 | 0.7069 | 0.6983 | 0.6678 | 0.6243 |
| 411 | 2 | 501 | 0.2 | 2 | 0.7124 | 0.7120 | 0.6732 | 0.6259 |
| 412 | 3 | 501 | 0.2 | 2 | 0.7184 | 0.6814 | 0.6714 | 0.6260 |
| 413 | 4 | 501 | 0.2 | 2 | 0.7098 | 0.6816 | 0.6602 | 0.6333 |
| 414 | Mean | 501 | 0.2 | 2 | 0.7113 | 0.6897 | 0.6671 | 0.6282 |
| 415 | 0 | 501 | 0.2 | 3 | 0.7565 | 0.7187 | 0.7015 | 0.6719 |
| 416 | 1 | 501 | 0.2 | 3 | 0.7588 | 0.7348 | 0.7080 | 0.6667 |
| 417 | 2 | 501 | 0.2 | 3 | 0.7604 | 0.7422 | 0.7124 | 0.6549 |
| 418 | 3 | 501 | 0.2 | 3 | 0.7665 | 0.7173 | 0.7114 | 0.6662 |
| 419 | 4 | 501 | 0.2 | 3 | 0.7598 | 0.7167 | 0.6992 | 0.6660 |
| 420 | Mean | 501 | 0.2 | 3 | 0.7604 | 0.7259 | 0.7065 | 0.6651 |
| 421 | 0 | 501 | 0.2 | 4 | 0.8087 | 0.7547 | 0.7409 | 0.6814 |
| 422 | 1 | 501 | 0.2 | 4 | 0.8106 | 0.7689 | 0.7448 | 0.6711 |
| 423 | 2 | 501 | 0.2 | 4 | 0.8126 | 0.7782 | 0.7495 | 0.6765 |
| 424 | 3 | 501 | 0.2 | 4 | 0.8155 | 0.7548 | 0.7466 | 0.6856 |
| 425 | 4 | 501 | 0.2 | 4 | 0.8114 | 0.7495 | 0.7398 | 0.6747 |
| 426 | Mean | 501 | 0.2 | 4 | 0.8118 | 0.7612 | 0.7443 | 0.6779 |
| 427 | 0 | 501 | 0.2 | 5 | 0.8582 | 0.7831 | 0.7744 | 0.6918 |
| 428 | 1 | 501 | 0.2 | 5 | 0.8568 | 0.7974 | 0.7728 | 0.6809 |
| 429 | 2 | 501 | 0.2 | 5 | 0.8584 | 0.8000 | 0.7777 | 0.6784 |
| 430 | 3 | 501 | 0.2 | 5 | 0.8622 | 0.7859 | 0.7767 | 0.6908 |
| 431 | 4 | 501 | 0.2 | 5 | 0.8599 | 0.7718 | 0.7733 | 0.6742 |
| 432 | Mean | 501 | 0.2 | 5 | 0.8591 | 0.7877 | 0.7750 | 0.6832 |
| 433 | 0 | 501 | 0.3 |  | 0.9997 | 0.8608 | 0.8595 | 0.7305 |
| 434 | 1 | 501 | 0.3 |  | 0.9997 | 0.8680 | 0.8492 | 0.7215 |
| 435 | 2 | 501 | 0.3 |  | 0.9997 | 0.8524 | 0.8485 | 0.6977 |
| 436 | 3 | 501 | 0.3 |  | 0.9997 | 0.8675 | 0.8545 | 0.7308 |
| 437 | 4 | 501 | 0.3 |  | 0.9997 | 0.8323 | 0.8524 | 0.7247 |
| 438 | Mean | 501 | 0.3 |  | 0.9997 | 0.8562 | 0.8528 | 0.7211 |
| 439 | 0 | 501 | 0.3 | 1 | 0.6567 | 0.6185 | 0.6279 | 0.5494 |
| 440 | 1 | 501 | 0.3 | 1 | 0.6562 | 0.6505 | 0.6346 | 0.5489 |
| 441 | 2 | 501 | 0.3 | 1 | 0.6643 | 0.6802 | 0.6429 | 0.5400 |
| 442 | 3 | 501 | 0.3 | 1 | 0.6615 | 0.6294 | 0.6354 | 0.5440 |
| 443 | 4 | 501 | 0.3 | 1 | 0.6613 | 0.6443 | 0.6348 | 0.5713 |
| 444 | Mean | 501 | 0.3 | 1 | 0.6600 | 0.6446 | 0.6351 | 0.5507 |
| 445 | 0 | 501 | 0.3 | 2 | 0.7077 | 0.6777 | 0.6635 | 0.6298 |
| 446 | 1 | 501 | 0.3 | 2 | 0.7090 | 0.7022 | 0.6725 | 0.6208 |
| 447 | 2 | 501 | 0.3 | 2 | 0.7108 | 0.7090 | 0.6701 | 0.6235 |
| 448 | 3 | 501 | 0.3 | 2 | 0.7166 | 0.6770 | 0.6697 | 0.6210 |
| 449 | 4 | 501 | 0.3 | 2 | 0.7080 | 0.6785 | 0.6625 | 0.6379 |
| 450 | Mean | 501 | 0.3 | 2 | 0.7104 | 0.6889 | 0.6677 | 0.6266 |
| 451 | 0 | 501 | 0.3 | 3 | 0.7585 | 0.7212 | 0.7049 | 0.6640 |
| 452 | 1 | 501 | 0.3 | 3 | 0.7602 | 0.7378 | 0.7079 | 0.6613 |
| 453 | 2 | 501 | 0.3 | 3 | 0.7586 | 0.7389 | 0.7100 | 0.6525 |
| 454 | 3 | 501 | 0.3 | 3 | 0.7651 | 0.7147 | 0.7084 | 0.6660 |
| 455 | 4 | 501 | 0.3 | 3 | 0.7600 | 0.7158 | 0.6982 | 0.6636 |
| 456 | Mean | 501 | 0.3 | 3 | 0.7605 | 0.7257 | 0.7059 | 0.6615 |
| 457 | 0 | 501 | 0.3 | 4 | 0.8138 | 0.7617 | 0.7444 | 0.6757 |
| 458 | 1 | 501 | 0.3 | 4 | 0.8140 | 0.7710 | 0.7472 | 0.6754 |
| 459 | 2 | 501 | 0.3 | 4 | 0.8168 | 0.7780 | 0.7525 | 0.6626 |
| 460 | 3 | 501 | 0.3 | 4 | 0.8180 | 0.7575 | 0.7467 | 0.6783 |
| 461 | 4 | 501 | 0.3 | 4 | 0.8134 | 0.7483 | 0.7405 | 0.6689 |
| 462 | Mean | 501 | 0.3 | 4 | 0.8152 | 0.7633 | 0.7463 | 0.6722 |
| 463 | 0 | 501 | 0.3 | 5 | 0.8609 | 0.7882 | 0.7767 | 0.6892 |
| 464 | 1 | 501 | 0.3 | 5 | 0.8598 | 0.7961 | 0.7736 | 0.6745 |
| 465 | 2 | 501 | 0.3 | 5 | 0.8608 | 0.7993 | 0.7781 | 0.6727 |
| 466 | 3 | 501 | 0.3 | 5 | 0.8650 | 0.7863 | 0.7759 | 0.6926 |
| 467 | 4 | 501 | 0.3 | 5 | 0.8626 | 0.7735 | 0.7735 | 0.6765 |
| 468 | Mean | 501 | 0.3 | 5 | 0.8618 | 0.7887 | 0.7756 | 0.6811 |
| 469 | 0 | 501 | 0.4 |  | 0.9997 | 0.8607 | 0.8587 | 0.7329 |
| 470 | 1 | 501 | 0.4 |  | 0.9997 | 0.8653 | 0.8491 | 0.7253 |
| 471 | 2 | 501 | 0.4 |  | 0.9997 | 0.8496 | 0.8482 | 0.6985 |
| 472 | 3 | 501 | 0.4 |  | 0.9997 | 0.8644 | 0.8517 | 0.7265 |
| 473 | 4 | 501 | 0.4 |  | 0.9997 | 0.8320 | 0.8521 | 0.7273 |
| 474 | Mean | 501 | 0.4 |  | 0.9997 | 0.8544 | 0.8520 | 0.7221 |
| 475 | 0 | 501 | 0.4 | 1 | 0.6576 | 0.6172 | 0.6251 | 0.5540 |
| 476 | 1 | 501 | 0.4 | 1 | 0.6609 | 0.6514 | 0.6349 | 0.5548 |
| 477 | 2 | 501 | 0.4 | 1 | 0.6612 | 0.6776 | 0.6391 | 0.5520 |
| 478 | 3 | 501 | 0.4 | 1 | 0.6651 | 0.6301 | 0.6374 | 0.5455 |
| 479 | 4 | 501 | 0.4 | 1 | 0.6596 | 0.6443 | 0.6351 | 0.5782 |
| 480 | Mean | 501 | 0.4 | 1 | 0.6609 | 0.6441 | 0.6343 | 0.5569 |
| 481 | 0 | 501 | 0.4 | 2 | 0.7078 | 0.6794 | 0.6639 | 0.6201 |
| 482 | 1 | 501 | 0.4 | 2 | 0.7061 | 0.7000 | 0.6704 | 0.5998 |
| 483 | 2 | 501 | 0.4 | 2 | 0.7096 | 0.7029 | 0.6656 | 0.6207 |
| 484 | 3 | 501 | 0.4 | 2 | 0.7152 | 0.6733 | 0.6684 | 0.6241 |
| 485 | 4 | 501 | 0.4 | 2 | 0.7082 | 0.6792 | 0.6611 | 0.6279 |
| 486 | Mean | 501 | 0.4 | 2 | 0.7094 | 0.6869 | 0.6659 | 0.6185 |
| 487 | 0 | 501 | 0.4 | 3 | 0.7572 | 0.7246 | 0.7036 | 0.6600 |
| 488 | 1 | 501 | 0.4 | 3 | 0.7641 | 0.7418 | 0.7138 | 0.6572 |
| 489 | 2 | 501 | 0.4 | 3 | 0.7580 | 0.7375 | 0.7087 | 0.6503 |
| 490 | 3 | 501 | 0.4 | 3 | 0.7664 | 0.7129 | 0.7058 | 0.6548 |
| 491 | 4 | 501 | 0.4 | 3 | 0.7592 | 0.7147 | 0.6986 | 0.6600 |
| 492 | Mean | 501 | 0.4 | 3 | 0.7610 | 0.7263 | 0.7061 | 0.6565 |
| 493 | 0 | 501 | 0.4 | 4 | 0.8151 | 0.7606 | 0.7440 | 0.6781 |
| 494 | 1 | 501 | 0.4 | 4 | 0.8140 | 0.7692 | 0.7471 | 0.6703 |
| 495 | 2 | 501 | 0.4 | 4 | 0.8159 | 0.7765 | 0.7518 | 0.6650 |
| 496 | 3 | 501 | 0.4 | 4 | 0.8188 | 0.7547 | 0.7474 | 0.6716 |
| 497 | 4 | 501 | 0.4 | 4 | 0.8137 | 0.7498 | 0.7409 | 0.6681 |
| 498 | Mean | 501 | 0.4 | 4 | 0.8155 | 0.7622 | 0.7462 | 0.6706 |
| 499 | 0 | 501 | 0.4 | 5 | 0.8641 | 0.7901 | 0.7797 | 0.6886 |
| 500 | 1 | 501 | 0.4 | 5 | 0.8612 | 0.7984 | 0.7763 | 0.6743 |
| 501 | 2 | 501 | 0.4 | 5 | 0.8610 | 0.7978 | 0.7760 | 0.6695 |
| 502 | 3 | 501 | 0.4 | 5 | 0.8636 | 0.7796 | 0.7725 | 0.6862 |
| 503 | 4 | 501 | 0.4 | 5 | 0.8642 | 0.7741 | 0.7739 | 0.6663 |
| 504 | Mean | 501 | 0.4 | 5 | 0.8628 | 0.7880 | 0.7757 | 0.6770 |
| 505 | 0 | 501 | 0.5 |  | 0.9997 | 0.8600 | 0.8557 | 0.7301 |
| 506 | 1 | 501 | 0.5 |  | 0.9997 | 0.8615 | 0.8476 | 0.7248 |
| 507 | 2 | 501 | 0.5 |  | 0.9997 | 0.8465 | 0.8448 | 0.7013 |
| 508 | 3 | 501 | 0.5 |  | 0.9997 | 0.8628 | 0.8512 | 0.7228 |
| 509 | 4 | 501 | 0.5 |  | 0.9997 | 0.8302 | 0.8503 | 0.7240 |
| 510 | Mean | 501 | 0.5 |  | 0.9997 | 0.8522 | 0.8499 | 0.7206 |
| 511 | 0 | 501 | 0.5 | 1 | 0.6612 | 0.6233 | 0.6280 | 0.5598 |
| 512 | 1 | 501 | 0.5 | 1 | 0.6596 | 0.6512 | 0.6368 | 0.5604 |
| 513 | 2 | 501 | 0.5 | 1 | 0.6645 | 0.6799 | 0.6456 | 0.5529 |
| 514 | 3 | 501 | 0.5 | 1 | 0.6642 | 0.6284 | 0.6348 | 0.5510 |
| 515 | 4 | 501 | 0.5 | 1 | 0.6648 | 0.6497 | 0.6393 | 0.5811 |
| 516 | Mean | 501 | 0.5 | 1 | 0.6629 | 0.6465 | 0.6369 | 0.5611 |
| 517 | 0 | 501 | 0.5 | 2 | 0.7064 | 0.6789 | 0.6632 | 0.6239 |
| 518 | 1 | 501 | 0.5 | 2 | 0.7077 | 0.7040 | 0.6703 | 0.6117 |
| 519 | 2 | 501 | 0.5 | 2 | 0.7061 | 0.6984 | 0.6637 | 0.5934 |
| 520 | 3 | 501 | 0.5 | 2 | 0.7146 | 0.6712 | 0.6660 | 0.6146 |
| 521 | 4 | 501 | 0.5 | 2 | 0.7053 | 0.6784 | 0.6580 | 0.6443 |
| 522 | Mean | 501 | 0.5 | 2 | 0.7080 | 0.6862 | 0.6642 | 0.6176 |
| 523 | 0 | 501 | 0.5 | 3 | 0.7615 | 0.7283 | 0.7069 | 0.6670 |
| 524 | 1 | 501 | 0.5 | 3 | 0.7637 | 0.7404 | 0.7115 | 0.6534 |
| 525 | 2 | 501 | 0.5 | 3 | 0.7593 | 0.7394 | 0.7068 | 0.6405 |
| 526 | 3 | 501 | 0.5 | 3 | 0.7623 | 0.7069 | 0.7020 | 0.6438 |
| 527 | 4 | 501 | 0.5 | 3 | 0.7594 | 0.7184 | 0.6991 | 0.6561 |
| 528 | Mean | 501 | 0.5 | 3 | 0.7613 | 0.7267 | 0.7053 | 0.6521 |
| 529 | 0 | 501 | 0.5 | 4 | 0.8160 | 0.7639 | 0.7469 | 0.6785 |
| 530 | 1 | 501 | 0.5 | 4 | 0.8154 | 0.7713 | 0.7478 | 0.6584 |
| 531 | 2 | 501 | 0.5 | 4 | 0.8160 | 0.7796 | 0.7515 | 0.6590 |
| 532 | 3 | 501 | 0.5 | 4 | 0.8159 | 0.7492 | 0.7439 | 0.6693 |
| 533 | 4 | 501 | 0.5 | 4 | 0.8148 | 0.7513 | 0.7416 | 0.6603 |
| 534 | Mean | 501 | 0.5 | 4 | 0.8156 | 0.7631 | 0.7463 | 0.6651 |
| 535 | 0 | 501 | 0.5 | 5 | 0.8630 | 0.7906 | 0.7784 | 0.6872 |
| 536 | 1 | 501 | 0.5 | 5 | 0.8652 | 0.7979 | 0.7790 | 0.6728 |
| 537 | 2 | 501 | 0.5 | 5 | 0.8632 | 0.7960 | 0.7768 | 0.6654 |
| 538 | 3 | 501 | 0.5 | 5 | 0.8630 | 0.7780 | 0.7730 | 0.6841 |
| 539 | 4 | 501 | 0.5 | 5 | 0.8642 | 0.7759 | 0.7754 | 0.6719 |
| 540 | Mean | 501 | 0.5 | 5 | 0.8637 | 0.7877 | 0.7765 | 0.6763 |
| 541 | 0 | 1001 | 0.1 |  | 0.9997 | 0.8664 | 0.8665 | 0.7334 |
| 542 | 1 | 1001 | 0.1 |  | 0.9997 | 0.8731 | 0.8593 | 0.7281 |
| 543 | 2 | 1001 | 0.1 |  | 0.9997 | 0.8621 | 0.8567 | 0.6973 |
| 544 | 3 | 1001 | 0.1 |  | 0.9997 | 0.8740 | 0.8630 | 0.7267 |
| 545 | 4 | 1001 | 0.1 |  | 0.9997 | 0.8417 | 0.8610 | 0.7237 |
| 546 | Mean | 1001 | 0.1 |  | 0.9997 | 0.8635 | 0.8613 | 0.7219 |
| 547 | 0 | 1001 | 0.1 | 1 | 0.6690 | 0.6258 | 0.6375 | 0.5302 |
| 548 | 1 | 1001 | 0.1 | 1 | 0.6714 | 0.6608 | 0.6465 | 0.5274 |
| 549 | 2 | 1001 | 0.1 | 1 | 0.6720 | 0.6868 | 0.6501 | 0.5344 |
| 550 | 3 | 1001 | 0.1 | 1 | 0.6728 | 0.6425 | 0.6450 | 0.5217 |
| 551 | 4 | 1001 | 0.1 | 1 | 0.6794 | 0.6566 | 0.6447 | 0.5713 |
| 552 | Mean | 1001 | 0.1 | 1 | 0.6729 | 0.6545 | 0.6447 | 0.5370 |
| 553 | 0 | 1001 | 0.1 | 2 | 0.7109 | 0.6710 | 0.6654 | 0.6194 |
| 554 | 1 | 1001 | 0.1 | 2 | 0.7173 | 0.6989 | 0.6784 | 0.6307 |
| 555 | 2 | 1001 | 0.1 | 2 | 0.7157 | 0.7143 | 0.6775 | 0.6434 |
| 556 | 3 | 1001 | 0.1 | 2 | 0.7196 | 0.6856 | 0.6759 | 0.6267 |
| 557 | 4 | 1001 | 0.1 | 2 | 0.7152 | 0.6876 | 0.6682 | 0.6333 |
| 558 | Mean | 1001 | 0.1 | 2 | 0.7157 | 0.6915 | 0.6731 | 0.6307 |
| 559 | 0 | 1001 | 0.1 | 3 | 0.7534 | 0.7077 | 0.6983 | 0.6616 |
| 560 | 1 | 1001 | 0.1 | 3 | 0.7547 | 0.7270 | 0.7024 | 0.6691 |
| 561 | 2 | 1001 | 0.1 | 3 | 0.7582 | 0.7434 | 0.7104 | 0.6660 |
| 562 | 3 | 1001 | 0.1 | 3 | 0.7606 | 0.7162 | 0.7053 | 0.6727 |
| 563 | 4 | 1001 | 0.1 | 3 | 0.7596 | 0.7196 | 0.7012 | 0.6621 |
| 564 | Mean | 1001 | 0.1 | 3 | 0.7573 | 0.7228 | 0.7035 | 0.6663 |
| 565 | 0 | 1001 | 0.1 | 4 | 0.8027 | 0.7459 | 0.7341 | 0.6823 |
| 566 | 1 | 1001 | 0.1 | 4 | 0.8049 | 0.7628 | 0.7394 | 0.6740 |
| 567 | 2 | 1001 | 0.1 | 4 | 0.8051 | 0.7679 | 0.7422 | 0.6721 |
| 568 | 3 | 1001 | 0.1 | 4 | 0.8087 | 0.7557 | 0.7405 | 0.6877 |
| 569 | 4 | 1001 | 0.1 | 4 | 0.8065 | 0.7472 | 0.7354 | 0.6788 |
| 570 | Mean | 1001 | 0.1 | 4 | 0.8056 | 0.7559 | 0.7383 | 0.6790 |
| 571 | 0 | 1001 | 0.1 | 5 | 0.8497 | 0.7767 | 0.7665 | 0.6945 |
| 572 | 1 | 1001 | 0.1 | 5 | 0.8494 | 0.7924 | 0.7670 | 0.6786 |
| 573 | 2 | 1001 | 0.1 | 5 | 0.8530 | 0.7963 | 0.7739 | 0.6727 |
| 574 | 3 | 1001 | 0.1 | 5 | 0.8557 | 0.7873 | 0.7728 | 0.6882 |
| 575 | 4 | 1001 | 0.1 | 5 | 0.8508 | 0.7713 | 0.7668 | 0.6788 |
| 576 | Mean | 1001 | 0.1 | 5 | 0.8517 | 0.7848 | 0.7694 | 0.6825 |
| 577 | 0 | 1001 | 0.2 |  | 0.9997 | 0.8638 | 0.8624 | 0.7331 |
| 578 | 1 | 1001 | 0.2 |  | 0.9997 | 0.8699 | 0.8540 | 0.7249 |
| 579 | 2 | 1001 | 0.2 |  | 0.9997 | 0.8561 | 0.8520 | 0.7004 |
| 580 | 3 | 1001 | 0.2 |  | 0.9997 | 0.8719 | 0.8578 | 0.7239 |
| 581 | 4 | 1001 | 0.2 |  | 0.9997 | 0.8353 | 0.8569 | 0.7237 |
| 582 | Mean | 1001 | 0.2 |  | 0.9997 | 0.8594 | 0.8566 | 0.7212 |
| 583 | 0 | 1001 | 0.2 | 1 | 0.6533 | 0.6122 | 0.6238 | 0.5380 |
| 584 | 1 | 1001 | 0.2 | 1 | 0.6605 | 0.6494 | 0.6357 | 0.5306 |
| 585 | 2 | 1001 | 0.2 | 1 | 0.6645 | 0.6822 | 0.6456 | 0.5294 |
| 586 | 3 | 1001 | 0.2 | 1 | 0.6652 | 0.6316 | 0.6372 | 0.5287 |
| 587 | 4 | 1001 | 0.2 | 1 | 0.6679 | 0.6512 | 0.6384 | 0.5576 |
| 588 | Mean | 1001 | 0.2 | 1 | 0.6623 | 0.6453 | 0.6362 | 0.5369 |
| 589 | 0 | 1001 | 0.2 | 2 | 0.7088 | 0.6766 | 0.6642 | 0.6177 |
| 590 | 1 | 1001 | 0.2 | 2 | 0.7100 | 0.6999 | 0.6696 | 0.6239 |
| 591 | 2 | 1001 | 0.2 | 2 | 0.7118 | 0.7060 | 0.6696 | 0.6376 |
| 592 | 3 | 1001 | 0.2 | 2 | 0.7163 | 0.6782 | 0.6720 | 0.6226 |
| 593 | 4 | 1001 | 0.2 | 2 | 0.7117 | 0.6835 | 0.6640 | 0.6302 |
| 594 | Mean | 1001 | 0.2 | 2 | 0.7117 | 0.6888 | 0.6679 | 0.6264 |
| 595 | 0 | 1001 | 0.2 | 3 | 0.7560 | 0.7153 | 0.7000 | 0.6637 |
| 596 | 1 | 1001 | 0.2 | 3 | 0.7607 | 0.7347 | 0.7097 | 0.6673 |
| 597 | 2 | 1001 | 0.2 | 3 | 0.7597 | 0.7425 | 0.7121 | 0.6687 |
| 598 | 3 | 1001 | 0.2 | 3 | 0.7651 | 0.7161 | 0.7078 | 0.6680 |
| 599 | 4 | 1001 | 0.2 | 3 | 0.7590 | 0.7188 | 0.7002 | 0.6679 |
| 600 | Mean | 1001 | 0.2 | 3 | 0.7601 | 0.7255 | 0.7059 | 0.6671 |
| 601 | 0 | 1001 | 0.2 | 4 | 0.8100 | 0.7545 | 0.7402 | 0.6854 |
| 602 | 1 | 1001 | 0.2 | 4 | 0.8113 | 0.7709 | 0.7464 | 0.6699 |
| 603 | 2 | 1001 | 0.2 | 4 | 0.8131 | 0.7771 | 0.7500 | 0.6718 |
| 604 | 3 | 1001 | 0.2 | 4 | 0.8155 | 0.7558 | 0.7472 | 0.6849 |
| 605 | 4 | 1001 | 0.2 | 4 | 0.8109 | 0.7499 | 0.7393 | 0.6757 |
| 606 | Mean | 1001 | 0.2 | 4 | 0.8122 | 0.7617 | 0.7446 | 0.6776 |
| 607 | 0 | 1001 | 0.2 | 5 | 0.8570 | 0.7837 | 0.7727 | 0.6914 |
| 608 | 1 | 1001 | 0.2 | 5 | 0.8568 | 0.7951 | 0.7722 | 0.6820 |
| 609 | 2 | 1001 | 0.2 | 5 | 0.8597 | 0.7997 | 0.7788 | 0.6740 |
| 610 | 3 | 1001 | 0.2 | 5 | 0.8619 | 0.7868 | 0.7760 | 0.6927 |
| 611 | 4 | 1001 | 0.2 | 5 | 0.8607 | 0.7760 | 0.7746 | 0.6753 |
| 612 | Mean | 1001 | 0.2 | 5 | 0.8592 | 0.7883 | 0.7749 | 0.6831 |
| 613 | 0 | 1001 | 0.3 |  | 0.9997 | 0.8617 | 0.8602 | 0.7327 |
| 614 | 1 | 1001 | 0.3 |  | 0.9997 | 0.8663 | 0.8511 | 0.7255 |
| 615 | 2 | 1001 | 0.3 |  | 0.9997 | 0.8519 | 0.8493 | 0.7007 |
| 616 | 3 | 1001 | 0.3 |  | 0.9997 | 0.8688 | 0.8555 | 0.7213 |
| 617 | 4 | 1001 | 0.3 |  | 0.9997 | 0.8330 | 0.8532 | 0.7249 |
| 618 | Mean | 1001 | 0.3 |  | 0.9997 | 0.8563 | 0.8538 | 0.7210 |
| 619 | 0 | 1001 | 0.3 | 1 | 0.6581 | 0.6210 | 0.6303 | 0.5508 |
| 620 | 1 | 1001 | 0.3 | 1 | 0.6609 | 0.6505 | 0.6362 | 0.5426 |
| 621 | 2 | 1001 | 0.3 | 1 | 0.6646 | 0.6829 | 0.6444 | 0.5352 |
| 622 | 3 | 1001 | 0.3 | 1 | 0.6618 | 0.6285 | 0.6339 | 0.5379 |
| 623 | 4 | 1001 | 0.3 | 1 | 0.6679 | 0.6490 | 0.6417 | 0.5698 |
| 624 | Mean | 1001 | 0.3 | 1 | 0.6627 | 0.6464 | 0.6373 | 0.5473 |
| 625 | 0 | 1001 | 0.3 | 2 | 0.7066 | 0.6736 | 0.6634 | 0.6182 |
| 626 | 1 | 1001 | 0.3 | 2 | 0.7076 | 0.7011 | 0.6691 | 0.6098 |
| 627 | 2 | 1001 | 0.3 | 2 | 0.7108 | 0.7075 | 0.6685 | 0.6279 |
| 628 | 3 | 1001 | 0.3 | 2 | 0.7146 | 0.6752 | 0.6688 | 0.6240 |
| 629 | 4 | 1001 | 0.3 | 2 | 0.7091 | 0.6802 | 0.6624 | 0.6268 |
| 630 | Mean | 1001 | 0.3 | 2 | 0.7097 | 0.6875 | 0.6664 | 0.6213 |
| 631 | 0 | 1001 | 0.3 | 3 | 0.7584 | 0.7232 | 0.7039 | 0.6663 |
| 632 | 1 | 1001 | 0.3 | 3 | 0.7623 | 0.7383 | 0.7111 | 0.6672 |
| 633 | 2 | 1001 | 0.3 | 3 | 0.7603 | 0.7420 | 0.7118 | 0.6590 |
| 634 | 3 | 1001 | 0.3 | 3 | 0.7664 | 0.7165 | 0.7093 | 0.6600 |
| 635 | 4 | 1001 | 0.3 | 3 | 0.7611 | 0.7190 | 0.7020 | 0.6616 |
| 636 | Mean | 1001 | 0.3 | 3 | 0.7617 | 0.7278 | 0.7076 | 0.6628 |
| 637 | 0 | 1001 | 0.3 | 4 | 0.8134 | 0.7606 | 0.7431 | 0.6818 |
| 638 | 1 | 1001 | 0.3 | 4 | 0.8134 | 0.7720 | 0.7479 | 0.6739 |
| 639 | 2 | 1001 | 0.3 | 4 | 0.8160 | 0.7774 | 0.7528 | 0.6670 |
| 640 | 3 | 1001 | 0.3 | 4 | 0.8177 | 0.7551 | 0.7473 | 0.6756 |
| 641 | 4 | 1001 | 0.3 | 4 | 0.8126 | 0.7503 | 0.7403 | 0.6731 |
| 642 | Mean | 1001 | 0.3 | 4 | 0.8146 | 0.7631 | 0.7463 | 0.6743 |
| 643 | 0 | 1001 | 0.3 | 5 | 0.8615 | 0.7895 | 0.7773 | 0.6888 |
| 644 | 1 | 1001 | 0.3 | 5 | 0.8607 | 0.7985 | 0.7772 | 0.6756 |
| 645 | 2 | 1001 | 0.3 | 5 | 0.8613 | 0.7993 | 0.7792 | 0.6702 |
| 646 | 3 | 1001 | 0.3 | 5 | 0.8650 | 0.7874 | 0.7774 | 0.6922 |
| 647 | 4 | 1001 | 0.3 | 5 | 0.8621 | 0.7762 | 0.7738 | 0.6737 |
| 648 | Mean | 1001 | 0.3 | 5 | 0.8621 | 0.7902 | 0.7770 | 0.6801 |
| 649 | 0 | 1001 | 0.4 |  | 0.9997 | 0.8612 | 0.8595 | 0.7300 |
| 650 | 1 | 1001 | 0.4 |  | 0.9997 | 0.8652 | 0.8494 | 0.7270 |
| 651 | 2 | 1001 | 0.4 |  | 0.9997 | 0.8498 | 0.8470 | 0.6981 |
| 652 | 3 | 1001 | 0.4 |  | 0.9997 | 0.8650 | 0.8529 | 0.7247 |
| 653 | 4 | 1001 | 0.4 |  | 0.9997 | 0.8344 | 0.8532 | 0.7261 |
| 654 | Mean | 1001 | 0.4 |  | 0.9997 | 0.8551 | 0.8524 | 0.7212 |
| 655 | 0 | 1001 | 0.4 | 1 | 0.6591 | 0.6220 | 0.6307 | 0.5523 |
| 656 | 1 | 1001 | 0.4 | 1 | 0.6599 | 0.6505 | 0.6385 | 0.5336 |
| 657 | 2 | 1001 | 0.4 | 1 | 0.6609 | 0.6829 | 0.6419 | 0.5519 |
| 658 | 3 | 1001 | 0.4 | 1 | 0.6651 | 0.6322 | 0.6377 | 0.5358 |
| 659 | 4 | 1001 | 0.4 | 1 | 0.6648 | 0.6449 | 0.6369 | 0.5843 |
| 660 | Mean | 1001 | 0.4 | 1 | 0.6619 | 0.6465 | 0.6371 | 0.5516 |
| 661 | 0 | 1001 | 0.4 | 2 | 0.7065 | 0.6787 | 0.6633 | 0.6138 |
| 662 | 1 | 1001 | 0.4 | 2 | 0.7081 | 0.7005 | 0.6708 | 0.6126 |
| 663 | 2 | 1001 | 0.4 | 2 | 0.7092 | 0.7034 | 0.6642 | 0.6134 |
| 664 | 3 | 1001 | 0.4 | 2 | 0.7162 | 0.6747 | 0.6708 | 0.6257 |
| 665 | 4 | 1001 | 0.4 | 2 | 0.7095 | 0.6812 | 0.6632 | 0.6302 |
| 666 | Mean | 1001 | 0.4 | 2 | 0.7099 | 0.6877 | 0.6665 | 0.6191 |
| 667 | 0 | 1001 | 0.4 | 3 | 0.7582 | 0.7246 | 0.7021 | 0.6612 |
| 668 | 1 | 1001 | 0.4 | 3 | 0.7633 | 0.7389 | 0.7119 | 0.6581 |
| 669 | 2 | 1001 | 0.4 | 3 | 0.7604 | 0.7400 | 0.7098 | 0.6535 |
| 670 | 3 | 1001 | 0.4 | 3 | 0.7664 | 0.7142 | 0.7085 | 0.6639 |
| 671 | 4 | 1001 | 0.4 | 3 | 0.7599 | 0.7181 | 0.6994 | 0.6575 |
| 672 | Mean | 1001 | 0.4 | 3 | 0.7616 | 0.7272 | 0.7063 | 0.6588 |
| 673 | 0 | 1001 | 0.4 | 4 | 0.8161 | 0.7648 | 0.7460 | 0.6783 |
| 674 | 1 | 1001 | 0.4 | 4 | 0.8155 | 0.7705 | 0.7479 | 0.6724 |
| 675 | 2 | 1001 | 0.4 | 4 | 0.8171 | 0.7761 | 0.7530 | 0.6674 |
| 676 | 3 | 1001 | 0.4 | 4 | 0.8171 | 0.7509 | 0.7439 | 0.6757 |
| 677 | 4 | 1001 | 0.4 | 4 | 0.8140 | 0.7515 | 0.7425 | 0.6672 |
| 678 | Mean | 1001 | 0.4 | 4 | 0.8160 | 0.7627 | 0.7467 | 0.6722 |
| 679 | 0 | 1001 | 0.4 | 5 | 0.8637 | 0.7916 | 0.7793 | 0.6877 |
| 680 | 1 | 1001 | 0.4 | 5 | 0.8633 | 0.7988 | 0.7780 | 0.6737 |
| 681 | 2 | 1001 | 0.4 | 5 | 0.8620 | 0.7963 | 0.7779 | 0.6709 |
| 682 | 3 | 1001 | 0.4 | 5 | 0.8652 | 0.7841 | 0.7761 | 0.6832 |
| 683 | 4 | 1001 | 0.4 | 5 | 0.8639 | 0.7761 | 0.7749 | 0.6676 |
| 684 | Mean | 1001 | 0.4 | 5 | 0.8636 | 0.7894 | 0.7772 | 0.6766 |
| 685 | 0 | 1001 | 0.5 |  | 0.9997 | 0.8590 | 0.8571 | 0.7309 |
| 686 | 1 | 1001 | 0.5 |  | 0.9997 | 0.8623 | 0.8478 | 0.7261 |
| 687 | 2 | 1001 | 0.5 |  | 0.9997 | 0.8484 | 0.8448 | 0.6956 |
| 688 | 3 | 1001 | 0.5 |  | 0.9997 | 0.8659 | 0.8515 | 0.7256 |
| 689 | 4 | 1001 | 0.5 |  | 0.9997 | 0.8317 | 0.8516 | 0.7225 |
| 690 | Mean | 1001 | 0.5 |  | 0.9997 | 0.8535 | 0.8506 | 0.7201 |
| 691 | 0 | 1001 | 0.5 | 1 | 0.6568 | 0.6207 | 0.6289 | 0.5559 |
| 692 | 1 | 1001 | 0.5 | 1 | 0.6586 | 0.6530 | 0.6353 | 0.5669 |
| 693 | 2 | 1001 | 0.5 | 1 | 0.6659 | 0.6831 | 0.6475 | 0.5433 |
| 694 | 3 | 1001 | 0.5 | 1 | 0.6658 | 0.6301 | 0.6371 | 0.5482 |
| 695 | 4 | 1001 | 0.5 | 1 | 0.6667 | 0.6499 | 0.6405 | 0.5772 |
| 696 | Mean | 1001 | 0.5 | 1 | 0.6628 | 0.6474 | 0.6379 | 0.5583 |
| 697 | 0 | 1001 | 0.5 | 2 | 0.7080 | 0.6806 | 0.6624 | 0.6204 |
| 698 | 1 | 1001 | 0.5 | 2 | 0.7073 | 0.7026 | 0.6694 | 0.6172 |
| 699 | 2 | 1001 | 0.5 | 2 | 0.7084 | 0.7021 | 0.6674 | 0.6097 |
| 700 | 3 | 1001 | 0.5 | 2 | 0.7148 | 0.6718 | 0.6683 | 0.6251 |
| 701 | 4 | 1001 | 0.5 | 2 | 0.7076 | 0.6793 | 0.6596 | 0.6342 |
| 702 | Mean | 1001 | 0.5 | 2 | 0.7092 | 0.6873 | 0.6654 | 0.6213 |
| 703 | 0 | 1001 | 0.5 | 3 | 0.7603 | 0.7294 | 0.7052 | 0.6637 |
| 704 | 1 | 1001 | 0.5 | 3 | 0.7644 | 0.7394 | 0.7123 | 0.6530 |
| 705 | 2 | 1001 | 0.5 | 3 | 0.7606 | 0.7400 | 0.7102 | 0.6394 |
| 706 | 3 | 1001 | 0.5 | 3 | 0.7654 | 0.7113 | 0.7055 | 0.6524 |
| 707 | 4 | 1001 | 0.5 | 3 | 0.7596 | 0.7199 | 0.7002 | 0.6618 |
| 708 | Mean | 1001 | 0.5 | 3 | 0.7621 | 0.7280 | 0.7067 | 0.6541 |
| 709 | 0 | 1001 | 0.5 | 4 | 0.8156 | 0.7633 | 0.7464 | 0.6816 |
| 710 | 1 | 1001 | 0.5 | 4 | 0.8165 | 0.7704 | 0.7490 | 0.6671 |
| 711 | 2 | 1001 | 0.5 | 4 | 0.8153 | 0.7760 | 0.7500 | 0.6617 |
| 712 | 3 | 1001 | 0.5 | 4 | 0.8173 | 0.7498 | 0.7448 | 0.6721 |
| 713 | 4 | 1001 | 0.5 | 4 | 0.8155 | 0.7525 | 0.7418 | 0.6646 |
| 714 | Mean | 1001 | 0.5 | 4 | 0.8160 | 0.7624 | 0.7464 | 0.6694 |
| 715 | 0 | 1001 | 0.5 | 5 | 0.8645 | 0.7917 | 0.7800 | 0.6838 |
| 716 | 1 | 1001 | 0.5 | 5 | 0.8630 | 0.7980 | 0.7776 | 0.6704 |
| 717 | 2 | 1001 | 0.5 | 5 | 0.8637 | 0.7964 | 0.7767 | 0.6659 |
| 718 | 3 | 1001 | 0.5 | 5 | 0.8633 | 0.7796 | 0.7731 | 0.6740 |
| 719 | 4 | 1001 | 0.5 | 5 | 0.8643 | 0.7777 | 0.7738 | 0.6670 |
| 720 | Mean | 1001 | 0.5 | 5 | 0.8638 | 0.7887 | 0.7763 | 0.6722 |

The first column represents the number of rows. The second column represents the number of fold that is used as test dataset. The third, fourth and firth columns represent the parameters of Random Forest, the number of random forest trees, the feature ratio of each tree, and the depth of each tree. The sixth, seventh, eighth and nineth columns represents the AUC value in different datasets.
