# Supplementary material for: PbsNRs: predict the potential binders and scaffolds for nuclear receptors
Source: Brief Bioinform. 2025 Jan 11;26(1):bbae710. doi: 10.1093/bib/bbae710 (PMC11724720; doi:10.1093/bib/bbae710)
Supplement: Supplementary_Table_6_bbae710 [file supplementary_table_6_bbae710.docx]

**Supplementary Table 7.** The performance of Deep Learning under different parameters.

| Parameter group of deep learning | Fold | Layer | Cell | PS | Valid_AUC | Test_AUC |
| --- | --- | --- | --- | --- | --- | --- |
| 1 | 0 | 1 | 100 | 0 | 0.8706 | 0.8500 |
| 2 | 1 | 1 | 100 | 0 | 0.8707 | 0.8551 |
| 3 | 2 | 1 | 100 | 0 | 0.8545 | 0.8476 |
| 4 | 3 | 1 | 100 | 0 | 0.8547 | 0.8449 |
| 5 | 4 | 1 | 100 | 0 | 0.8509 | 0.8543 |
| 6 | Mean | 1 | 100 | 0 | 0.8603 | 0.8504 |
| 7 | 0 | 1 | 100 | 0.1 | 0.8590 | 0.8492 |
| 8 | 1 | 1 | 100 | 0.1 | 0.8655 | 0.8496 |
| 9 | 2 | 1 | 100 | 0.1 | 0.8603 | 0.8510 |
| 10 | 3 | 1 | 100 | 0.1 | 0.8660 | 0.8517 |
| 11 | 4 | 1 | 100 | 0.1 | 0.8573 | 0.8510 |
| 12 | Mean | 1 | 100 | 0.1 | 0.8616 | 0.8505 |
| 13 | 0 | 1 | 100 | 0.3 | 0.8616 | 0.8522 |
| 14 | 1 | 1 | 100 | 0.3 | 0.8716 | 0.8489 |
| 15 | 2 | 1 | 100 | 0.3 | 0.8568 | 0.8466 |
| 16 | 3 | 1 | 100 | 0.3 | 0.8618 | 0.8540 |
| 17 | 4 | 1 | 100 | 0.3 | 0.8668 | 0.8530 |
| 18 | Mean | 1 | 100 | 0.3 | 0.8637 | 0.8509 |
| 19 | 0 | 1 | 100 | 0.5 | 0.8576 | 0.8475 |
| 20 | 1 | 1 | 100 | 0.5 | 0.8649 | 0.8409 |
| 21 | 2 | 1 | 100 | 0.5 | 0.8518 | 0.8424 |
| 22 | 3 | 1 | 100 | 0.5 | 0.8530 | 0.8431 |
| 23 | 4 | 1 | 100 | 0.5 | 0.8726 | 0.8458 |
| 24 | Mean | 1 | 100 | 0.5 | 0.8600 | 0.8439 |
| 25 | 0 | 1 | 300 | 0 | 0.8644 | 0.8529 |
| 26 | 1 | 1 | 300 | 0 | 0.8740 | 0.8498 |
| 27 | 2 | 1 | 300 | 0 | 0.8613 | 0.8523 |
| 28 | 3 | 1 | 300 | 0 | 0.8666 | 0.8482 |
| 29 | 4 | 1 | 300 | 0 | 0.8556 | 0.8567 |
| 30 | Mean | 1 | 300 | 0 | 0.8644 | 0.8520 |
| 31 | 0 | 1 | 300 | 0.1 | 0.8702 | 0.8590 |
| 32 | 1 | 1 | 300 | 0.1 | 0.8659 | 0.8479 |
| 33 | 2 | 1 | 300 | 0.1 | 0.8591 | 0.8524 |
| 34 | 3 | 1 | 300 | 0.1 | 0.8592 | 0.8504 |
| 35 | 4 | 1 | 300 | 0.1 | 0.8613 | 0.8559 |
| 36 | Mean | 1 | 300 | 0.1 | 0.8631 | 0.8531 |
| 37 | 0 | 1 | 300 | 0.3 | 0.8612 | 0.8531 |
| 38 | 1 | 1 | 300 | 0.3 | 0.8732 | 0.8532 |
| 39 | 2 | 1 | 300 | 0.3 | 0.8573 | 0.8484 |
| 40 | 3 | 1 | 300 | 0.3 | 0.8686 | 0.8561 |
| 41 | 4 | 1 | 300 | 0.3 | 0.8622 | 0.8586 |
| 42 | Mean | 1 | 300 | 0.3 | 0.8645 | 0.8539 |
| 43 | 0 | 1 | 300 | 0.5 | 0.8600 | 0.8473 |
| 44 | 1 | 1 | 300 | 0.5 | 0.8650 | 0.8423 |
| 45 | 2 | 1 | 300 | 0.5 | 0.8559 | 0.8430 |
| 46 | 3 | 1 | 300 | 0.5 | 0.8746 | 0.8466 |
| 47 | 4 | 1 | 300 | 0.5 | 0.8729 | 0.8573 |
| 48 | Mean | 1 | 300 | 0.5 | 0.8657 | 0.8473 |
| 49 | 0 | 1 | 500 | 0 | 0.8717 | 0.8580 |
| 50 | 1 | 1 | 500 | 0 | 0.8667 | 0.8510 |
| 51 | 2 | 1 | 500 | 0 | 0.8614 | 0.8505 |
| 52 | 3 | 1 | 500 | 0 | 0.8554 | 0.8555 |
| 53 | 4 | 1 | 500 | 0 | 0.8534 | 0.8580 |
| 54 | Mean | 1 | 500 | 0 | 0.8617 | 0.8546 |
| 55 | 0 | 1 | 500 | 0.1 | 0.8704 | 0.8591 |
| 56 | 1 | 1 | 500 | 0.1 | 0.8739 | 0.8476 |
| 57 | 2 | 1 | 500 | 0.1 | 0.8654 | 0.8464 |
| 58 | 3 | 1 | 500 | 0.1 | 0.8614 | 0.8472 |
| 59 | 4 | 1 | 500 | 0.1 | 0.8505 | 0.8609 |
| 60 | Mean | 1 | 500 | 0.1 | 0.8643 | 0.8522 |
| 61 | 0 | 1 | 500 | 0.3 | 0.8746 | 0.8563 |
| 62 | 1 | 1 | 500 | 0.3 | 0.8689 | 0.8494 |
| 63 | 2 | 1 | 500 | 0.3 | 0.8586 | 0.8450 |
| 64 | 3 | 1 | 500 | 0.3 | 0.8658 | 0.8474 |
| 65 | 4 | 1 | 500 | 0.3 | 0.8632 | 0.8568 |
| 66 | Mean | 1 | 500 | 0.3 | 0.8662 | 0.8510 |
| 67 | 0 | 1 | 500 | 0.5 | 0.8612 | 0.8480 |
| 68 | 1 | 1 | 500 | 0.5 | 0.8706 | 0.8459 |
| 69 | 2 | 1 | 500 | 0.5 | 0.8643 | 0.8551 |
| 70 | 3 | 1 | 500 | 0.5 | 0.8648 | 0.8471 |
| 71 | 4 | 1 | 500 | 0.5 | 0.8652 | 0.8551 |
| 72 | Mean | 1 | 500 | 0.5 | 0.8652 | 0.8502 |
| 73 | 0 | 2 | 100 | 0 | 0.8777 | 0.8593 |
| 74 | 1 | 2 | 100 | 0 | 0.8741 | 0.8592 |
| 75 | 2 | 2 | 100 | 0 | 0.8646 | 0.8523 |
| 76 | 3 | 2 | 100 | 0 | 0.8716 | 0.8603 |
| 77 | 4 | 2 | 100 | 0 | 0.8602 | 0.8613 |
| 78 | Mean | 2 | 100 | 0 | 0.8696 | 0.8585 |
| 79 | 0 | 2 | 100 | 0.1 | 0.8752 | 0.8568 |
| 80 | 1 | 2 | 100 | 0.1 | 0.8811 | 0.8541 |
| 81 | 2 | 2 | 100 | 0.1 | 0.8591 | 0.8553 |
| 82 | 3 | 2 | 100 | 0.1 | 0.8720 | 0.8521 |
| 83 | 4 | 2 | 100 | 0.1 | 0.8660 | 0.8594 |
| 84 | Mean | 2 | 100 | 0.1 | 0.8707 | 0.8555 |
| 85 | 0 | 2 | 100 | 0.3 | 0.8704 | 0.8623 |
| 86 | 1 | 2 | 100 | 0.3 | 0.8748 | 0.8494 |
| 87 | 2 | 2 | 100 | 0.3 | 0.8618 | 0.8530 |
| 88 | 3 | 2 | 100 | 0.3 | 0.8644 | 0.8514 |
| 89 | 4 | 2 | 100 | 0.3 | 0.8651 | 0.8560 |
| 90 | Mean | 2 | 100 | 0.3 | 0.8673 | 0.8544 |
| 91 | 0 | 2 | 100 | 0.5 | 0.8650 | 0.8502 |
| 92 | 1 | 2 | 100 | 0.5 | 0.8661 | 0.8423 |
| 93 | 2 | 2 | 100 | 0.5 | 0.8581 | 0.8481 |
| 94 | 3 | 2 | 100 | 0.5 | 0.8602 | 0.8432 |
| 95 | 4 | 2 | 100 | 0.5 | 0.8627 | 0.8465 |
| 96 | Mean | 2 | 100 | 0.5 | 0.8624 | 0.8461 |
| 97 | 0 | 2 | 300 | 0 | 0.8711 | 0.8604 |
| 98 | 1 | 2 | 300 | 0 | 0.8821 | 0.8620 |
| 99 | 2 | 2 | 300 | 0 | 0.8748 | 0.8529 |
| 100 | 3 | 2 | 300 | 0 | 0.8609 | 0.8542 |
| 101 | 4 | 2 | 300 | 0 | 0.8571 | 0.8606 |
| 102 | Mean | 2 | 300 | 0 | 0.8692 | 0.8580 |
| 103 | 0 | 2 | 300 | 0.1 | 0.8716 | 0.8574 |
| 104 | 1 | 2 | 300 | 0.1 | 0.8752 | 0.8557 |
| 105 | 2 | 2 | 300 | 0.1 | 0.8642 | 0.8620 |
| 106 | 3 | 2 | 300 | 0.1 | 0.8730 | 0.8568 |
| 107 | 4 | 2 | 300 | 0.1 | 0.8610 | 0.8582 |
| 108 | Mean | 2 | 300 | 0.1 | 0.8690 | 0.8580 |
| 109 | 0 | 2 | 300 | 0.3 | 0.8708 | 0.8613 |
| 110 | 1 | 2 | 300 | 0.3 | 0.8735 | 0.8595 |
| 111 | 2 | 2 | 300 | 0.3 | 0.8703 | 0.8548 |
| 112 | 3 | 2 | 300 | 0.3 | 0.8645 | 0.8586 |
| 113 | 4 | 2 | 300 | 0.3 | 0.8610 | 0.8625 |
| 114 | Mean | 2 | 300 | 0.3 | 0.8680 | 0.8594 |
| 115 | 0 | 2 | 300 | 0.5 | 0.8656 | 0.8608 |
| 116 | 1 | 2 | 300 | 0.5 | 0.8708 | 0.8546 |
| 117 | 2 | 2 | 300 | 0.5 | 0.8663 | 0.8586 |
| 118 | 3 | 2 | 300 | 0.5 | 0.8651 | 0.8511 |
| 119 | 4 | 2 | 300 | 0.5 | 0.8668 | 0.8594 |
| 120 | Mean | 2 | 300 | 0.5 | 0.8669 | 0.8569 |
| 121 | 0 | 2 | 500 | 0 | 0.8667 | 0.8575 |
| 122 | 1 | 2 | 500 | 0 | 0.8746 | 0.8638 |
| 123 | 2 | 2 | 500 | 0 | 0.8728 | 0.8554 |
| 124 | 3 | 2 | 500 | 0 | 0.8588 | 0.8582 |
| 125 | 4 | 2 | 500 | 0 | 0.8573 | 0.8589 |
| 126 | Mean | 2 | 500 | 0 | 0.8660 | 0.8587 |
| 127 | 0 | 2 | 500 | 0.1 | 0.8674 | 0.8591 |
| 128 | 1 | 2 | 500 | 0.1 | 0.8734 | 0.8568 |
| 129 | 2 | 2 | 500 | 0.1 | 0.8666 | 0.8577 |
| 130 | 3 | 2 | 500 | 0.1 | 0.8687 | 0.8590 |
| 131 | 4 | 2 | 500 | 0.1 | 0.8495 | 0.8612 |
| 132 | Mean | 2 | 500 | 0.1 | 0.8651 | 0.8588 |
| 133 | 0 | 2 | 500 | 0.3 | 0.8648 | 0.8605 |
| 134 | 1 | 2 | 500 | 0.3 | 0.8737 | 0.8559 |
| 135 | 2 | 2 | 500 | 0.3 | 0.8651 | 0.8593 |
| 136 | 3 | 2 | 500 | 0.3 | 0.8661 | 0.8500 |
| 137 | 4 | 2 | 500 | 0.3 | 0.8706 | 0.8578 |
| 138 | Mean | 2 | 500 | 0.3 | 0.8681 | 0.8567 |
| 139 | 0 | 2 | 500 | 0.5 | 0.8627 | 0.8581 |
| 140 | 1 | 2 | 500 | 0.5 | 0.8793 | 0.8500 |
| 141 | 2 | 2 | 500 | 0.5 | 0.8570 | 0.8472 |
| 142 | 3 | 2 | 500 | 0.5 | 0.8646 | 0.8569 |
| 143 | 4 | 2 | 500 | 0.5 | 0.8652 | 0.8601 |
| 144 | Mean | 2 | 500 | 0.5 | 0.8657 | 0.8544 |
| 145 | 0 | 3 | 100 | 0 | 0.8624 | 0.8548 |
| 146 | 1 | 3 | 100 | 0 | 0.8713 | 0.8591 |
| 147 | 2 | 3 | 100 | 0 | 0.8618 | 0.8540 |
| 148 | 3 | 3 | 100 | 0 | 0.8637 | 0.8612 |
| 149 | 4 | 3 | 100 | 0 | 0.8526 | 0.8591 |
| 150 | Mean | 3 | 100 | 0 | 0.8623 | 0.8576 |
| 151 | 0 | 3 | 100 | 0.1 | 0.8567 | 0.8598 |
| 152 | 1 | 3 | 100 | 0.1 | 0.8714 | 0.8598 |
| 153 | 2 | 3 | 100 | 0.1 | 0.8634 | 0.8531 |
| 154 | 3 | 3 | 100 | 0.1 | 0.8609 | 0.8585 |
| 155 | 4 | 3 | 100 | 0.1 | 0.8552 | 0.8606 |
| 156 | Mean | 3 | 100 | 0.1 | 0.8615 | 0.8584 |
| 157 | 0 | 3 | 100 | 0.3 | 0.8723 | 0.8580 |
| 158 | 1 | 3 | 100 | 0.3 | 0.8809 | 0.8583 |
| 159 | 2 | 3 | 100 | 0.3 | 0.8601 | 0.8514 |
| 160 | 3 | 3 | 100 | 0.3 | 0.8676 | 0.8516 |
| 161 | 4 | 3 | 100 | 0.3 | 0.8632 | 0.8509 |
| 162 | Mean | 3 | 100 | 0.3 | 0.8688 | 0.8540 |
| 163 | 0 | 3 | 100 | 0.5 | 0.8645 | 0.8474 |
| 164 | 1 | 3 | 100 | 0.5 | 0.8673 | 0.8421 |
| 165 | 2 | 3 | 100 | 0.5 | 0.8540 | 0.8426 |
| 166 | 3 | 3 | 100 | 0.5 | 0.8588 | 0.8409 |
| 167 | 4 | 3 | 100 | 0.5 | 0.8713 | 0.8493 |
| 168 | Mean | 3 | 100 | 0.5 | 0.8631 | 0.8445 |
| 169 | 0 | 3 | 300 | 0 | 0.8598 | 0.8566 |
| 170 | 1 | 3 | 300 | 0 | 0.8671 | 0.8542 |
| 171 | 2 | 3 | 300 | 0 | 0.8667 | 0.8543 |
| 172 | 3 | 3 | 300 | 0 | 0.8661 | 0.8571 |
| 173 | 4 | 3 | 300 | 0 | 0.8485 | 0.8508 |
| 174 | Mean | 3 | 300 | 0 | 0.8616 | 0.8546 |
| 175 | 0 | 3 | 300 | 0.1 | 0.8692 | 0.8558 |
| 176 | 1 | 3 | 300 | 0.1 | 0.8717 | 0.8608 |
| 177 | 2 | 3 | 300 | 0.1 | 0.8659 | 0.8553 |
| 178 | 3 | 3 | 300 | 0.1 | 0.8661 | 0.8553 |
| 179 | 4 | 3 | 300 | 0.1 | 0.8605 | 0.8596 |
| 180 | Mean | 3 | 300 | 0.1 | 0.8667 | 0.8574 |
| 181 | 0 | 3 | 300 | 0.3 | 0.8679 | 0.8628 |
| 182 | 1 | 3 | 300 | 0.3 | 0.8815 | 0.8572 |
| 183 | 2 | 3 | 300 | 0.3 | 0.8646 | 0.8592 |
| 184 | 3 | 3 | 300 | 0.3 | 0.8728 | 0.8585 |
| 185 | 4 | 3 | 300 | 0.3 | 0.8625 | 0.8627 |
| 186 | Mean | 3 | 300 | 0.3 | 0.8698 | 0.8601 |
| 187 | 0 | 3 | 300 | 0.5 | 0.8736 | 0.8538 |
| 188 | 1 | 3 | 300 | 0.5 | 0.8729 | 0.8543 |
| 189 | 2 | 3 | 300 | 0.5 | 0.8620 | 0.8501 |
| 190 | 3 | 3 | 300 | 0.5 | 0.8713 | 0.8550 |
| 191 | 4 | 3 | 300 | 0.5 | 0.8690 | 0.8569 |
| 192 | Mean | 3 | 300 | 0.5 | 0.8698 | 0.8540 |
| 193 | 0 | 3 | 500 | 0 | 0.8720 | 0.8580 |
| 194 | 1 | 3 | 500 | 0 | 0.8697 | 0.8597 |
| 195 | 2 | 3 | 500 | 0 | 0.8701 | 0.8538 |
| 196 | 3 | 3 | 500 | 0 | 0.8639 | 0.8606 |
| 197 | 4 | 3 | 500 | 0 | 0.8535 | 0.8599 |
| 198 | Mean | 3 | 500 | 0 | 0.8659 | 0.8584 |
| 199 | 0 | 3 | 500 | 0.1 | 0.8654 | 0.8510 |
| 200 | 1 | 3 | 500 | 0.1 | 0.8738 | 0.8530 |
| 201 | 2 | 3 | 500 | 0.1 | 0.8630 | 0.8530 |
| 202 | 3 | 3 | 500 | 0.1 | 0.8645 | 0.8519 |
| 203 | 4 | 3 | 500 | 0.1 | 0.8515 | 0.8544 |
| 204 | Mean | 3 | 500 | 0.1 | 0.8636 | 0.8526 |
| 205 | 0 | 3 | 500 | 0.3 | 0.8669 | 0.8585 |
| 206 | 1 | 3 | 500 | 0.3 | 0.8862 | 0.8623 |
| 207 | 2 | 3 | 500 | 0.3 | 0.8565 | 0.8530 |
| 208 | 3 | 3 | 500 | 0.3 | 0.8654 | 0.8541 |
| 209 | 4 | 3 | 500 | 0.3 | 0.8617 | 0.8598 |
| 210 | Mean | 3 | 500 | 0.3 | 0.8674 | 0.8575 |
| 211 | 0 | 3 | 500 | 0.5 | 0.8686 | 0.8578 |
| 212 | 1 | 3 | 500 | 0.5 | 0.8753 | 0.8549 |
| 213 | 2 | 3 | 500 | 0.5 | 0.8719 | 0.8539 |
| 214 | 3 | 3 | 500 | 0.5 | 0.8697 | 0.8571 |
| 215 | 4 | 3 | 500 | 0.5 | 0.8659 | 0.8549 |
| 216 | Mean | 3 | 500 | 0.5 | 0.8703 | 0.8557 |
| 217 | 0 | 4 | 100 | 0 | 0.8630 | 0.8610 |
| 218 | 1 | 4 | 100 | 0 | 0.8769 | 0.8626 |
| 219 | 2 | 4 | 100 | 0 | 0.8641 | 0.8535 |
| 220 | 3 | 4 | 100 | 0 | 0.8742 | 0.8550 |
| 221 | 4 | 4 | 100 | 0 | 0.8555 | 0.8589 |
| 222 | Mean | 4 | 100 | 0 | 0.8667 | 0.8582 |
| 223 | 0 | 4 | 100 | 0.1 | 0.8646 | 0.8615 |
| 224 | 1 | 4 | 100 | 0.1 | 0.8675 | 0.8550 |
| 225 | 2 | 4 | 100 | 0.1 | 0.8664 | 0.8559 |
| 226 | 3 | 4 | 100 | 0.1 | 0.8685 | 0.8519 |
| 227 | 4 | 4 | 100 | 0.1 | 0.8534 | 0.8560 |
| 228 | Mean | 4 | 100 | 0.1 | 0.8641 | 0.8561 |
| 229 | 0 | 4 | 100 | 0.3 | 0.8650 | 0.8540 |
| 230 | 1 | 4 | 100 | 0.3 | 0.8729 | 0.8496 |
| 231 | 2 | 4 | 100 | 0.3 | 0.8646 | 0.8502 |
| 232 | 3 | 4 | 100 | 0.3 | 0.8583 | 0.8520 |
| 233 | 4 | 4 | 100 | 0.3 | 0.8666 | 0.8539 |
| 234 | Mean | 4 | 100 | 0.3 | 0.8655 | 0.8520 |
| 235 | 0 | 4 | 100 | 0.5 | 0.8671 | 0.8461 |
| 236 | 1 | 4 | 100 | 0.5 | 0.8651 | 0.8410 |
| 237 | 2 | 4 | 100 | 0.5 | 0.8621 | 0.8450 |
| 238 | 3 | 4 | 100 | 0.5 | 0.8599 | 0.8447 |
| 239 | 4 | 4 | 100 | 0.5 | 0.8633 | 0.8498 |
| 240 | Mean | 4 | 100 | 0.5 | 0.8635 | 0.8453 |
| 241 | 0 | 4 | 300 | 0 | 0.8666 | 0.8605 |
| 242 | 1 | 4 | 300 | 0 | 0.8685 | 0.8545 |
| 243 | 2 | 4 | 300 | 0 | 0.8703 | 0.8570 |
| 244 | 3 | 4 | 300 | 0 | 0.8656 | 0.8568 |
| 245 | 4 | 4 | 300 | 0 | 0.8436 | 0.8614 |
| 246 | Mean | 4 | 300 | 0 | 0.8629 | 0.8581 |
| 247 | 0 | 4 | 300 | 0.1 | 0.8656 | 0.8585 |
| 248 | 1 | 4 | 300 | 0.1 | 0.8789 | 0.8571 |
| 249 | 2 | 4 | 300 | 0.1 | 0.8628 | 0.8555 |
| 250 | 3 | 4 | 300 | 0.1 | 0.8607 | 0.8572 |
| 251 | 4 | 4 | 300 | 0.1 | 0.8475 | 0.8549 |
| 252 | Mean | 4 | 300 | 0.1 | 0.8631 | 0.8566 |
| 253 | 0 | 4 | 300 | 0.3 | 0.8732 | 0.8636 |
| 254 | 1 | 4 | 300 | 0.3 | 0.8772 | 0.8578 |
| 255 | 2 | 4 | 300 | 0.3 | 0.8713 | 0.8592 |
| 256 | 3 | 4 | 300 | 0.3 | 0.8645 | 0.8539 |
| 257 | 4 | 4 | 300 | 0.3 | 0.8651 | 0.8654 |
| 258 | Mean | 4 | 300 | 0.3 | 0.8703 | 0.8600 |
| 259 | 0 | 4 | 300 | 0.5 | 0.8702 | 0.8587 |
| 260 | 1 | 4 | 300 | 0.5 | 0.8735 | 0.8507 |
| 261 | 2 | 4 | 300 | 0.5 | 0.8679 | 0.8561 |
| 262 | 3 | 4 | 300 | 0.5 | 0.8685 | 0.8543 |
| 263 | 4 | 4 | 300 | 0.5 | 0.8676 | 0.8581 |
| 264 | Mean | 4 | 300 | 0.5 | 0.8695 | 0.8556 |
| 265 | 0 | 4 | 500 | 0 | 0.8645 | 0.8560 |
| 266 | 1 | 4 | 500 | 0 | 0.8686 | 0.8647 |
| 267 | 2 | 4 | 500 | 0 | 0.8655 | 0.8546 |
| 268 | 3 | 4 | 500 | 0 | 0.8618 | 0.8544 |
| 269 | 4 | 4 | 500 | 0 | 0.8578 | 0.8555 |
| 270 | Mean | 4 | 500 | 0 | 0.8636 | 0.8571 |
| 271 | 0 | 4 | 500 | 0.1 | 0.8646 | 0.8638 |
| 272 | 1 | 4 | 500 | 0.1 | 0.8681 | 0.8555 |
| 273 | 2 | 4 | 500 | 0.1 | 0.8675 | 0.8491 |
| 274 | 3 | 4 | 500 | 0.1 | 0.8729 | 0.8548 |
| 275 | 4 | 4 | 500 | 0.1 | 0.8521 | 0.8527 |
| 276 | Mean | 4 | 500 | 0.1 | 0.8651 | 0.8552 |
| 277 | 0 | 4 | 500 | 0.3 | 0.8623 | 0.8567 |
| 278 | 1 | 4 | 500 | 0.3 | 0.8791 | 0.8590 |
| 279 | 2 | 4 | 500 | 0.3 | 0.8652 | 0.8559 |
| 280 | 3 | 4 | 500 | 0.3 | 0.8643 | 0.8537 |
| 281 | 4 | 4 | 500 | 0.3 | 0.8698 | 0.8598 |
| 282 | Mean | 4 | 500 | 0.3 | 0.8682 | 0.8570 |
| 283 | 0 | 4 | 500 | 0.5 | 0.8647 | 0.8593 |
| 284 | 1 | 4 | 500 | 0.5 | 0.8755 | 0.8527 |
| 285 | 2 | 4 | 500 | 0.5 | 0.8618 | 0.8529 |
| 286 | 3 | 4 | 500 | 0.5 | 0.8651 | 0.8578 |
| 287 | 4 | 4 | 500 | 0.5 | 0.8647 | 0.8552 |
| 288 | Mean | 4 | 500 | 0.5 | 0.8664 | 0.8556 |
| 289 | 0 | 5 | 100 | 0 | 0.8580 | 0.8555 |
| 290 | 1 | 5 | 100 | 0 | 0.8718 | 0.8591 |
| 291 | 2 | 5 | 100 | 0 | 0.8652 | 0.8582 |
| 292 | 3 | 5 | 100 | 0 | 0.8716 | 0.8530 |
| 293 | 4 | 5 | 100 | 0 | 0.8511 | 0.8534 |
| 294 | Mean | 5 | 100 | 0 | 0.8635 | 0.8558 |
| 295 | 0 | 5 | 100 | 0.1 | 0.8653 | 0.8607 |
| 296 | 1 | 5 | 100 | 0.1 | 0.8742 | 0.8599 |
| 297 | 2 | 5 | 100 | 0.1 | 0.8660 | 0.8529 |
| 298 | 3 | 5 | 100 | 0.1 | 0.8697 | 0.8681 |
| 299 | 4 | 5 | 100 | 0.1 | 0.8622 | 0.8540 |
| 300 | Mean | 5 | 100 | 0.1 | 0.8675 | 0.8591 |
| 301 | 0 | 5 | 100 | 0.3 | 0.8669 | 0.8481 |
| 302 | 1 | 5 | 100 | 0.3 | 0.8763 | 0.8451 |
| 303 | 2 | 5 | 100 | 0.3 | 0.8613 | 0.8499 |
| 304 | 3 | 5 | 100 | 0.3 | 0.8658 | 0.8530 |
| 305 | 4 | 5 | 100 | 0.3 | 0.8728 | 0.8547 |
| 306 | Mean | 5 | 100 | 0.3 | 0.8686 | 0.8502 |
| 307 | 0 | 5 | 100 | 0.5 | 0.8570 | 0.8408 |
| 308 | 1 | 5 | 100 | 0.5 | 0.8613 | 0.8343 |
| 309 | 2 | 5 | 100 | 0.5 | 0.8608 | 0.8426 |
| 310 | 3 | 5 | 100 | 0.5 | 0.8481 | 0.8381 |
| 311 | 4 | 5 | 100 | 0.5 | 0.8651 | 0.8423 |
| 312 | Mean | 5 | 100 | 0.5 | 0.8585 | 0.8396 |
| 313 | 0 | 5 | 300 | 0 | 0.8723 | 0.8621 |
| 314 | 1 | 5 | 300 | 0 | 0.8741 | 0.8558 |
| 315 | 2 | 5 | 300 | 0 | 0.8593 | 0.8591 |
| 316 | 3 | 5 | 300 | 0 | 0.8555 | 0.8561 |
| 317 | 4 | 5 | 300 | 0 | 0.8567 | 0.8550 |
| 318 | Mean | 5 | 300 | 0 | 0.8636 | 0.8576 |
| 319 | 0 | 5 | 300 | 0.1 | 0.8606 | 0.8596 |
| 320 | 1 | 5 | 300 | 0.1 | 0.8680 | 0.8534 |
| 321 | 2 | 5 | 300 | 0.1 | 0.8684 | 0.8532 |
| 322 | 3 | 5 | 300 | 0.1 | 0.8642 | 0.8562 |
| 323 | 4 | 5 | 300 | 0.1 | 0.8631 | 0.8579 |
| 324 | Mean | 5 | 300 | 0.1 | 0.8649 | 0.8561 |
| 325 | 0 | 5 | 300 | 0.3 | 0.8711 | 0.8641 |
| 326 | 1 | 5 | 300 | 0.3 | 0.8826 | 0.8592 |
| 327 | 2 | 5 | 300 | 0.3 | 0.8667 | 0.8543 |
| 328 | 3 | 5 | 300 | 0.3 | 0.8679 | 0.8505 |
| 329 | 4 | 5 | 300 | 0.3 | 0.8596 | 0.8538 |
| 330 | Mean | 5 | 300 | 0.3 | 0.8696 | 0.8564 |
| 331 | 0 | 5 | 300 | 0.5 | 0.8781 | 0.8597 |
| 332 | 1 | 5 | 300 | 0.5 | 0.8778 | 0.8520 |
| 333 | 2 | 5 | 300 | 0.5 | 0.8670 | 0.8491 |
| 334 | 3 | 5 | 300 | 0.5 | 0.8649 | 0.8511 |
| 335 | 4 | 5 | 300 | 0.5 | 0.8657 | 0.8556 |
| 336 | Mean | 5 | 300 | 0.5 | 0.8707 | 0.8535 |
| 337 | 0 | 5 | 500 | 0 | 0.8649 | 0.8584 |
| 338 | 1 | 5 | 500 | 0 | 0.8707 | 0.8518 |
| 339 | 2 | 5 | 500 | 0 | 0.8674 | 0.8504 |
| 340 | 3 | 5 | 500 | 0 | 0.8637 | 0.8589 |
| 341 | 4 | 5 | 500 | 0 | 0.8487 | 0.8561 |
| 342 | Mean | 5 | 500 | 0 | 0.8631 | 0.8551 |
| 343 | 0 | 5 | 500 | 0.1 | 0.8731 | 0.8597 |
| 344 | 1 | 5 | 500 | 0.1 | 0.8766 | 0.8585 |
| 345 | 2 | 5 | 500 | 0.1 | 0.8650 | 0.8513 |
| 346 | 3 | 5 | 500 | 0.1 | 0.8603 | 0.8564 |
| 347 | 4 | 5 | 500 | 0.1 | 0.8460 | 0.8594 |
| 348 | Mean | 5 | 500 | 0.1 | 0.8642 | 0.8571 |
| 349 | 0 | 5 | 500 | 0.3 | 0.8684 | 0.8549 |
| 350 | 1 | 5 | 500 | 0.3 | 0.8853 | 0.8497 |
| 351 | 2 | 5 | 500 | 0.3 | 0.8578 | 0.8562 |
| 352 | 3 | 5 | 500 | 0.3 | 0.8732 | 0.8592 |
| 353 | 4 | 5 | 500 | 0.3 | 0.8665 | 0.8591 |
| 354 | Mean | 5 | 500 | 0.3 | 0.8702 | 0.8558 |
| 355 | 0 | 5 | 500 | 0.5 | 0.8698 | 0.8495 |
| 356 | 1 | 5 | 500 | 0.5 | 0.8825 | 0.8532 |
| 357 | 2 | 5 | 500 | 0.5 | 0.8659 | 0.8520 |
| 358 | 3 | 5 | 500 | 0.5 | 0.8634 | 0.8539 |
| 359 | 4 | 5 | 500 | 0.5 | 0.8656 | 0.8612 |
| 360 | Mean | 5 | 500 | 0.5 | 0.8694 | 0.8539 |

The first column represents the number of rows. The second column represents the number of fold that is used as test dataset. The third, fourth and firth columns represent the parameters of Deep Learning, the number of hidden layers, the number of neurons in one layer and dropout rate. The sixth and seventh columns represents the AUC value in different datasets.
